# The Butterfly Effect: Multifaceted Consequences of Sensitizer Concentration Change in Phase Transition-based Luminescent Thermometer of LiYO_2_:Er^3+^,Yb^3+^

**DOI:** 10.1021/acsami.4c03856

**Published:** 2024-05-13

**Authors:** L. Marciniak, W. Piotrowski, M. Szymczak, C. D. S. Brites, V. Kinzhybalo, Hao Suo, L.D. Carlos, Feng Wang

**Affiliations:** † Institute of Low Temperature and Structure Research, Polish Academy of Sciences, Okolna 2, 50-422 Wroclaw, Poland; ‡ Physics Department and CICECO—Aveiro Institute of Materials, University of Aveiro, 3810-193 Aveiro, Portugal; § Department of Materials Science and Engineering, City University of Hong Kong, Kowloon, Hong Kong

**Keywords:** phase transition, luminescent thermometry, relative sensitivity, Er^3+^, Yb^3+^, upconversion

## Abstract

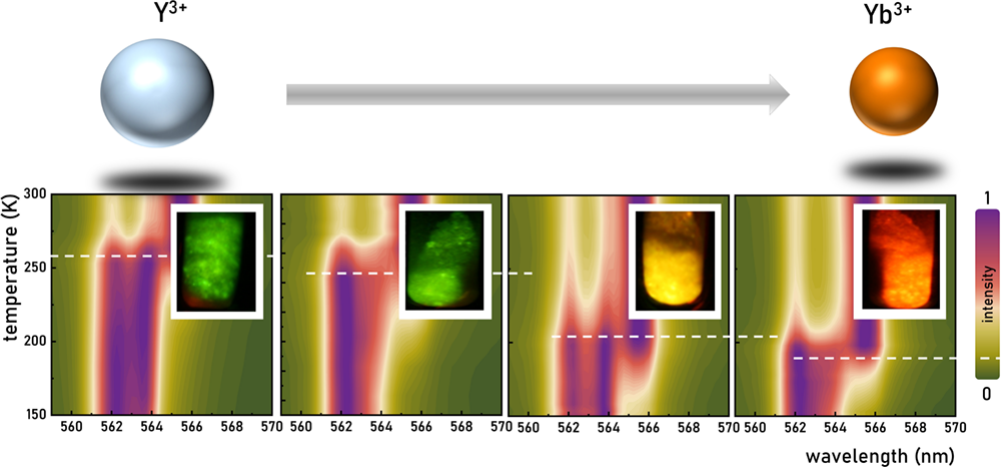

In response to the ongoing quest for new, highly sensitive upconverting luminescent thermometers, this article introduces, for the first time, upconverting luminescent thermometers based on thermally induced structured phase transitions. As demonstrated, the transition from the low-temperature monoclinic to the high-temperature tetragonal structures of LiYO_2_:Yb^3+^,Er^3+^ induces multifaceted modification in the spectroscopic properties of the examined material, influencing the spectral positions of luminescence bands, energy gap values between thermally coupled energy levels, and the red-to-green emission intensities ratio. Moreover, as illustrated, both the color of the emitted light and the phase transition temperature (from 265 K, for LiYO_2_:Er^3+^, 1%Yb^3+^, to 180 K, for 10%Yb^3+^), and consequently, the thermometric parameters of the luminescent thermometer can be modulated by the concentration of Yb^3+^ sensitizer ions. Establishing a correlation between the phase transition temperature and the mismatch of ion radii between the host material and dopant ions allows for smooth adjustment of the thermometric performance of such a thermometer following specific application requirements. Three different thermometric approaches were investigated using thermally coupled levels (*S*_R_ = 1.8%/K at 180 K for 1%Yb^3+^), green to red emission intensities ratio (*S*_R_ = 1.5%/K at 305 K for 2%Yb^3+^), and single band ratiometric approach (*S*_R_ = 2.5%/K at 240 K for 10%Yb^3+^). The thermally induced structural phase transition in LiYO_2_:Er^3+^,Yb^3+^ has enabled the development of multiple upconverting luminescent thermometers. This innovative approach opens avenues for advancing the field of luminescence thermometry, offering enhanced relative thermal sensitivity and adaptability for various applications.

## Introduction

The recent surge in luminescence thermometry is primarily propelled by the possibility of remote, electrically passive temperature measurements, and its methodological simplicity.^1−4^ The precision and relative sensitivity of temperature measurement in this context are contingent upon the rate of the thermal changes in the spectroscopic parameters of the phosphor and of the detection setup employed.^5−13^ Therefore, current research endeavors are accordingly focused on delineating new processes and proposing strategic methodologies to optimize the performance of luminescent thermometers.^8^ A particularly innovative idea proposed recently involves exploiting changes in the spectroscopic properties due to thermally induced reversible structural transitions of the luminescent material.^14−17^ The spectroscopic properties of luminescent ions, e.g., are determined by their structural environment, encompassing parameters such as coordination number, point symmetry, and covalency of bonds.^18^ In the case of the trivalent lanthanide ions, the shielding of 4f orbital from the environment by the 5s6p orbitals results in the small sensitivity of their spectroscopic properties to the structural changes. Despite that, some alterations in the crystallographic environment can induce notable effects, including changes in the number of Stark components and the barycenter of emission bands. Such modifications facilitate additional energy transfer through resonance matching. Consequently, a thermally induced structural phase transition emerges as a pivotal modulator of the spectroscopic properties of the phosphor, paving the way for the development of a high-sensitivity luminescent thermometer.^14−17^ This advancement is achieved through a ratiometric temperature readout, where the thermometric parameter is defined by the ratio of the integrated intensity of the bands corresponding to individual structural phases. An excellent example of such host material is LiYO_2_, undergoing a structural phase transition from a monoclinic (low-temperature phase) to a tetragonal structure (high-temperature phase) around room temperature, resulting in an alteration of the point symmetry of the Y^3+^ ion from C_2_ to D_2d_.^19−23^ Previous studies have underscored the success of this strategic approach, yielding very high relative thermal sensitivities surpassing 10% K^–1^.

While this method has been extensively explored for Stokes emitting phosphors, the realm of phase transition-based upconverting luminescent thermometers remains relatively unexplored.^14−17^ This article introduces a comprehensive study of LiYO_2_:Er^3+^,Yb^3+^, a well-explored upconversion system. Diverging from the conventional reliance on thermal coupling between the ^2^H_11/2_ and ^4^S_3/2_ levels for temperature determination, the thermally induced phase transition in LiYO_2_:Er^3+^,Yb^3+^ elicits multifaceted changes in the spectroscopic properties. This versatility enables the development of multimodal ratiometric luminescent thermometers, thereby highlighting the nuanced and multifaceted attributes of LiYO_2_ as a luminescence-based temperature sensor.

## Experimental Section

### Synthesis

The powders of LiYO_2_:1%Er^3+^, *x*% Yb^3+^ (*x* = 1, 2, 5, 10) nanocrystals were synthesized with a modified Pechini method. Li_2_CO_3_ (99.9% purity, Chempur), Y_2_O_3_ (99.999% purity, Stanford Materials Corporation), Er_2_O_3_ (99.99% purity, Stanford Materials Corporation), Yb_2_O_3_ (99.99% purity, Stanford Materials Corporation), C_6_H_8_O_7_ (>99.5% purity, Alfa Aesar), and H(OCH_2_CH_2_)_*n*_OH, (PEG-200, *n* = 200, Alfa Aesar) were used as starting materials. Yttrium, ytterbium, and erbium oxides were dissolved in deionized water with the addition of a small amount of HNO_3_ (65% purity, Avantor) and then recrystallized three times to remove the excess nitrogen. The 4-fold stoichiometric excess of lithium carbonate was added to the water solution of nitrates. After that, anhydrous citric acid and polyglycol were added to the mixture. The molar ratio of citric acid to all metals was set up as 6:1. Meanwhile, PEG-200 and citric acid were used in a molar ratio of 1:1. Subsequently, the obtained solution was dried for 3 days at 363 K until a resin was formed. The produced resin of the samples with 1% Er^3+^ and *x*% Yb^3+^ (*x* = 1, 2, 5, 10) concentration with respect to the number of Y^3+^ moles ions was annealed in porcelain crucibles for 6 h in the air at a temperature of 1273 K.

### Characterization

All of the synthesized materials were examined by powder X-ray diffraction measurements. Powder diffraction data were obtained in Bragg–Brentano geometry using a PANalytical X’Pert Pro diffractometer equipped with Oxford Cryosystems Phenix low-temperature and Anton Paar HTK 1200N high-temperature attachments using Ni-filtered Cu Kα radiation (*V* = 40 kV, *I* = 30 mA). The sample for the low-temperature diffraction experiment was mixed with an Apiezon grease. Diffraction patterns in the 15–90° 2θ range were measured in a cooling/heating sequence in the 100–325 K temperature range. ICSD database entries 50992 (low-temperature phase) and 50993 (high-temperature phase) were taken as initial models for the analysis of the obtained diffraction data.

Transmission electron microscopy (TEM) images were performed with a Philips CM-20 SuperTwin transmission electron microscope, operating at 160 kV. The sample was ground in a mortar and dispersed in methanol, and then a drop of the suspension was put on a copper microscope grid covered with carbon.

The excitation spectra were obtained using the FLS1000 Fluorescence Spectrometer from Edinburgh Instruments, which was equipped with a 450 W xenon lamp as an excitation source and R928 photomultiplier tube from Hamamatsu as a detector. Emission spectra were measured using the same system with 975 and 980 nm laser diodes as excitation source. To carry out the temperature-dependent measurement, the temperature of the sample was controlled by using a THMS600 heating–cooling stage from Linkam (0.1 K temperature stability and 0.1 K set point resolution). Luminescence decay profiles were recorded using the FLS1000 Fluorescence Spectrometer from Edinburgh Instruments equipped with a R928 photomultiplier tube from Hamamatsu and 980 nm pulsed work laser diode as excitation source. The average lifetime of the excited states was calculated with a fit to a double-exponential function (eq 1):
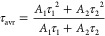
1where τ_1_, τ_2_ are the decay parameters and *A*_1_, *A*_2_ are the fitted amplitudes of the biexponential function (eq 2):
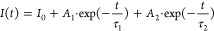
2

The absolute photoluminescence quantum yield (QY) of all samples was measured by using a Quantaurus-QY (C13534, Hamamatsu) system. This setup features a 150 W xenon lamp coupled to a monochromator for excitation wavelength selection, an integrating sphere as the sample chamber, and two multichannel analyzers for simultaneous visible and near-infrared (NIR) spectral detection. An external 975 nm laser diode (FC-980 5W, CNI Lasers) served as the excitation source, with its power ranging from 0.63 to 6.4 W (corresponding to a laser power density between 250 and 2564 W cm^–2^). All measurements were performed in quartz sample holders, with an empty holder used as the reference. The emission QY was calculated as the ratio of emitted to absorbed photons. The system software automatically computes the emission QY based on user-defined wavelength integration ranges for both excitation and emission spectra. In the emission spectral range, both downshifting (1007–1650 nm) and upconverting (483–920 nm) emissions were recorded. Three independent measurements were performed for each sample, and the reported value represents the average. According to the manufacturer, the measurements have a relative error of 10%.

## Results and Discussion

**Figure 1:**
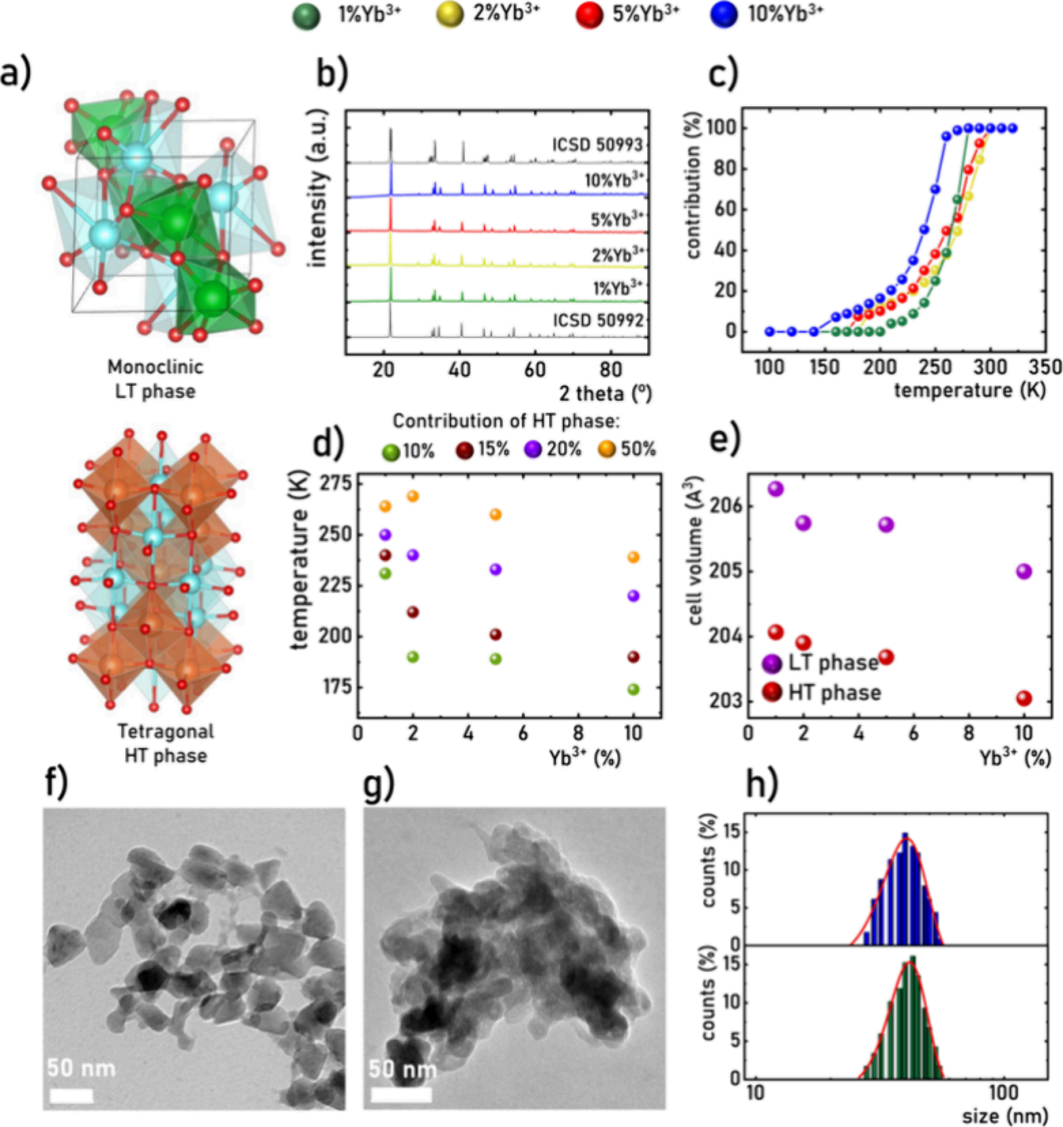
Structure of the low temperature (LT) monoclinic phase and high temperature (HT) tetragonal phase of LiYO_2_ (a); comparison of the room temperature XRD for LiYO_2_:Yb^3+^,Er^3+^ with different concentration of Yb^3+^ ions (b); thermal dependence of the contribution of HT phase in the diffractograms of LiYO_2_:Yb^3+^,Er^3+^ measured as a function of temperature for different Yb^3+^ concentration (c); the temperature at which the particular contributions of HT phase were found in the diffractograms for different Yb^3+^ concentration (d); the influence of Yb^3+^ concentration on the unit cell volume of the LT and HT phases of LiYO_2_ (e); the representative TEM images of LiYO_2_:1%Er^3+^, 1%Yb^3+^ (f); and LiYO_2_:1%Er^3+^, 10%Yb^3+^ (g) and corresponding particle size distributions (40.4 nm and fwhm = 19.5, *r*^2^ > 0.98 for LiYO_2_:1%Er^3+^, 1%Yb^3+^ and 41.6 nm and fwhm = 22.9 nm, *r*^2^ > 0.97 for LiYO_2_:1%Er^3+^, 10%Yb^3+^) (h).

LiYO_2_ crystallizes in two crystallographic phases: a low-temperature monoclinic phase (*P21/c* space group, LT) and a high-temperature tetragonal phase (*I41/amd* space group, HT) (Figure 1a).^19−23^ The phase transition temperature significantly depends on the material’s morphology (in the case of LiYO_2_ single crystal, the phase transition was found at 373 K, while for nanomaterial with an average particle size of ∼50 nm, it occurs around 293 K), and the type and concentration of dopant ions.^14,22^ Regardless of the temperature, the structural transition results in a change in the size of the unit cell from *a* = 6.1493(8) Å, *b* = 6.1500(10) Å, *c* = 6.2494(2) Å, β = 119.091(5)° to *a* = 4.4468(9) Å, *c* = 10.372(22) Å and the point symmetry of Y^3+^ ions from *C*_*2*_ to *D*_*2d*_. The change in the point symmetry of the Y^3+^ cation is particularly significant as lanthanide dopant ions substitute for the Y^3+^ cation, and the point symmetry significantly modifies the spectroscopic properties of the dopant. Comparison of room temperature X-ray diffraction (XRD) patterns measured for LiYO_2_:1%Er^3+^, Yb^3+^ for different Yb^3+^ ion concentrations indicates that they are dominated by the high-temperature tetragonal phase (Figure 1b, see also Figure S1). Additionally, the introduction of Yb^3+^ ions does not generate the appearance of additional reflections, confirming the phase purity of the material and the effective substitution of Y^3+^ ions by Yb^3+^ ions. The in situ temperature-dependent XRD measurements enabled, through the Rietveld refinement method, the determination of the contribution of individual structures to the obtained diffractogram (Figure 1c, results of the calculation can be found in the SI and calculated values of the cell parameters as a function of temperature are presented in Figure S2). An increase in the temperature favors an increase in the content of the tetragonal LiYO_2_ phase for all of the analyzed materials. However, the concentration of Yb^3+^ significantly affects the pace of the observed changes. As a general tendency, one can observe that the higher the concentration of Yb^3+^ ions, the lower the temperature at which the reflections from the HT phase are observed. This correlation was illustrated in Figure 1d, where the temperature dependency for which the HT content reaches 10, 15, 20, and 50%, respectively, is plotted as a function of Yb^3+^ ion concentration (Figure 1e). The observed effect is related to the difference in ionic radii between the host material cation Y^3+^ (*R* = 1.019 Å) and the dopant ion Yb^3+^ (*R* = 0.985 Å), which will be discussed further in the paper.^24^ This causes a gradual reduction in the unit cell size observed for both the LT phase (calculation performed for the XRD patterns measured at 100 K) and HT (measured at 310 K) with increasing Yb^3+^ concentration. However, as indicated by the analysis of the TEM images obtained for two extreme concentrations of Yb^3+^ (1% in Figure 1f and 10% in Figure 1g), an increase in dopant ion concentration does not cause significant changes in the material’s morphology and the distribution of crystallite sizes. It is worth noting that the obtained materials consist of strongly aggregated crystallites with an average size of about 42 ± 12 nm (Figure 1h).

### Doping Concentration Dependence of Upconversion

**Figure 2:**
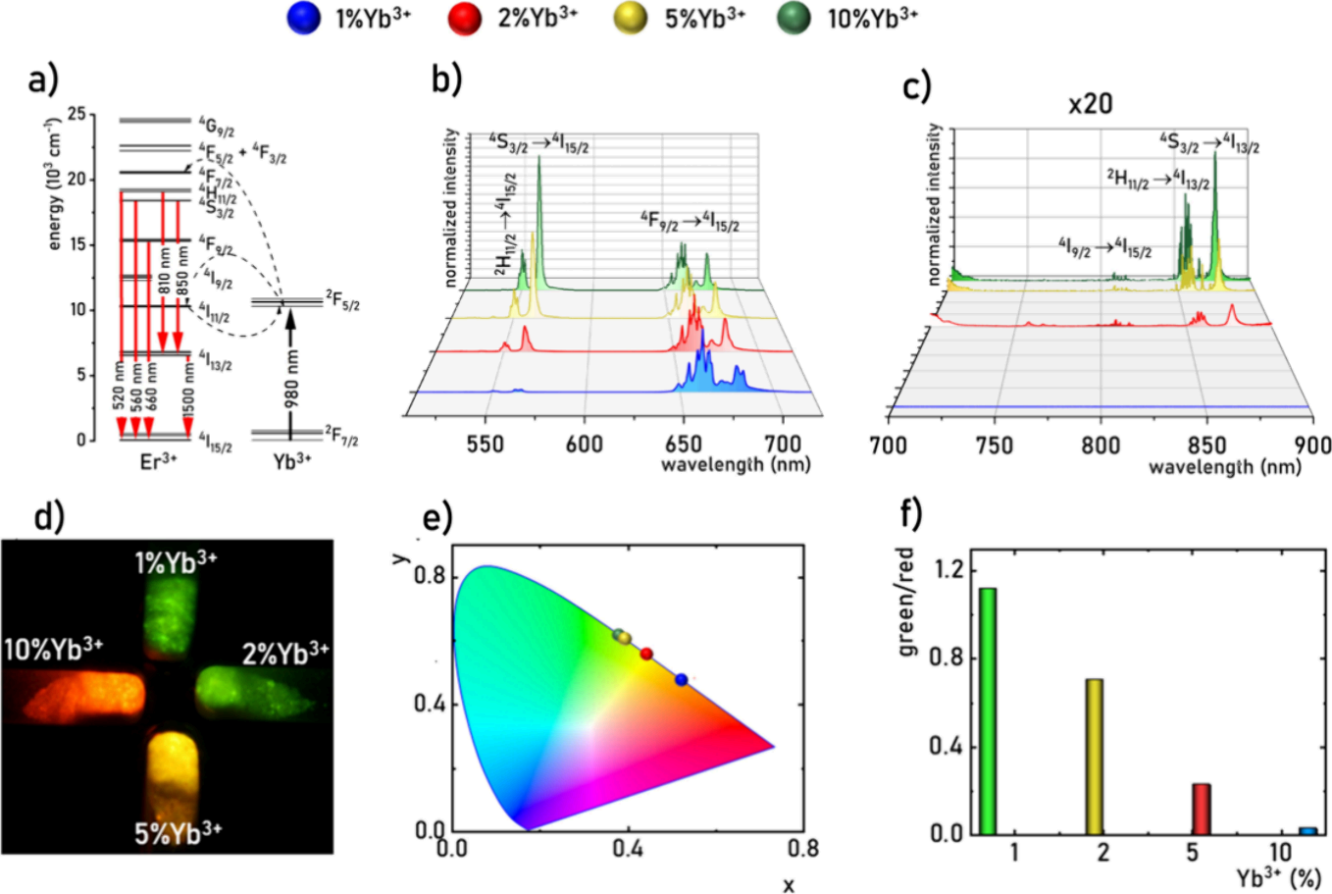
Simplified energy level diagrams of Er^3+^ and Yb^3+^ ions (a) the comparison of room temperature upconversion emission spectra of LiYO_2_:Er^3+^,Yb^3+^ with different concentrations of Yb^3+^ ions upon λ_exc_ = 975 nm) in the visible (b); and NIR (c) spectral ranges; photographs presenting the room temperature upconversion luminescence of LiYO_2_:Er^3+^,Yb^3+^ powders (d); the chromatic coordinates of the emitted light (CIE 1931) (e); green to red emission intensity ratio as a function of Yb^3+^ concentration (f).

The upconversion emission in the Er^3+^, Yb^3+^ pair system is one of the most extensively studied energy transfer processes.^25−29^ Therefore, the following description focuses on its salient aspects (Figure 2a). In this configuration, Yb^3+^ ions act as sensitizers, absorbing excitation energy of 975 nm pumping wavelength and subsequently transferring it to Er^3+^ ions, acting as acceptors (excitation spectra can be found in Figure S3). This energy transfer is made possible by perfect energy matching between the ^2^F_5/2_ and ^2^F_7/2_ levels of Yb^3+^ ions with the energy difference between the ^4^I_15/2_ and ^4^I_9/2_ states of Er^3+^ions. The utilization of Yb^3+^ ions with a substantially higher absorption cross-section at 975 nm than the Er^3+^ counterpart enhances the upconversion intensity of Er^3+^ ions, optimizing the absorption of excitation photons. Upon energy transfer to Er^3+^ ions, the ^4^I_9/2_ level becomes populated, and the ensuing Yb^3+^ → Er^3+^ energy transfer process facilitates the population of the ^4^F_7/2_ state. Subsequent multiphonon depopulation processes lead to the occupation of the ^2^H_11/2_, ^4^S_3/2_ and ^4^F_9/2_ levels, resulting, through their radiative depopulation, in the generation of emission bands in the green (510–550 nm for ^2^H_11/2_ → ^4^I_15/2_ and 550–590 nm for ^4^S_3/2_ → ^4^I_15/2_) and red (650–690 nm for ^4^F_9/2_ → ^4^I_15/2_) spectral ranges of the spectrum (Figures 2b and S4). Furthermore, what is uncommonly observed for Er^3+^-based upconversion phosphors, the emission bands associated with electronic transitions ^2^H_11/2_ → ^4^I_13/2_ and ^4^S_3/2_ → ^4^I_13/2_ are noted in the NIR range of LiYO_2_:Er^3+^, Yb^3+^ (Figure 2c). While their intensity is around 20-fold less intense than counterparts to the ^4^I_15/2_ level, their presence holds significance for the development of NIR-to-NIR upconversion phosphors, particularly valuable for background-free bioimaging applications.

The energy transfer probability between Yb^3+^ and Er^3+^ is inversely proportional to the interionic distance between them. Consequently, higher molar concentrations of Yb^3+^ ions (typically ten times higher than the acceptors) are conventionally used. Therefore, the influence of the Yb^3+^ ion concentration on the spectroscopic properties of LiYO_2_:Er^3+^,Yb^3+^ was systematically examined. The comparison of room temperature upconversion emission spectra with varying concentrations of sensitizer ions reveals a decrease in luminescence intensity of the ^2^H_11/2_ → ^4^I_15/2_ and ^4^S_3/2_ → ^4^I_15/2_ transitions in the green spectral range relative to the red ^4^F_9/2_ → ^4^I_15/2_ band with increasing Yb^3+^ concentration. This manifests in a notable shift in emitted light color from green (1% Yb^3+^) through yellowish (5% Yb^3+^) and eventually to orange luminescence (10% Yb^3+^) (Figure 2d,e). The green-to-red emission intensity ratio decreases by a factor of 10 as the Yb^3+^ concentration increases from 1 to 10% (Figure 2f). This quenching of luminescence from the ^2^H_11/2_ and ^4^S_3/2_ levels may be attributed to back Er^3+^ → Yb^3+^ energy transfer, with the probability increasing as the distance between the sensitizer and acceptor shortens. This hypothesis is supported by the kinetics analysis of luminescence from the ^4^S_3/2_ level, where an increase in sensitizer concentration results in a shortened average lifetime from τ_avr_ = 0.340 ± 0.01 ms to 0.150 ± 0.01 ms (Figures S5 and S6). This elucidates that the emission color output of the upconverting LiYO_2_:Er^3+^,Yb^3+^ can be easily tailored by adjusting the concentration of sensitizer ions.

### Temperature Dependence of Upconversion

**Figure 3:**
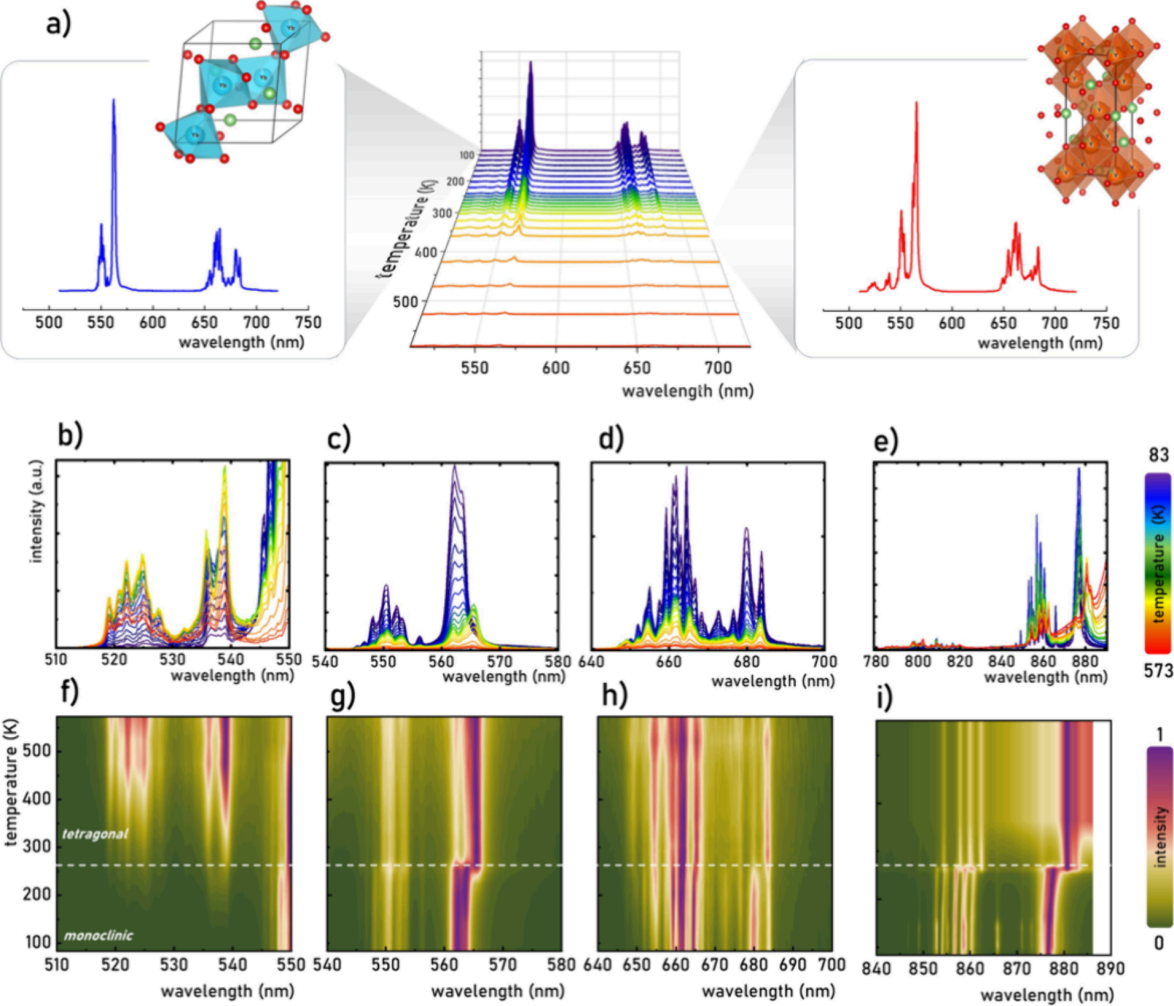
Upconversion emission spectra of LiYO_2_:Er^3+^,1%Yb^3+^ measured as a function of temperature upon λ_exc_ = 975 nm with the indication of the emission spectra characteristic for monoclinic and tetragonal phases of LiYO_2_:Er^3+^,Yb^3+^ (a) the limited to the ^2^H_11/2_→ ^4^I_15/2_ (b), ^4^S_3/2_ → ^4^I_15/2_ (c), ^4^F_9/2_ → ^4^I_15/2_ (d), ^2^H_11/2_ → ^4^I_13/2_ and ^4^S_3/2_ → ^4^I_13/2_ (e) emission bands; and the corresponding luminescence maps of normalized emission spectra (f–i).

To assess the potential application of LiYO_2_:Er^3+^,Yb^3+^ in luminescence thermometry, the upconversion emission spectra were measured in the 83–573 K range (Figure 3a). The total luminescence intensity experiences pronounced quenching with increasing temperature arising from two factors: the increasing probability of nonradiative multiphonon depopulation of emitting levels at higher temperatures and the spectrally narrow absorption band of Yb^3+^ ions in LiYO_2_:Er^3+^, Yb^3+^, which undergoes a temperature-induced spectral shift, causing a spectral mismatch with the excitation wavelength. Consequently, beyond 473 K, the emission intensity undergoes substantial attenuation due to reduced excitation efficiency. Therefore, the temperature increase induces significant changes in the emission spectrum related to the structural phase transition between monoclinic and tetragonal phases. These alterations are discernible in each emission band, and for the sake of clarity, the bands are presented separately in Figure 3b–e. The temperature elevation prompts the appearance of emission bands around 525 and 538 nm, associated with electronic transitions ^4^F_3/2_ → ^4^I_15/2_ and ^4^F_7/2_ → ^4^I_15/2_, respectively. These bands result from the thermalization of the higher-lying energy levels with increasing temperature and are likely unrelated to the phase transition. However, for the ^2^H_11/2_ → ^4^I_15/2_ and ^4^S_3/2_ → ^4^I_15/2_ (Figure 3c), ^4^F_9/2_ → ^4^I_15/2_ (Figure 3d), and ^2^H_11/2_ → ^4^I_13/2_ and ^4^S_3/2_ → ^4^I_13/2_ (Figure 3e) transitions, a red-shift is observable, accompanied by a reduction in the number of Stark components with increasing temperature. This is corroborated by the luminescence maps of the normalized signals, revealing a distinct phase transition at approximately 290 K for LiYO_2_:Er^3+^,1%Yb^3+^ (Figure 3f–i). Notably, a further temperature increase beyond the phase transition point rapidly diminishes the luminescence intensity of the bands associated with the monoclinic phase. However, these bands are not completely quenched, suggesting the presence of a small fraction of low-temperature phases at temperatures above the phase transition point, attributed to the spectral overlap of signals from both phases (thermal dependence of τ_avr_ can be found in Figures S7 and S8).

The upconversion QY was measured for LiYO_2_:Er^3+^,Yb^3+^ as a function of the 975-nm laser power density. The results obtained were confronted with the QY of the Stokes part of the emission spectra corresponding to the ^4^I_13/2_ → ^4^I_15/2_ transition. As expected, an increase in the excitation power density results in an increase in the absolute QY for the upconversion emissions of LiYO_2_:Er^3+^,Yb^3+^ and, after exceeding about 1000 W cm^–2^, a saturation regime is observed reaching values increasing from 0.15 to 0.20% (Table 1). As expected, the emission QY values for the downshifting emission in the NIR spectral range, corresponding to the ^4^I_13/2_ → ^4^I_15/2_ transition, are independent of the excitation power. Notably, the impact of the Yb^3+^ concentration is much higher in the downshifting QY values (see Figures S9 and S10).

**Table 1:** Emission QY Values of the LiYO_2_:Er^**3**+^Yb^**3**+^ Samples upon 975 nm Excitation (1135 W cm^–**2**^) for Upconversion (Integration Range of 483–920 nm) and Downshifting (Integration Range of 1007–1650 nm) Emissions^a^

sample	upconversion QY (%)	downshifting QY (%)
LiYO_2_:Er^3+^,1%Yb^3+^	0.15 ± 0.02	36 ± 4
LiYO_2_:Er^3+^,2%Yb^3^	0.20 ± 0.02	36 ± 4
LiYO_2_:Er^3+^,5%Yb^3+^	0.18 ± 0.02	30 ± 3
LiYO_2_:Er^3+^,10%Yb^3+^	0.14 ± 0.01	17 ± 2

a The uncertainty on the QY values corresponds to 10% of the measured value, according to the setup manufacturer.

**Figure 4:**
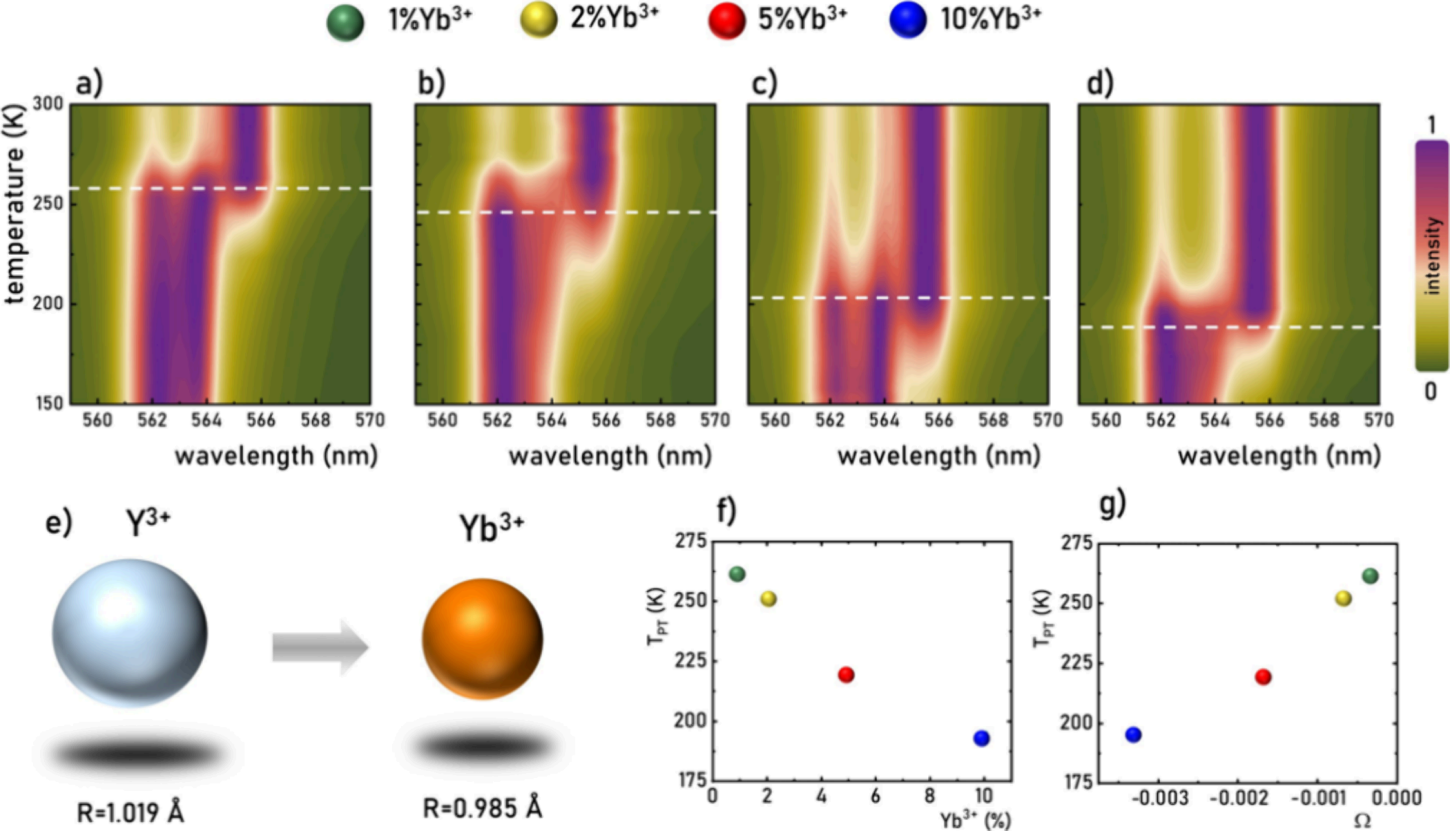
Thermal luminescence maps of LiYO_2_:Er^3+^,Yb^3+^ in 555–570 nm spectral range for 1%Yb^3+^ (a); 2%Yb^3+^ (b); 5%Yb^3+^ (c); and 10%Yb^3+^ (d) dopant concentration; the comparison of the ionic radii of Y^3+^ and Yb^3+^ ions responsible for the change in the phase transition temperature of LiYO_2_:Er^3+^,Yb^3+^ (e) the temperature of the phase transition (*T*_PT_) as a function of Yb^3+^ concentration (f) and Ω parameter (g).

As demonstrated, the analysis of the luminescence spectra of the LiYO_2_:Er^3+^,Yb^3+^ systems proves the effectivity of an optical-based technique in identifying the temperature-induced phase transition temperature. As the XRD data reveal the influence of Yb^3+^ ions concentration on the phase transition temperature, parallel investigations based on the luminescence response of the luminophore were conducted. In particular, temperature-dependent luminescence spectra of samples featuring varying concentrations of Yb^3+^ ions were analyzed. The resultant normalized luminescence maps, obtained over a narrow spectral range, reveal differences in the phase transition temperature for the sample with different amounts of sensitizer ions (Figure 4a–d). Despite the concentration of Yb^3+^ ions not influencing the spectral positions of the bands, the temperature of phase transition demonstrably decreases with the addition of the codopant. The observed effect arises from the difference in ionic radii between the host material ions Y^3+^ (*R*_0_ = 1.019 Å) and the dopant ion Yb^3+^ (*R* = 0.985 Å) (Figure 4e). The incremental introduction of dopant ions leads to a successive reduction in the LiYO_2_ unit cell, thereby lowering the phase transition temperature from 265 K, for LiYO_2_:Er^3+^, 1%Yb^3+^, to 180 K, for 10%Yb^3+^ (Figure 4f). The mismatch in ionic radii between host material and dopant ions can be quantified by the determination of the Ω parameter:^30^
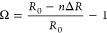
3where *R*_0_ represents the ionic radius of Y^3+^ ions, *n* is the codopant ion concentration, and Δ*R* is the difference between *R*_0_ and the ionic radii of the host material ions and codopant.

Given that the Yb^3+^ ion is smaller than the ionic radius of the Y^3+^ ion, Ω assumes negative values (Figure 4g). Notably, the phase transition temperature is shown to be a monotonic function of Ω. This aspect is particularly significant, as it indicates that, by determining the value of Ω, the phase transition temperature can be quantitatively predicted with high precision, facilitating the design of a thermometer with predefined temperature parameters.

### Luminescence Thermometry through Thermally Coupled Energy Levels

**Figure 5:**
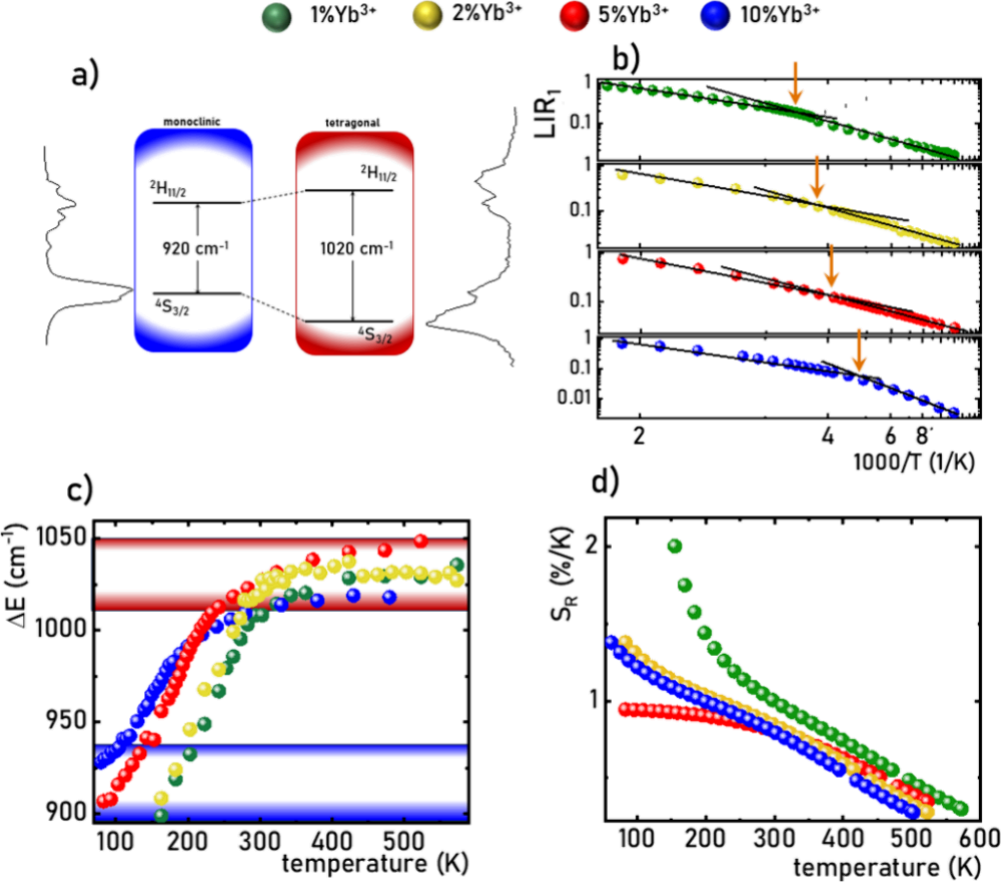
Energy gap Δ*E* between ^2^H_11/2_ and ^4^S_3/2_ states of Er^3+^ ions for monoclinic and tetragonal phases of LiYO_2_:Er^3+^,Yb^3+^ (a); thermal dependence of LIR_1_ for different concentrations of Yb^3+^ ions (b), the Δ*E* as a function of temperature for different concentration of Yb^3+^ ions (c), and the thermal dependence of *S*_R_ (d).

The Er^3+^ ions are widely utilized in luminescence thermometry due to the thermal coupling between the ^2^H_11/2_ and ^4^S_3/2_ levels, facilitating the development of a primary luminescent thermometer.^6,31−33^ In this context, the relative thermal sensitivity of the luminescent thermometer is directly proportional to the energy difference between the barycenter of the thermally coupled levels, Δ*E* (Figure 5a). The luminescence intensity ratio, LIR_1_, changes according to the Boltzmann distribution:
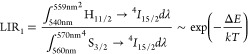
4where Δ*E* is the energy gap between ^2^H_11/2_ and ^4^S_3/2_ levels, *k* is the Boltzmann constant, and *T* is the absolute temperature. Hence, for this thermometer, a linear change in ln(*LIR*_1_) with 1/*T* is anticipated. However, for LiYO_2_:Er^3+^,Yb^3+^, two slopes are observed in the ln(LIR_1_) vs 1/*T* plot (Figure 5b). Significantly, the temperature at which the change in the slope of this straight line occurs depends on the concentration of Yb^3+^ ions, decreasing as the concentration increases, which is in good agreement with the previously determined phase transition temperatures. The observed change in the slope of the curve suggests a change in *ΔE*, which is independent of the temperature change for the conventional upconverting systems (without a structural phase transition). The analysis of upconversion luminescence spectra in LiYO_2_:Yb^3+^,Er^3+^ at 83 and 353 K reveals that the structural phase transition induces an increase in Δ*E* from 920 cm^–1^ for monoclinic to 1020 cm^–1^ for tetragonal phases (Figure 5c). The calculation of Δ*E* as a function of temperature (determined by the slope of the above-mentioned straight lines) shows a smooth change of *ΔE* over a temperature range of about 150 K. However, the temperature range in which the Δ*E* undergoes modification depends on the Yb^3+^ concentration. The observed difference in Δ*E* is related to two effects: (i) a change in the splitting of energy levels into Stark components and (ii) a change in the covalency of the Er^3+^-O^2–^ bond associated with the phase transition. The transition from a monoclinic to a tetragonal structure results in a reduction in the number of Stark levels and a change in their energy, influencing the energy of the ^2^H_11/2_ and ^4^S_3/2_ multiplets and, consequently, the Δ*E*. Unfortunately, the large number of Stark components and the small distance between Er^3+^ and Yb^3+^ ions in LiYO_2_:Er^3+^ complicate accurate analysis for the tetragonal phase. Additionally, Δ*E* is strongly dependent on the covalency of the Er^3+^–O^2–^ bond, with a shorter bond length leading to increased covalency, and then, a higher energy gap is expected. Studies on APO_4_ phosphates (A = La, Gd, Lu, Y) indicate that increased covalency primarily causes a decrease in the energy of both levels, with the ^4^S_3/2_ level responding more strongly to these changes than the ^2^H_11/2_ level.^34^ Similar behavior is observed for LiYO_2_:Er^3+^, suggesting that the change in the Er^3+^–O^2–^ distance is a major factor in the thermally induced structural transition affecting Δ*E*. To quantify the thermal changes of *LIR*_1_, the relative thermal sensitivity is calculated as
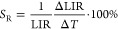
5

The typical values of the relative sensitivities can be found for the LiYO_2_:Er^3+^, Yb^3+^ of around 1.5% K^–1^ (Figure 5d), decreasing with temperature, in good agreement with the functional dependence of *S*_R_ with Δ*E/kT*^2^.

### Double-Band Luminescence Thermometry

**Figure 6:**
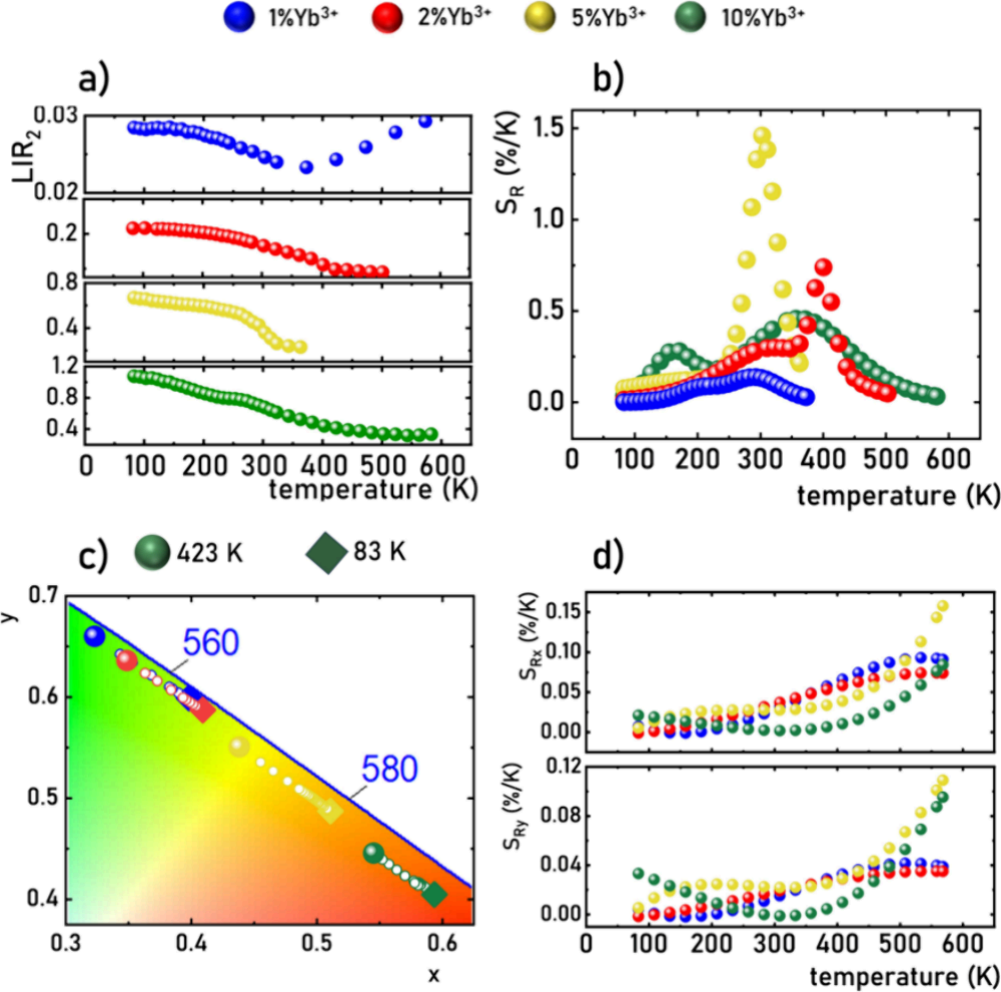
Thermal dependence of LIR_2_ for different concentrations of Yb^3+^ ions of LiYO_2_:Er^3+^,Yb^3+^ (operating temperature range marked in blue) (a) and corresponding *S*_R_ (b); thermally induced change in the chromatic coordinates (CIE 1931) for different concentrations of Yb^3+^ ions (c); and corresponding relative sensitivities based on the *x* and *y* chromatic coordinates (d).

Upon analysis of the luminescence spectra of LiYO_2_:Er^3+^,Yb^3+^ measured as a function of temperature, as depicted in Figure 2a (Figure S11), another spectral characteristic undergoing alterations with increasing temperature can be noticed: the green to the red band ratio. The temperature increase induces a more pronounced thermal quenching effect of the ^4^F_9/2_ → ^4^I_15/2_ band compared to the ^4^S_3/2_ → ^4^I_15/2_ one. This phenomenon can be explained by the growing probability of multiphonon depopulation processes of the ^4^F_9/2_ level, stemming from the reduced energy difference between this level and the lower lying ^4^I_9/2_ (2000 cm^–1^) in comparison to the ^4^S_3/2_ level and the lower lying ^4^F_9/2_ level (2800 cm^–1^). Consequently, the red-to-green emission intensity ratio (*LIR*_2_) defined as follows:
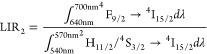
6

can be employed as another thermometric parameter. Irrespective of the concentration of Yb^3+^ ions, *LIR*_2_ exhibits a similar character of initial thermal changes, decreasing with increasing temperature (Figure 6a). An apparent inflection point on this tendency corresponds to the phase transition temperature, beyond which for the 1%Yb^3+^ sample a reversal in the observed dependence is noted. It is crucial to note that the initial intensity of the ^4^F_9/2_ → ^4^I_15/2_ band for 1%Yb^3+^ is relatively small compared to that of the ^4^S_3/2_ → ^4^I_15/2_ band, leading to a small initial value of LIR_2_. Consequently, values obtained above 400 K may be subject to high error due to the small signal-to-noise ratio. The most rapid changes in LIR_2_ values were observed for 5%Yb^3+^, resulting in a notably high relative sensitivity value of up to 1.5% K^–1^ at 305 K (comparable to the value obtained with LIR_1_) (Figure 6b). The observed variations in LIR_2_ values are reflected in the thermal modulation of the emitted light color. Irrespective of the concentration of Yb^3+^ ions, an elevation in temperature induces a shift in the color of the emitted light toward green. The most extensive color range of observed changes was recorded for 5%Yb^3+^. Such variations enable the development of a third thermometric parameter based on the change of the (*x,y*) color coordinates of 1931 by the Commission Internationale de l’Éclairage (CIE1931) (Figure 6c), and consequently, relative sensitivities-based CIE 1931 *x* and *y* parameters (*S*_Rx_ and *S*_Ry_, respectively) were determined analogously to eq 5 (Figure 6d, thermal dependence of S_Ax_ and S_Ay_ are presented in Figure S12).

### Single-Band Luminescence Thermometry

**Figure 7:**
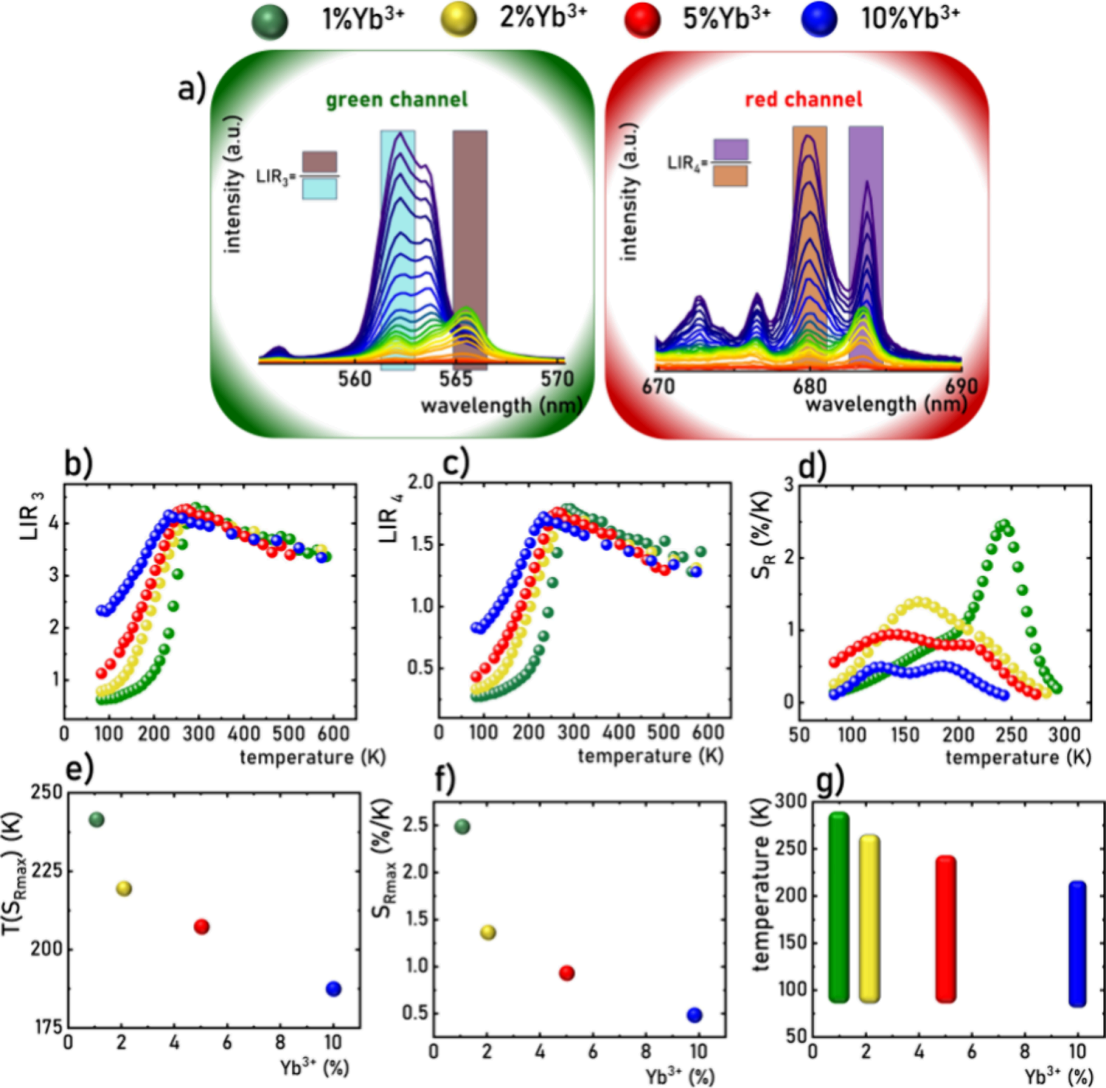
Emission spectra of LiYO_2_:Er^3+^, 1%Yb^3+^ measured as a function of temperature presented in the green and red parts of emission spectra (a); thermal dependence of LIR_3_ (b) and LIR_4_ (operating temperature range marked in blue) (c) for different concentrations of Yb^3+^ ions; and corresponding *S*_R_ (d); temperature *T*(*S*_Rmax_) at which the maximal (*S*_Rmax_) relative sensitivity was noted (e), the *S*_Rmax_ (f) and usable temperature range of luminescent thermometer, (g) as a function of Yb^3+^ concentration.

As previously demonstrated, the structural phase transition within LiYO_2_:Er^3+^,Yb^3+^ is discernible through a marked spectral shift in the luminescence spectrum of the Er^3+^ ions. Consequently, the ratio of intensities between lines associated with monoclinic and tetragonal phases can serve as a temperature parameter. The dispersive behavior of light scattering and absorption in diverse media is a critical factor that may impact ratiometric temperature measurements (one of the example of such applications is the in vivo temperature measurements in biological systems).^5,35^ In such cases, using the intensity ratio of two bands with significant spectral separation may introduce distortions in the temperature readings obtained through LIR measurements. Most reported cases employ the ratio of luminescence intensities between bands originating from different Stark components of the emitting level, which are thermalized with increasing temperature.^36,37^ However, the small energy differences between these Stark components typically result in sensitivities not exceeding 0.3% K^–1^. The utilization of a phase transition-based thermometer offers the potential to overcome the limitations associated with low sensitivity, facilitating the development of highly sensitive single-band ratiometric thermometers. Importantly, the use of Er^3+^ ions enables the observation of thermally induced spectral changes in both the green (^4^S_3/2_ → 4I_15/2_ transition) and red (^4^F_9/2_ → ^4^I_15/2_ transition) spectral ranges (Figure 7a). This allows for the development of two additional thermometric parameters, namely, LIR_3_ and LIR_4_, defined as follows:
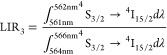
7
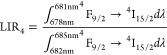
8

Since the thermal changes in LIR_3_ (Figure 7b) and LIR_4_ (Figure 7c) are induced by the same structural process, their thermal dynamics is similar. Both parameters show an increase with rising temperature until reaching a maximum value near the phase transition temperature followed by a slight decrease. The broad thermal range of these intensity ratio changes results from the spectral band overlap in both structural phases, with differences in the initial values attributed to distinct band shapes. Nevertheless, the relative thermal sensitivities in both cases are nearly identical.

A reliable luminescent thermometer must exhibit monotonic changes within its thermal operating range. Consequently, the reversal of monotonicity in LIRs above the phase transition temperature precludes their use at high temperatures.^38,39^ Relative thermal sensitivities were therefore determined within the temperature range below the temperature corresponding to the maximum LIR value (Figure 7d). As expected, the temperature at which the maximum sensitivity ratio (*S*_*R*_) value was achieved decreases monotonically with increasing dopant concentration (Figure 7e). Additionally, the *S*_*R*_ value decreases with increasing concentration from *S*_R_ = 2.5% K^–1^ for 1% Yb^3+^ to *S*_R_ = 0.5% K^–1^ for 10% Yb^3+^ (Figure 7f). The notably high sensitivity values obtained for the single-band ratiometric thermometer position LiYO_2_:Er^3+^,Yb^3+^ as a significant candidate for luminescence-based temperature sensing (Figure 7g). Moreover, the versatility of this material in both the red and green spectral ranges enhances its potential for diverse applications. It is essential to note, however, that an increase in the dopant concentration narrows the useful operating range of the thermometer. The reproducibility of the temperature readout is an important parameter. Therefore, for the luminescent thermometer of the highest relative sensitivity LiYO_2_:1%Yb^3+^,1%Er^3+^, the LIR values within the heating–cooling cycles were determined (Figure S13). As can be noticed, the values of LIR confirm its high applicative potential. However, the careful analysis of the thermal dependence of LIR in a whole temperature range indicates the small hysteresis loop, which is expected for phase transition and was already reported for other materials.^14−16,30^ Therefore, the developed thermometers should be used in applications where the monotonicity of the temperature change is known and does not change during the experiment.

## Conclusions

In summary, the present investigation delves into the impact of temperature variations and thermally induced structural phase transitions in LiYO_2_:Er^3+^,Yb^3+^ on their upconversion luminescence properties. Elevating temperatures prompt a reversible phase transition from the low-temperature monoclinic phase to the high-temperature tetragonal phase. This structural alteration induces a modification in the point symmetry of the Y^3+^ ion from C_2_ to D_2d_, subsequently influencing the spectroscopic attributes of the Er^3+^ ion. Consequently, as the temperature increases, discernible shifts in the spectral position of the bands become apparent. The intensity ratio of spectral lines associated with distinct polymorphs of LiYO_2_ facilitates the development of single-band ratiometric luminescent thermometers, operating within both green and red spectral ranges, exhibiting a sensitivity of *S*_R_ = 2.5% K^–1^ at 243 K. The elevation in the concentration of sensitizer ions (Yb^3+^), attributed to the disparity in ionic radii relative to the host material, serves to lower the phase transition temperature, thereby optimizing the temperature at which maximum relative sensitivity is achieved. Additionally, an abundance of Yb^3+^ ions narrows the effective temperature range of the thermometer and alters the emission color from green to red. The described phase transition, resulting from energy changes in the ^2^H_11/2_ and ^4^S_3/2_ levels, contributes to an augmented energy gap between the levels, thereby slightly enhancing the sensitivity of the classical ^4^S_3/2_/^2^H_11/2_ → ^4^I_15/2_ luminescent thermometer. Additionally, the more efficient thermal depopulation of the ^4^F_9/2_ level compared to the ^4^S_3/2_ one induces a shift in the emitted light color toward green, presenting opportunities for luminescence thermometry founded on luminescence thermochromism.

This research on the inaugural phase-transition-based upconverting luminescent thermometer underscores its significant applicative potential and constitutes a valuable contribution to ongoing investigations in this highly promising field.

## Supporting Information Available

Results of the Rietveld refinement of the LiYO_2_:Er^3+^,Yb^3+^ XRD patterns measured at different temperatures, thermal dependence of absolute sensitivities, and LIR within heating–cooling cycles (PDF)

## References

[ref1] Brites C. D. S.; Balabhadra S.; Carlos L. D. Lanthanide-Based Thermometers: At the Cutting-Edge of Luminescence Thermometry. Adv. Opt. Mater. 2019, 7 (5), 1801239 10.1002/adom.201801239.

[ref2] Brites C. D. S.; Millán A.; Carlos L. D; Lanthanides in Luminescent Thermometry; In Handbook on the Physics and Chemistry of Rare Earths; Jean-Claude B.; Vitalij K.; H P B T Eds.; Elsevier, 2016; Vol. 49, pp. 339–427. doi:10.1016/bs.hpcre.2016.03.005.

[ref3] Jaque D.; Vetrone F. Luminescence Nanothermometry. Nanoscale 2012, 4 (15), 4301–4326. 10.1039/c2nr30764b. 22751683

[ref4] Zhou J.; del Rosal B.; Jaque D.; Uchiyama S.; Jin D. Advances and Challenges for Fluorescence Nanothermometry. Nat. Methods 2020, 17 (10), 967–980. 10.1038/s41592-020-0957-y. 32989319

[ref5] Bednarkiewicz A.; Marciniak L.; Carlos L. D.; Jaque D. Standardizing Luminescence Nanothermometry for Biomedical Applications. Nanoscale 2020, 12 (27), 14405–14421. 10.1039/D0NR03568H. 32633305

[ref6] Martins J. C.; Brites C. D. S.; Neto A. N. C.; Ferreira R. A. S.; Carlos L. D.; An Overview of Luminescent Primary Thermometers BT—Luminescent Thermometry: Applications and Uses; Carvajal Martí J. J.; Pujol Baiges M. C., Eds.; Springer International Publishing: Cham, 2023, pp. 105–152. doi:10.1007/978-3-031-28516-5_3.

[ref7] Marciniak L.; Kniec K.; Elżbieciak-Piecka K.; Trejgis K.; Stefanska J.; Dramićanin M. Luminescence Thermometry with Transition Metal Ions. A Review. Coord. Chem. Rev. 2022, 469, 214671 10.1016/j.ccr.2022.214671.

[ref8] Dramićanin M. D. Trends in Luminescence Thermometry. J. Appl. Phys. 2020, 128 (4), 40902 10.1063/5.0014825.

[ref9] Dramićanin M. D. Sensing Temperature via Downshifting Emissions of Lanthanide-Doped Metal Oxides and Salts. A Review. Methods Appl. Fluoresc. 2016, 4 (4), 42001 10.1088/2050-6120/4/4/042001. 28192289

[ref10] Ximendes E.; Marin R.; Carlos L. D.; Jaque D. Less Is More: Dimensionality Reduction as a General Strategy for More Precise Luminescence Thermometry. Light Sci. Appl. 2022, 11 (1), 237 10.1038/s41377-022-00932-3. 35896538 PMC9329371

[ref11] Trejgis K.; Ledwa K.; Li L.; Marciniak L. Effect of the Nanoparticle Size on Thermometric Properties of a Single-Band Ratiometric Luminescent Thermometer in NaYF4:Nd3+. J. Mater. Chem. C 2022, 10 (8), 3006–3014. 10.1039/D1TC06069D.

[ref12] Li K.; Dai M.; Fu Z.; Wang Z.; Xu H.; Wang R. A Latest-Generation Fluoride with Excellent Structural Stiffness for Ultra-Efficient Photoluminescence and Specific Four-Peak Emission Temperature Sensing. Inorg. Chem. Front. 2023, 11 (1), 172–185. 10.1039/D3QI01902K.

[ref13] Xu H.; Jia M.; Wang Z.; Wei Y.; Fu Z. Enhancing the Upconversion Luminescence and Sensitivity of Nanothermometry through Advanced Design of Dumbbell-Shaped Structured Nanoparticles. ACS Appl. Mater. Interfaces 2021, 13 (51), 61506–61517. 10.1021/acsami.1c17900. 34910472

[ref14] Marciniak L.; Piotrowski W.; Szalkowski M.; Kinzhybalo V.; Drozd M.; Dramicanin M.; Bednarkiewicz A. Highly Sensitive Luminescence Nanothermometry and Thermal Imaging Facilitated by Phase Transition. Chem. Eng. J. 2022, 427, 131941 10.1016/j.cej.2021.131941.

[ref15] Marciniak L.; Piotrowski W. M.; Drozd M.; Kinzhybalo V.; Bednarkiewicz A.; Dramicanin M. Phase Transition-Driven Highly Sensitive, NIR–NIR Band-Shape Luminescent Thermometer Based on LiYO2:Nd3+. Adv. Opt. Mater. 2022, 10 (9), 2102856 10.1002/adom.202102856.

[ref16] Wang S.; Zhang J.; Ye Z.; Yu H.; Zhang H. Exploiting Novel Optical Thermometry near Room Temperature with a Combination of Phase-Change Host and Luminescent Pr3+ Ion. Chem. Eng. J. 2021, 414, 128884 10.1016/j.cej.2021.128884.

[ref17] Suo H.; Guo D.; Zhao P.; Zhang X.; Wang Y.; Zheng W.; Li P.; Yin T.; Guan L.; Wang Z.; Wang F. Ultrasensitive Colorimetric Luminescence Thermometry by Progressive Phase Transition. Adv. Sci. 2024, 11 (7), 2305241 10.1002/advs.202305241. PMC10870082 38084003

[ref18] Solé J. G.; Bausá L. E.; Jaque D. Applications: Rare Earth and Transition Metal Ions, and Color Centers. An Introduction to the Optical Spectroscopy of Inorganic Solids. 2005, 199–234. 10.1002/0470016043.ch6.

[ref19] Muhammad N.; Khan A.; Haidar Khan S.; Sajjaj Siraj M.; Shah S. S. A.; Murtaza G. Engel-Vosko GGA Calculations of the Structural, Electronic and Optical Properties of LiYO2. Phys. B Condens. Matter 2017, 521, 62–68. 10.1016/j.physb.2017.06.055.

[ref20] Antonov V. A.; Arsenev P. A.; Artykov Z. A.; Petrova D. S. Spectroscopic Properties of Dy3+ Ions in LiYO2 Single Crystals. J. Appl. Spectrosc. 1979, 31 (6), 1581–1584. 10.1007/BF01100282.

[ref21] Faucher M. D.; Sciau P.; Kiat J. M.; Alves M. G.; Bouree F. Refinement of the Monoclinic and Tetragonal Structures of Eu3+-Doped LiYO2by Neutron Diffraction at 77 and 383 K Differential Scanning Calorimetry, and Crystal Field Analysis. J. Solid State Chem. 1998, 137 (2), 242–248. 10.1006/jssc.1997.7713.

[ref22] Moune O. K.; Dexpert-Ghys J.; Piriou B.; Alves M. G.; Faucher M. D. Electronic Structure of Pr3+ and Tm3+ Doped LiYO2. J. Alloys Compd. 1998, 275–277, 258–263. 10.1016/s0925-8388(98)00316-8.

[ref23] Faucher M. D.; Moune O. K.; Alves M. G.; Piriou B.; Sciau P.; Pham-Thi M. Optical and Crystallographic Study of Eu3+ and Tb3+ Doped Liyo2: Phase Transition, Luminescence Efficiency and Crystal Field Calculation. J. Solid State Chem. 1996, 121 (2), 457–466. 10.1006/jssc.1996.0063.

[ref24] Shannon R. D. Revised Effective Ionic Radii and Systematic Studies of Interatomic Distances in Halides and Chalcogenides. Acta Crystallogr., Sect. A 1976, 32 (5), 751–767. 10.1107/S0567739476001551.

[ref25] Chen G.; Qiu H.; Prasad P. N.; Chen X. Upconversion Nanoparticles: Design, Nanochemistry, and Applications in Theranostics. Chem. Rev. 2014, 114 (10), 5161–5214. 10.1021/cr400425h. 24605868 PMC4039352

[ref26] Gnach A.; Bednarkiewicz A. Lanthanide-Doped up-Converting Nanoparticles: Merits and Challenges. Nano Today 2012, 7 (6), 532–563. 10.1016/j.nantod.2012.10.006.

[ref27] Zhou J.; Liu Q.; Feng W.; Sun Y.; Li F. Upconversion Luminescent Materials: Advances and Applications. Chem. Rev. 2015, 115 (1), 395–465. 10.1021/cr400478f. 25492128

[ref28] Wolfbeis O. S. An Overview of Nanoparticles Commonly Used in Fluorescent Bioimaging. Chem. Soc. Rev. 2015, 44 (14), 4743–4768. 10.1039/C4CS00392F. 25620543

[ref29] Zhou B.; Shi B.; Jin D.; Liu X. Controlling Upconversion Nanocrystals for Emerging Applications. Nat. Nanotechnol. 2015, 10 (11), 924–936. 10.1038/nnano.2015.251. 26530022

[ref30] Marciniak L.; Piotrowski W. M.; Szymczak M.; Drozd M.; Kinzhybalo V.; Back M. Customizing Thermometry: Optimizing the Operating Temperature Range of Phase Transition-Based Ratiometric Luminescence Thermometers. Chem. Eng. J. 2024, 487, 150363 10.1016/j.cej.2024.150363.

[ref31] Balabhadra S.; Debasu M. L.; Brites C. D. S.; Ferreira R. A. S.; Carlos L. D. Upconverting Nanoparticles Working As Primary Thermometers in Different Media. J. Phys. Chem. C 2017, 121 (25), 13962–13968. 10.1021/acs.jpcc.7b04827.

[ref32] Maturi F. E.; Gaddam A.; Brites C. D. S.; Souza J. M. M.; Eckert H.; Ribeiro S. J. L.; Carlos L. D.; Manzani D. Extending the Palette of Luminescent Primary Thermometers: Yb3+/Pr3+ Co-Doped Fluoride Phosphate Glasses. Chem. Mater. 2023, 35 (17), 7229–7238. 10.1021/acs.chemmater.3c01508. 37719033 PMC10500981

[ref33] Corredoira-Vázquez J.; González-Barreira C.; García-Deibe A. M.; Sanmartín-Matalobos J.; Hernández-Rodríguez M. A.; Brites C. D. S.; Carlos L. D.; Fondo M. Harnessing Ligand Design to Develop Primary and Self-Calibrated Luminescent Thermometers with Field-Induced Single Ion Magnet Behaviour in Dy3+ Complexes. Inorg. Chem. Front. 2024, 11 (4), 1087–1098. 10.1039/D3QI02374E.

[ref34] Maciejewska K.; Bednarkiewicz A.; Meijerink A.; Marciniak L. Correlation between the Covalency and the Thermometric Properties of Yb3+/Er3+Codoped Nanocrystalline Orthophosphates. J. Phys. Chem. C 2021, 125 (4), 2659–2665. 10.1021/acs.jpcc.0c09532. PMC7876742 33584938

[ref35] Labrador-Páez L.; Pedroni M.; Speghini A.; García-Solé J.; Haro-González P.; Jaque D. Reliability of Rare-Earth-Doped Infrared Luminescent Nanothermometers. Nanoscale 2018, 10 (47), 22319–22328. 10.1039/C8NR07566B. 30468230

[ref36] Hazra C.; Skripka A.; Ribeiro S. J. L.; Vetrone F. Erbium Single-Band Nanothermometry in the Third Biological Imaging Window: Potential and Limitations. Adv. Opt. Mater. 2020, 8 (23), 2001178 10.1002/adom.202001178.

[ref37] Wawrzynczyk D.; Bednarkiewicz A.; Nyk M.; Strek W.; Samoc M. Neodymium(Iii) Doped Fluoride Nanoparticles as Non-Contact Optical Temperature Sensors. Nanoscale 2012, 4 (22), 6959–6961. 10.1039/c2nr32203j. 23072978

[ref38] Woźny P.; Soler-Carracedo K.; Stopikowska N.; Martín I. R.; Runowski M. Structure-Dependent Luminescence of Eu3+-Doped Strontium Vanadates Synthesized with Different V: Sr Ratios – Application in WLEDs and Ultra-Sensitive Optical Thermometry. J. Mater. Chem. C 2023, 11 (14), 4792–4807. 10.1039/D2TC05341A.

[ref39] Qiu X.; Zheng T.; Runowski M.; Woźny P.; Martín I. R.; Soler-Carracedo K.; Piñero C. E.; Lebedkin S.; Fuhr O.; Bräse S. Constructing [2.2]Paracyclophane-Based Ultrasensitive Optical Fluorescent-Phosphorescent Thermometer with Cucurbit[8]Uril Supramolecular Assembly. Adv. Funct. Mater. 2024, 2313517 10.1002/adfm.202313517.

