# Supplementary material for: The Butterfly Effect: Multifaceted Consequences of Sensitizer Concentration Change in Phase Transition-based Luminescent Thermometer of LiYO2:Er3+,Yb3+
Source: ACS Appl Mater Interfaces. 2024 May 13;16(20):26439–49. doi: 10.1021/acsami.4c03856 (PMC11129115; doi:10.1021/acsami.4c03856)
Supplement: Supplementary file 1 — am4c03856_si_001.pdf [file am4c03856_si_001.pdf]

Supporting Information

The butterfly effect: multifaceted consequences of sensitizer concentration change in phase transition based luminescence thermometer of $LiYO_2:Er^{3+},Yb^{3+}$

L. Marciniak[1*], W. Piotrowski[1], M. Szymczak[1], C. D. S. Brites[2], V. Kinzhybalo[1], Hao Suo[3], L.D. Carlos[2], Feng Wang[3],

[1]Institute of Low Temperature and Structure Research, Polish Academy of Sciences, Okolna 2, 50-422 Wroclaw, Poland

2 Physics Department and CICECO—Aveiro Institute of Materials, University of Aveiro, Aveiro, Portugal3 Department of Materials Science and Engineering, City University of Hong Kong, Kowloon, Hong Kong SAR, 999077 China

* corresponding author*l.marciniak@intibs.pl*

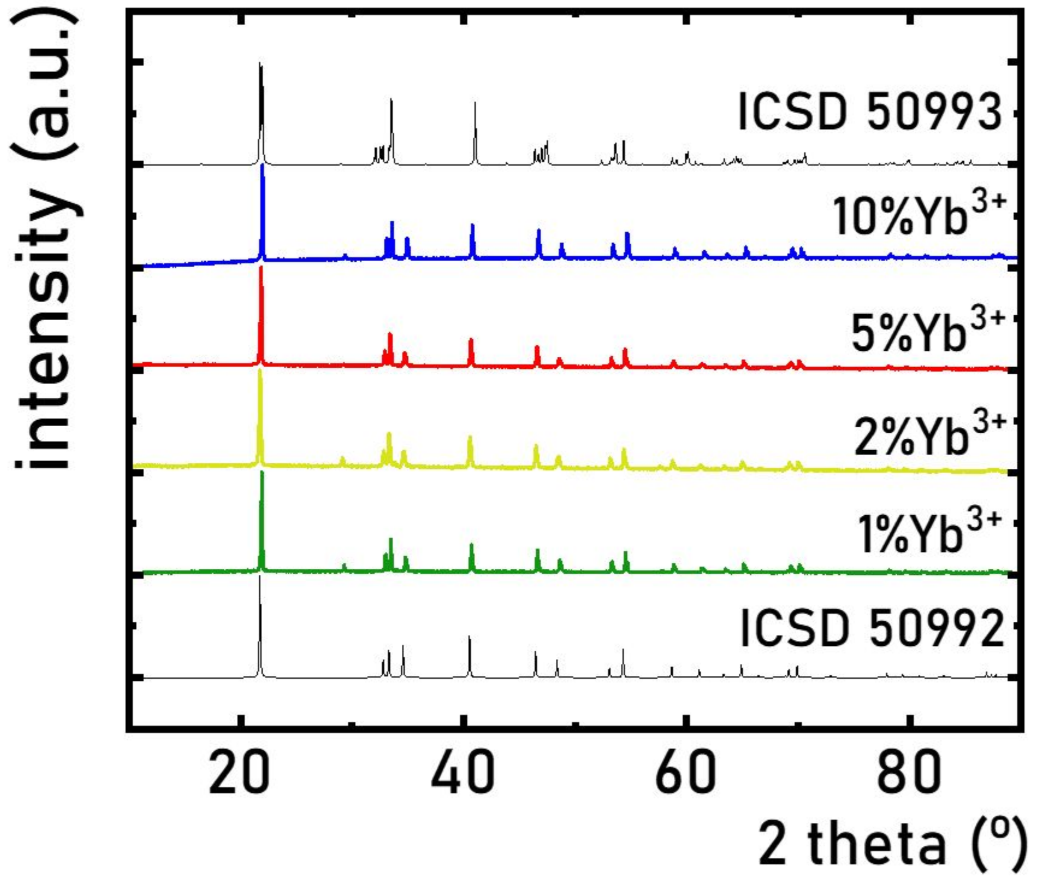

Figure S1. Comparison of the room temperature XRD for $LiYO_2:Yb^{3+},Er^{3+}$ with different concentration of Yb^{3+} ions

The results of the Rietveld refinement of the XRD patterns of $LiYO_2:Er^{3+},Yb^{3+}$ measured at different temperatures:

The data for the $LiYO_2$:2%Yb^{3+}, 1%Er^{3+} at 100 K:

Global Parameters

Number of used phases:	3	V standard:	0.000000
Number of variables:	21	W standard:	0.010000
Number of constraints:	2	U Left:	0.000000
Zero shift/ °2Theta:	0.000000	V Left:	0.000000
Specimen displacement/ mm :	0.284(2)	W Left:	0.010000
Profile function:	Pseudo Voigt	U Right:	0.000000
Background:	Polynomial	V Right:	0.000000
R (expected)/ %:	0.54798	W Right:	0.010000
R (profile)/ %:	3.73507	Asymmetry Type:	No Asymmetry Function
R (weighted profile)/ %:	5.48660		
GOF:	100.24980	Asymmetry 1:	0.000000
d-statistic:	0.14603	Asymmetry 2:	0.000000
U standard:	0.000000	Shape Type:	Shape Individual

Shape 1 Left:	0.600000	Crystal Shape Factor K:	1.0000
Shape 2 Left:	0.000000	Instrumental FWHM Curve Type:	Caglioti function
Shape 3 Left:	0.000000	Instr. Gauss Curve Coefficient A:	0.0045(5)
Shape 1 Right:	0.600000	Instr. Gauss Curve Coefficient B:	-0.0032(9)
Shape 2 Right:	0.000000	Instr. Gauss Curve Coefficient C:	0.0046(3)
Shape 3 Right:	0.000000	Instr. Lorentz Curve Coefficient A:	0.0062(7)
K a1/a2 intensity ratio:	0.500000	Instr. Lorentz Curve Coefficient B:	-0.004(1)
K alpha/beta intensity ratio:	0.000000	Instr. Lorentz Curve Coefficient C:	0.0064(5)

Relevant parameters of $LiYO_2$-LT, 50992-ICSD

Structure and profile data:		Extinction:	0.000000
Formula sum: $Y_{3.88}Li_{4.00}O_{8.00}Yb_{0.08}Er_{0.04}$		Flat Plate Absorption Correction:	0.000000
Formula mass/ g/mol:	521.2477	Porosity:	0.000000
Density (calculated)/ g/cm^3	4.2062	Roughness:	0.000000
F(000):	235.6400	Fitting mode:	Structure Fit
Weight fraction/ %:	95.6(3)	U Left:	0.17(3)
Space group (No.):	P 1 21/c 1 (14)	V Left:	-0.02(2)
Lattice parameters:		W Left:	0.028(3)
a/ A:	6.1230(2)	Preferred orientation direction/ hkl:	0.00 0.00 1.00
b/ A:	6.1787(3)	Preferred orientation parameter:	1.000000
c/ A:	6.2089(2)	Asymmetry parameter 1:	0.25(2)
alpha/ °:	90	Asymmetry parameter 2:	0.000000
beta/ °:	118.845(2)	Peak shape:	
gamma/ °:	90	parameter 1 Left:	0.38(1)
V/ 10^6 pm^3	205.75030	parameter 2 Left:	0.000000
Overall displacement parameter:	0.000000	parameter 3 Left:	0.000000
		R (Bragg)/ %:	3.93880

Occupancy, atomic fract. coordinates and Biso for $LiYO_2$-LT, 50992-ICSD

Atom	Wyck.	s.o.f.	x	y	z	B/ 10^4 pm^2
Y1	4e	0.970000	0.266000	0.131200	0.020700	0.210000
Li1	4e	1.000000	0.791000	0.346000	0.068000	0.600000
O1	4e	1.000000	0.045900	0.107000	0.237800	0.280000
O2	4e	1.000000	0.512100	0.328800	0.343900	0.290000
Yb1	4e	0.020000	0.266000	0.131200	0.020700	0.210000
Er1	4e	0.010000	0.266000	0.131200	0.020700	0.210000

Relevant parameters of $LiYO_2$-HT, 50993-ICSD

Structure and profile data:		Extinction:	0.000000
Formula sum: $Y_{3.88}Li_{4.00}O_{8.00}Yb_{0.08}Er_{0.04}$		Flat Plate Absorption Correction:	0.000000
Formula mass/ g/mol:	521.2477	Porosity:	0.000000
Density (calculated)/ g/cm^3	4.2712	Roughness:	0.000000
F(000):	235.6400	Fitting mode:	Structure Fit
Weight fraction/ %:	0.000000	U Left:	0.068937
Space group (No.):	I 41/a m d (141)	V Left:	-0.052995
Lattice parameters:		W Left:	0.027085
a/ A: 4(437541797888)		Preferred orientation direction/ hkl:	0.00 0.00 1.00
b/ A: 4(437541797888)		Preferred orientation parameter:	1.000000
c/ A: 10(5451897896960)		Asymmetry parameter 1:	0.170069
alpha/ °:	90	Asymmetry parameter 2:	0.000000
beta/ °:	90	Peak shape:	
gamma/ °:	90	parameter 1 Left:	0.626711
V/ 10^6 pm^3	202.62080	parameter 2 Left:	0.000000
Overall displacement parameter:	0.000000	parameter 3 Left:	0.000000
		R (Bragg)/ %:	999.00000

Occupancy, atomic fract. coordinates and Biso for $LiYO_2$-HT, 50993-ICSD

Atom	Wyck.	s.o.f.	x	y	z	B/ 10^4 pm^2
Y1	4b	0.970000	0.000000	0.250000	0.375000	0.000000
Li1	4b	0.000000	0.000000	0.250000	0.375000	0.000000
Li2	4a	1.000000	0.000000	0.750000	0.125000	0.000000
O1	8e	1.000000	0.000000	0.250000	0.152000	0.000000
Yb1	4b	0.020000	0.000000	0.250000	0.375000	0.000000
Er1	4b	0.010000	0.000000	0.250000	0.375000	0.000000

Figure S2: Rietveld refinement of X-ray diffractograms of the $LiYO_2$:2%Yb^{3+}, 1%Er^{3+} at 120 K.

The data for the $LiYO_2$:2%Yb^{3+}, 1%Er^{3+} at 120 K:

Global Parameters

Number of used phases:	3	Asymmetry Type:	No Asymmetry Function
Number of variables:	21		
Number of constraints:	2	Asymmetry 1:	0.000000
Zero shift/ °2Theta:	0.000000	Asymmetry 2:	0.000000
Specimen displacement/ mm :	0.279(2)	Shape Type:	Shape Individual
Profile function:	Pseudo Voigt	Shape 1 Left:	0.600000
Background:	Polynomial	Shape 2 Left:	0.000000
R (expected)/ %:	0.54608	Shape 3 Left:	0.000000
R (profile)/ %:	3.80800	Shape 1 Right:	0.600000
R (weighted profile)/ %:	5.62373	Shape 2 Right:	0.000000
GOF:	106.05760	Shape 3 Right:	0.000000
d-statistic:	0.13443	K a1/a2 intensity ratio:	0.500000
U standard:	0.000000	K alpha/beta intensity ratio:	0.000000
V standard:	0.000000	Crystal Shape Factor K:	1.0000
W standard:	0.010000	Instrumental FWHM Curve Type:	Caglioti function
U Left:	0.000000	Instr. Gauss Curve Coefficient A:	0.0045(5)
V Left:	0.000000	Instr. Gauss Curve Coefficient B:	-0.0032(9)
W Left:	0.010000	Instr. Gauss Curve Coefficient C:	0.0046(3)
U Right:	0.000000	Instr. Lorentz Curve Coefficient A:	0.0062(7)
V Right:	0.000000	Instr. Lorentz Curve Coefficient B:	-0.004(1)
W Right:	0.010000	Instr. Lorentz Curve Coefficient C:	0.0064(5)

Relevant parameters of $LiYO_2$-LT, 50992-ICSD

Structure and profile data:		Extinction:	0.000000
Formula sum: $Y_{3.88}Li_{4.00}O_{8.00}Yb_{0.08}Er_{0.04}$		Flat Plate Absorption Correction:	0.000000
Formula mass/ g/mol:	521.2477	Porosity:	0.000000
Density (calculated)/ g/cm^3	4.2071	Roughness:	0.000000
F(000):	235.6400	Fitting mode:	Structure Fit
Weight fraction/ %:	95.9(3)	U Left:	0.17(3)
Space group (No.):	P 1 21/c 1 (14)	V Left:	-0.02(2)
Lattice parameters:		W Left:	0.029(3)
a/ A:	6.1208(2)	Preferred orientation direction/ hkl:	0.00 0.00 1.00
b/ A:	6.1805(3)	Preferred orientation parameter:	1.000000
c/ A:	6.2063(3)	Asymmetry parameter 1:	0.26(2)
alpha/ °:	90	Asymmetry parameter 2:	0.000000
beta/ °:	118.818(2)	Peak shape:	
gamma/ °:	90	parameter 1 Left:	0.35(1)
V/ 10^6 pm^3	205.70570	parameter 2 Left:	0.000000
Overall displacement parameter:	0.000000	parameter 3 Left:	0.000000
		R (Bragg)/ %:	4.18270

Occupancy, atomic fract. coordinates and Biso for $LiYO_2$-LT, 50992-ICSD

Atom	Wyck.	s.o.f.	x	y	z	B/ 10^4 pm^2
Y1	4e	0.970000	0.266000	0.131200	0.020700	0.210000
Li1	4e	1.000000	0.791000	0.346000	0.068000	0.600000
O1	4e	1.000000	0.045900	0.107000	0.237800	0.280000
O2	4e	1.000000	0.512100	0.328800	0.343900	0.290000
Yb1	4e	0.020000	0.266000	0.131200	0.020700	0.210000
Er1	4e	0.010000	0.266000	0.131200	0.020700	0.210000

Relevant parameters of LiYO2-HT, 50993-ICSD

Structure and profile data:		Extinction:	0.000000
Formula sum: $Y_{3.88}Li_{4.00}O_{8.00}Yb_{0.08}Er_{0.04}$		Flat Plate Absorption Correction:	0.000000
Formula mass/ g/mol:	521.2477	Porosity:	0.000000
Density (calculated)/ g/cm^3	4.2712	Roughness:	0.000000
F(000):	235.6400	Fitting mode:	Structure Fit
Weight fraction/ %:	0.000000	U Left:	0.068937
Space group (No.):	I 41/a m d (141)	V Left:	-0.052995
Lattice parameters:		W Left:	0.027085
a/ A:	4(450037678080)	Preferred orientation direction/ hkl:	0.00 0.00 1.00
b/ A:	4(450037678080)	Preferred orientation parameter:	1.000000
c/ A:	10(5607599374336)	Asymmetry parameter 1:	0.170069
alpha/ °:	90	Asymmetry parameter 2:	0.000000
beta/ °:	90	Peak shape:	
gamma/ °:	90	parameter 1 Left:	0.626711
V/ 10^6 pm^3	202.62080	parameter 2 Left:	0.000000
Overall displacement parameter:	0.000000	parameter 3 Left:	0.000000
		R (Bragg)/ %:	999.00000

Occupancy, atomic fract. coordinates and Biso for $LiYO_2$-HT, 50993-ICSD

Atom	Wyck.	s.o.f.	x	y	z	B/ 10^4 pm^2
Y1	4b	0.970000	0.000000	0.250000	0.375000	0.000000
Li1	4b	0.000000	0.000000	0.250000	0.375000	0.000000
Li2	4a	1.000000	0.000000	0.750000	0.125000	0.000000
O1	8e	1.000000	0.000000	0.250000	0.152000	0.000000
Yb1	4b	0.020000	0.000000	0.250000	0.375000	0.000000
Er1	4b	0.010000	0.000000	0.250000	0.375000	0.000000

The data for the $LiYO_2$:2%Yb^{3+}, 1%Er^{3+} at 140 K:

Global Parameters

Number of used phases:	3	Asymmetry Type:	No Asymmetry Function
Number of variables:	21		
Number of constraints:	2	Asymmetry 1:	0.000000
Zero shift/ °2Theta:	0.000000	Asymmetry 2:	0.000000
Specimen displacement/ mm :	0.273(2)	Shape Type:	Shape Individual
Profile function:	Pseudo Voigt	Shape 1 Left:	0.600000
Background:	Polynomial	Shape 2 Left:	0.000000
R (expected)/ %:	0.54493	Shape 3 Left:	0.000000
R (profile)/ %:	3.89235	Shape 1 Right:	0.600000
R (weighted profile)/ %:	5.77806	Shape 2 Right:	0.000000
GOF:	112.43180	Shape 3 Right:	0.000000
d-statistic:	0.12675	K a1/a2 intensity ratio:	0.500000
U standard:	0.000000	K alpha/beta intensity ratio:	0.000000
V standard:	0.000000	Crystal Shape Factor K:	1.0000
W standard:	0.010000	Instrumental FWHM Curve Type:	Caglioti function
U Left:	0.000000	Instr. Gauss Curve Coefficient A:	0.0045(5)
V Left:	0.000000	Instr. Gauss Curve Coefficient B:	-0.0032(9)
W Left:	0.010000	Instr. Gauss Curve Coefficient C:	0.0046(3)
U Right:	0.000000	Instr. Lorentz Curve Coefficient A:	0.0062(7)
V Right:	0.000000	Instr. Lorentz Curve Coefficient B:	-0.004(1)
W Right:	0.010000	Instr. Lorentz Curve Coefficient C:	0.0064(5)

Relevant parameters of $LiYO_2$-LT, 50992-ICSD

Structure and profile data:		Extinction:	0.000000
Formula sum: $Y_{3.88}Li_{4.00}O_{8.00}Yb_{0.08}Er_{0.04}$		Flat Plate Absorption Correction:	0.000000
Formula mass/ g/mol:	521.2477	Porosity:	0.000000
Density (calculated)/ g/cm^3	4.2076	Roughness:	0.000000
F(000):	235.6400	Fitting mode:	Structure Fit
Weight fraction/ %:	95.8(3)	U Left:	0.17(3)
Space group (No.):	P 1 21/c 1 (14)	V Left:	-0.01(2)
Lattice parameters:		W Left:	0.029(3)
a/ A:	6.1190(2)	Preferred orientation direction/ hkl:	0.00 0.00 1.00
b/ A:	6.1829(3)	Preferred orientation parameter:	1.000000
c/ A:	6.2032(3)	Asymmetry parameter 1:	0.33(2)
alpha/ °:	90	Asymmetry parameter 2:	0.000000
beta/ °:	118.787(2)	Peak shape:	
gamma/ °:	90	parameter 1 Left:	0.34(1)
V/ 10^6 pm^3	205.68200	parameter 2 Left:	0.000000
Overall displacement parameter:	0.000000	parameter 3 Left:	0.000000
		R (Bragg)/ %:	4.67387

Occupancy, atomic fract. coordinates and Biso for $LiYO_2$-LT, 50992-ICSD

Atom	Wyck.	s.o.f.	x	y	z	B/ 10^4 pm^2
Y1	4e	0.970000	0.266000	0.131200	0.020700	0.210000
Li1	4e	1.000000	0.791000	0.346000	0.068000	0.600000
O1	4e	1.000000	0.045900	0.107000	0.237800	0.280000
O2	4e	1.000000	0.512100	0.328800	0.343900	0.290000
Yb1	4e	0.020000	0.266000	0.131200	0.020700	0.210000
Er1	4e	0.010000	0.266000	0.131200	0.020700	0.210000

Relevant parameters of $LiYO_2$-HT, 50993-ICSD

Structure and profile data:		Space group (No.):	I 41/a m d (141)
Formula sum: $Y_{3.88}Li_{4.00}O_{8.00}Yb_{0.08}Er_{0.04}$		Lattice parameters:	
Formula mass/ g/mol:	521.2477	a/ A: 4(463364194304)	
Density (calculated)/ g/cm^3	4.2712	b/ A: 4(463364194304)	
F(000):	235.6400	c/ A: 10(5773651345408)	
Weight fraction/ %:	0.000000	alpha/ °:	90
		beta/ °:	90

gamma/ °:	90	W Left:	0.027085
V/ 10^6 pm^3	202.62080	Preferred orientation direction/ hkl:	0.00 0.00 1.00
Overall displacement parameter:	0.000000	Preferred orientation parameter:	1.000000
Extinction:	0.000000	Asymmetry parameter 1:	0.170069
Flat Plate Absorption Correction:	0.000000	Asymmetry parameter 2:	0.000000
Porosity:	0.000000	Peak shape:	
Roughness:	0.000000	parameter 1 Left:	0.626711
Fitting mode:	Structure Fit	parameter 2 Left:	0.000000
U Left:	0.068937	parameter 3 Left:	0.000000
V Left:	-0.052995	R (Bragg)/ %:	999.00000

Occupancy, atomic fract. coordinates and Biso for $LiYO_2$-HT, 50993-ICSD

Atom	Wyck.	s.o.f.	x	y	z	B/ 10^4 pm^2
Y1	4b	0.970000	0.000000	0.250000	0.375000	0.000000
Li1	4b	0.000000	0.000000	0.250000	0.375000	0.000000
Li2	4a	1.000000	0.000000	0.750000	0.125000	0.000000
O1	8e	1.000000	0.000000	0.250000	0.152000	0.000000
Yb1	4b	0.020000	0.000000	0.250000	0.375000	0.000000
Er1	4b	0.010000	0.000000	0.250000	0.375000	0.000000

The data for the $LiYO_2$:2%Yb^{3+}, 1%Er^{3+} at 160 K:

Global Parameters

Number of used phases:	3	Asymmetry Type:	No Asymmetry Function
Number of variables:	20		
Number of constraints:	1	Asymmetry 1:	0.000000
Zero shift/ °2Theta:	0.000000	Asymmetry 2:	0.000000
Specimen displacement/ mm :	0.262(2)	Shape Type:	Shape Individual
Profile function:	Pseudo Voigt	Shape 1 Left:	0.600000
Background:	Polynomial	Shape 2 Left:	0.000000
R (expected)/ %:	0.54574	Shape 3 Left:	0.000000
R (profile)/ %:	3.80051	Shape 1 Right:	0.600000
R (weighted profile)/ %:	5.54116	Shape 2 Right:	0.000000
GOF:	103.09220	Shape 3 Right:	0.000000
d-statistic:	0.14770	K a1/a2 intensity ratio:	0.500000
U standard:	0.000000	K alpha/beta intensity ratio:	0.000000
V standard:	0.000000	Crystal Shape Factor K:	1.0000
W standard:	0.010000	Instrumental FWHM Curve Type:	Caglioti function
U Left:	0.000000	Instr. Gauss Curve Coefficient A:	0.0045(5)
V Left:	0.000000	Instr. Gauss Curve Coefficient B:	-0.0032(9)
W Left:	0.010000	Instr. Gauss Curve Coefficient C:	0.0046(3)
U Right:	0.000000	Instr. Lorentz Curve Coefficient A:	0.0062(7)
V Right:	0.000000	Instr. Lorentz Curve Coefficient B:	-0.004(1)
W Right:	0.010000	Instr. Lorentz Curve Coefficient C:	0.0064(5)

Relevant parameters of $LiYO_2$-LT, 50992-ICSD

Structure and profile data:		Extinction:	0.000000
Formula sum: $Y_{3.88}Li_{4.00}O_{8.00}Yb_{0.08}Er_{0.04}$		Flat Plate Absorption Correction:	0.000000
Formula mass/ g/mol:	521.2477	Porosity:	0.000000
Density (calculated)/ g/cm^3	4.2077	Roughness:	0.000000
F(000):	235.6400	Fitting mode:	Structure Fit
Weight fraction/ %:	90.1(3)	U Left:	-0.10(2)
Space group (No.):	P 1 21/c 1 (14)	V Left:	0.15(1)
Lattice parameters:		W Left:	0.003(2)
a/ A:	6.1167(2)	Preferred orientation direction/ hkl:	0.00 0.00 1.00
b/ A:	6.1847(3)	Preferred orientation parameter:	1.000000
c/ A:	6.2011(2)	Asymmetry parameter 1:	0.32(2)
alpha/ °:	90	Asymmetry parameter 2:	0.000000
beta/ °:	118.744(2)	Peak shape:	
gamma/ °:	90	parameter 1 Left:	0.40(1)
V/ 10^6 pm^3	205.67830	parameter 2 Left:	0.000000
Overall displacement parameter:	0.000000	parameter 3 Left:	0.000000
		R (Bragg)/ %:	4.32160

Occupancy, atomic fract. coordinates and Biso for $LiYO_2$-LT, 50992-ICSD

Atom	Wyck.	s.o.f.	x	y	z	B/ 10^4 pm^2
Y1	4e	0.970000	0.266000	0.131200	0.020700	0.210000
Li1	4e	1.000000	0.791000	0.346000	0.068000	0.600000
O1	4e	1.000000	0.045900	0.107000	0.237800	0.280000
O2	4e	1.000000	0.512100	0.328800	0.343900	0.290000

Yb1	4e	0.020000	0.266000	0.131200	0.020700	0.210000
Er1	4e	0.010000	0.266000	0.131200	0.020700	0.210000

Relevant parameters of $LiYO_2$-HT, 50993-ICSD

Structure and profile data:	
Formula sum: $Y_{3.88}Li_{4.00}O_{8.00}Yb_{0.08}Er_{0.04}$	
Formula mass/ g/mol:	521.2477
Density (calculated)/ g/cm^3	4.2816
F(000):	235.6400
Weight fraction/ %:	5.6(2)
Space group (No.):	I 41/a m d (141)
Lattice parameters:	
a/ A:	4.429400
b/ A:	4.429400
c/ A:	10.302300
alpha/ °:	90
beta/ °:	90
gamma/ °:	90
V/ 10^6 pm^3	202.12680
Overall displacement parameter:	0.000000
Extinction:	0.000000
Flat Plate Absorption Correction:	0.000000
Porosity:	0.000000
Roughness:	0.000000
Fitting mode:	Structure Fit
U Left:	0.068937
V Left:	-0.052995
W Left:	0.027085
Preferred orientation direction/ hkl:	0.00 0.00 1.00
Preferred orientation parameter:	1.000000
Asymmetry parameter 1:	0.170069
Asymmetry parameter 2:	0.000000
Peak shape:	
parameter 1 Left:	0.626711
parameter 2 Left:	0.000000
parameter 3 Left:	0.000000
R (Bragg)/ %:	2.99940

Occupancy, atomic fract. coordinates and Biso for $LiYO_2$-HT, 50993-ICSD

Atom	Wyck.	s.o.f.	x	y	z	B/ 10^4 pm^2
Y1	4b	0.970000	0.000000	0.250000	0.375000	0.000000
Li1	4b	0.000000	0.000000	0.250000	0.375000	0.000000
Li2	4a	1.000000	0.000000	0.750000	0.125000	0.000000
O1	8e	1.000000	0.000000	0.250000	0.152000	0.000000
Yb1	4b	0.020000	0.000000	0.250000	0.375000	0.000000
Er1	4b	0.010000	0.000000	0.250000	0.375000	0.000000

The data for the $LiYO_2$:2%Yb^{3+}, 1%Er^{3+} at 170 K:

Global Parameters

Number of used phases:	3
Number of variables:	20
Number of constraints:	1
Zero shift/ °2Theta:	0.000000
Specimen displacement/ mm :	0.260(2)
Profile function:	Pseudo Voigt
Background:	Polynomial
R (expected)/ %:	0.54611
R (profile)/ %:	3.78278
R (weighted profile)/ %:	5.46816
GOF:	100.25900
d-statistic:	0.14943
U standard:	0.000000
V standard:	0.000000
W standard:	0.010000
U Left:	0.000000
V Left:	0.000000
W Left:	0.010000
U Right:	0.000000
V Right:	0.000000
W Right:	0.010000
Asymmetry Type:	No Asymmetry Function
Asymmetry 1:	0.000000
Asymmetry 2:	0.000000
Shape Type:	Shape Individual
Shape 1 Left:	0.600000
Shape 2 Left:	0.000000
Shape 3 Left:	0.000000
Shape 1 Right:	0.600000
Shape 2 Right:	0.000000
Shape 3 Right:	0.000000
K a1/a2 intensity ratio:	0.500000
K alpha/beta intensity ratio:	0.000000
Crystal Shape Factor K:	1.0000
Instrumental FWHM Curve Type:	Caglioti function
Instr. Gauss Curve Coefficient A:	0.0045(5)
Instr. Gauss Curve Coefficient B:	-0.0032(9)
Instr. Gauss Curve Coefficient C:	0.0046(3)
Instr. Lorentz Curve Coefficient A:	0.0062(7)
Instr. Lorentz Curve Coefficient B:	-0.004(1)
Instr. Lorentz Curve Coefficient C:	0.0064(5)

Relevant parameters of $LiYO_2$-LT, 50992-ICSD

Structure and profile data:	
Formula sum: $Y_{3.88}Li_{4.00}O_{8.00}Yb_{0.08}Er_{0.04}$	
Formula mass/ g/mol:	521.2477
Density (calculated)/ g/cm^3	4.2084
F(000):	235.6400
Weight fraction/ %:	89.4(3)
Space group (No.):	P 1 21/c 1 (14)
Lattice parameters:	
a/ A:	6.1152(2)
b/ A:	6.1860(3)
c/ A:	6.1990(2)
alpha/ °:	90
beta/ °:	118.724(2)
gamma/ °:	90
V/ 10^6 pm^3	205.64070
Overall displacement parameter:	0.000000
Extinction:	0.000000
Flat Plate Absorption Correction:	0.000000
Porosity:	0.000000
Roughness:	0.000000
Fitting mode:	Structure Fit
U Left:	-0.10(1)
V Left:	0.16(1)
W Left:	0.002(2)
Preferred orientation direction/ hkl:	0.00 0.00 1.00

Preferred orientation parameter:	1.000000	parameter 1 Left:	0.41(1)
Asymmetry parameter 1:	0.25(2)	parameter 2 Left:	0.000000
Asymmetry parameter 2:	0.000000	parameter 3 Left:	0.000000
Peak shape:		R (Bragg)/ %:	4.30532

Occupancy, atomic fract. coordinates and Biso for $LiYO_2$-LT, 50992-ICSD

Atom	Wyck.	s.o.f.	x	y	z	B/ 10^4 pm^2
Y1	4e	0.970000	0.266000	0.131200	0.020700	0.210000
Li1	4e	1.000000	0.791000	0.346000	0.068000	0.600000
O1	4e	1.000000	0.045900	0.107000	0.237800	0.280000
O2	4e	1.000000	0.512100	0.328800	0.343900	0.290000
Yb1	4e	0.020000	0.266000	0.131200	0.020700	0.210000
Er1	4e	0.010000	0.266000	0.131200	0.020700	0.210000

Relevant parameters of $LiYO_2$-HT, 50993-ICSD

Structure and profile data:		Extinction:	0.000000
Formula sum: $Y_{3.88}Li_{4.00}O_{8.00}Yb_{0.08}Er_{0.04}$		Flat Plate Absorption Correction:	0.000000
Formula mass/ g/mol:	521.2477	Porosity:	0.000000
Density (calculated)/ g/cm^3	4.2816	Roughness:	0.000000
F(000):	235.6400	Fitting mode:	Structure Fit
Weight fraction/ %:	6.4(2)	U Left:	0.068937
Space group (No.):	I 41/a m d (141)	V Left:	-0.052995
Lattice parameters:		W Left:	0.027085
a/ A:	4.429400	Preferred orientation direction/ hkl:	0.00 0.00 1.00
b/ A:	4.429400	Preferred orientation parameter:	1.000000
c/ A:	10.302300	Asymmetry parameter 1:	0.170069
alpha/ °:	90	Asymmetry parameter 2:	0.000000
beta/ °:	90	Peak shape:	
gamma/ °:	90	parameter 1 Left:	0.626711
V/ 10^6 pm^3	202.12680	parameter 2 Left:	0.000000
Overall displacement parameter:	0.000000	parameter 3 Left:	0.000000
		R (Bragg)/ %:	3.03861

Occupancy, atomic fract. coordinates and Biso for $LiYO_2$-HT, 50993-ICSD

Atom	Wyck.	s.o.f.	x	y	z	B/ 10^4 pm^2
Y1	4b	0.970000	0.000000	0.250000	0.375000	0.000000
Li1	4b	0.000000	0.000000	0.250000	0.375000	0.000000
Li2	4a	1.000000	0.000000	0.750000	0.125000	0.000000
O1	8e	1.000000	0.000000	0.250000	0.152000	0.000000
Yb1	4b	0.020000	0.000000	0.250000	0.375000	0.000000
Er1	4b	0.010000	0.000000	0.250000	0.375000	0.000000

The data for the $LiYO_2$:2%Yb^{3+}, 1%Er^{3+} at 180 K:

Global Parameters

Number of used phases:	3	Asymmetry Type:	No Asymmetry Function
Number of variables:	22		
Number of constraints:	2	Asymmetry 1:	0.000000
Zero shift/ °2Theta:	0.000000	Asymmetry 2:	0.000000
Specimen displacement/ mm :	0.257(2)	Shape Type:	Shape Individual
Profile function:	Pseudo Voigt	Shape 1 Left:	0.600000
Background:	Polynomial	Shape 2 Left:	0.000000
R (expected)/ %:	0.54574	Shape 3 Left:	0.000000
R (profile)/ %:	3.93388	Shape 1 Right:	0.600000
R (weighted profile)/ %:	5.64477	Shape 2 Right:	0.000000
GOF:	106.98510	Shape 3 Right:	0.000000
d-statistic:	0.14358	K a1/a2 intensity ratio:	0.500000
U standard:	0.000000	K alpha/beta intensity ratio:	0.000000
V standard:	0.000000	Crystal Shape Factor K:	1.0000
W standard:	0.010000	Instrumental FWHM Curve Type:	Caglioti function
U Left:	0.000000	Instr. Gauss Curve Coefficient A:	0.0045(5)
V Left:	0.000000	Instr. Gauss Curve Coefficient B:	-0.0032(9)
W Left:	0.010000	Instr. Gauss Curve Coefficient C:	0.0046(3)
U Right:	0.000000	Instr. Lorentz Curve Coefficient A:	0.0062(7)
V Right:	0.000000	Instr. Lorentz Curve Coefficient B:	-0.004(1)
W Right:	0.010000	Instr. Lorentz Curve Coefficient C:	0.0064(5)

Relevant parameters of $LiYO_2$-LT, 50992-ICSD

Structure and profile data:	Formula sum: $Y_{3.88}Li_{4.00}O_{8.00}Yb_{0.08}Er_{0.04}$

Formula mass/ g/mol:	521.2477	Flat Plate Absorption Correction:	0.000000
Density (calculated)/ g/cm^3	4.2088	Porosity:	0.000000
F(000):	235.6400	Roughness:	0.000000
Weight fraction/ %:	88.4(3)	Fitting mode:	Structure Fit
Space group (No.):	P 1 21/c 1 (14)	U Left:	0.13(3)
Lattice parameters:		V Left:	-0.02(2)
a/ A:	6.1143(2)	W Left:	0.031(3)
b/ A:	6.1872(3)	Preferred orientation direction/ hkl:	0.00 0.00 1.00
c/ A:	6.1971(3)	Preferred orientation parameter:	1.000000
alpha/ °:	90	Asymmetry parameter 1:	0.32(2)
beta/ °:	118.707(2)	Asymmetry parameter 2:	0.000000
gamma/ °:	90	Peak shape:	
V/ 10^6 pm^3	205.62280	parameter 1 Left:	0.37(1)
Overall displacement parameter:	0.000000	parameter 2 Left:	0.000000
Extinction:	0.000000	parameter 3 Left:	0.000000
		R (Bragg)/ %:	4.48117

Occupancy, atomic fract. coordinates and Biso for LiYO$_2$-LT, 50992-ICSD

Atom	Wyck.	s.o.f.	x	y	z	B/ 10^4 pm^2
Y1	4e	0.970000	0.266000	0.131200	0.020700	0.210000
Li1	4e	1.000000	0.791000	0.346000	0.068000	0.600000
O1	4e	1.000000	0.045900	0.107000	0.237800	0.280000
O2	4e	1.000000	0.512100	0.328800	0.343900	0.290000
Yb1	4e	0.020000	0.266000	0.131200	0.020700	0.210000
Er1	4e	0.010000	0.266000	0.131200	0.020700	0.210000

Relevant parameters of LiYO$_2$-HT, 50993-ICSD

Structure and profile data:		Extinction:	0.000000
Formula sum: $Y_{3.88}Li_{4.00}O_{8.00}Yb_{0.08}Er_{0.04}$		Flat Plate Absorption Correction:	0.000000
Formula mass/ g/mol:	521.2477	Porosity:	0.000000
Density (calculated)/ g/cm^3	4.2816	Roughness:	0.000000
F(000):	235.6400	Fitting mode:	Structure Fit
Weight fraction/ %:	7.5(2)	U Left:	0.068937
Space group (No.):	I 41/a m d (141)	V Left:	-0.052995
Lattice parameters:		W Left:	0.027085
a/ A:	4.4293(4)	Preferred orientation direction/ hkl:	0.00 0.00 1.00
b/ A:	4.4293(4)	Preferred orientation parameter:	1.000000
c/ A:	10.303(1)	Asymmetry parameter 1:	0.170069
alpha/ °:	90	Asymmetry parameter 2:	0.000000
beta/ °:	90	Peak shape:	
gamma/ °:	90	parameter 1 Left:	0.626711
V/ 10^6 pm^3	202.12640	parameter 2 Left:	0.000000
Overall displacement parameter:	0.000000	parameter 3 Left:	0.000000
		R (Bragg)/ %:	3.47728

Occupancy, atomic fract. coordinates and Biso for LiYO$_2$-HT, 50993-ICSD

Atom	Wyck.	s.o.f.	x	y	z	B/ 10^4 pm^2
Y1	4b	0.970000	0.000000	0.250000	0.375000	0.000000
Li1	4b	0.000000	0.000000	0.250000	0.375000	0.000000
Li2	4a	1.000000	0.000000	0.750000	0.125000	0.000000
O1	8e	1.000000	0.000000	0.250000	0.152000	0.000000
Yb1	4b	0.020000	0.000000	0.250000	0.375000	0.000000
Er1	4b	0.010000	0.000000	0.250000	0.375000	0.000000

The data for the LiYO$_2$:2%Yb^{3+}, 1%Er^{3+} at 190 K:

Global Parameters

Number of used phases:	3	V standard:	0.000000
Number of variables:	22	W standard:	0.010000
Number of constraints:	2	U Left:	0.000000
Zero shift/ °2Theta:	0.000000	V Left:	0.000000
Specimen displacement/ mm :	0.250(2)	W Left:	0.010000
Profile function:	Pseudo Voigt	U Right:	0.000000
Background:	Polynomial	V Right:	0.000000
R (expected)/ %:	0.54573	W Right:	0.010000
R (profile)/ %:	3.85023	Asymmetry Type:	No Asymmetry Function
R (weighted profile)/ %:	5.56494		
GOF:	103.98570	Asymmetry 1:	0.000000
d-statistic:	0.14897	Asymmetry 2:	0.000000
U standard:	0.000000	Shape Type:	Shape Individual

Shape 1 Left:	0.600000	Crystal Shape Factor K:	1.0000
Shape 2 Left:	0.000000	Instrumental FWHM Curve Type:	Caglioti function
Shape 3 Left:	0.000000	Instr. Gauss Curve Coefficient A:	0.0045(5)
Shape 1 Right:	0.600000	Instr. Gauss Curve Coefficient B:	-0.0032(9)
Shape 2 Right:	0.000000	Instr. Gauss Curve Coefficient C:	0.0046(3)
Shape 3 Right:	0.000000	Instr. Lorentz Curve Coefficient A:	0.0062(7)
K a1/a2 intensity ratio:	0.500000	Instr. Lorentz Curve Coefficient B:	-0.004(1)
K alpha/beta intensity ratio:	0.000000	Instr. Lorentz Curve Coefficient C:	0.0064(5)

Relevant parameters of $LiYO_2$-LT, 50992-ICSD

Structure and profile data:		Extinction:	0.000000
Formula sum: $Y_{3.88}Li_{4.00}O_{8.00}Yb_{0.08}Er_{0.04}$		Flat Plate Absorption Correction:	0.000000
Formula mass/ g/mol:	521.2477	Porosity:	0.000000
Density (calculated)/ g/cm^3	4.2082	Roughness:	0.000000
F(000):	235.6400	Fitting mode:	Structure Fit
Weight fraction/ %:	87.6(3)	U Left:	0.13(3)
Space group (No.):	P 1 21/c 1 (14)	V Left:	-0.02(2)
Lattice parameters:		W Left:	0.031(3)
a/ A:	6.1136(2)	Preferred orientation direction/ hkl:	0.00 0.00 1.00
b/ A:	6.1890(3)	Preferred orientation parameter:	1.000000
c/ A:	6.1958(3)	Asymmetry parameter 1:	0.40(2)
alpha/ °:	90	Asymmetry parameter 2:	0.000000
beta/ °:	118.689(2)	Peak shape:	
gamma/ °:	90	parameter 1 Left:	0.37(1)
V/ 10^6 pm^3	205.65440	parameter 2 Left:	0.000000
Overall displacement parameter:	0.000000	parameter 3 Left:	0.000000
		R (Bragg)/ %:	4.38219

Occupancy, atomic fract. coordinates and Biso for $LiYO_2$-LT, 50992-ICSD

Atom	Wyck.	s.o.f.	x	y	z	B/ 10^4 pm^2
Y1	4e	0.970000	0.266000	0.131200	0.020700	0.210000
Li1	4e	1.000000	0.791000	0.346000	0.068000	0.600000
O1	4e	1.000000	0.045900	0.107000	0.237800	0.280000
O2	4e	1.000000	0.512100	0.328800	0.343900	0.290000
Yb1	4e	0.020000	0.266000	0.131200	0.020700	0.210000
Er1	4e	0.010000	0.266000	0.131200	0.020700	0.210000

Relevant parameters of LiYO2-HT, 50993-ICSD

Structure and profile data:		Extinction:	0.000000
Formula sum: $Y_{3.88}Li_{4.00}O_{8.00}Yb_{0.08}Er_{0.04}$		Flat Plate Absorption Correction:	0.000000
Formula mass/ g/mol:	521.2477	Porosity:	0.000000
Density (calculated)/ g/cm^3	4.2789	Roughness:	0.000000
F(000):	235.6400	Fitting mode:	Structure Fit
Weight fraction/ %:	8.3(2)	U Left:	0.068937
Space group (No.):	I 41/a m d (141)	V Left:	-0.052995
Lattice parameters:		W Left:	0.027085
a/ A:	4.4346(3)	Preferred orientation direction/ hkl:	0.00 0.00 1.00
b/ A:	4.4346(3)	Preferred orientation parameter:	1.000000
c/ A:	10.285(1)	Asymmetry parameter 1:	0.170069
alpha/ °:	90	Asymmetry parameter 2:	0.000000
beta/ °:	90	Peak shape:	
gamma/ °:	90	parameter 1 Left:	0.626711
V/ 10^6 pm^3	202.25310	parameter 2 Left:	0.000000
Overall displacement parameter:	0.000000	parameter 3 Left:	0.000000
		R (Bragg)/ %:	3.13164

Occupancy, atomic fract. coordinates and Biso for $LiYO_2$-HT, 50993-ICSD

Atom	Wyck.	s.o.f.	x	y	z	B/ 10^4 pm^2
Y1	4b	0.970000	0.000000	0.250000	0.375000	0.000000
Li1	4b	0.000000	0.000000	0.250000	0.375000	0.000000
Li2	4a	1.000000	0.000000	0.750000	0.125000	0.000000
O1	8e	1.000000	0.000000	0.250000	0.152000	0.000000
Yb1	4b	0.020000	0.000000	0.250000	0.375000	0.000000
Er1	4b	0.010000	0.000000	0.250000	0.375000	0.000000

The data for the $LiYO_2$:2%Yb^{3+}, 1%Er^{3+} at 200 K:

Global Parameters

Number of used phases:	3	Number of constraints:	2
Number of variables:	22	Zero shift/ °2Theta:	0.000000

Specimen displacement/ mm :	0.246(2)	Asymmetry 1:	0.000000
Profile function:	Pseudo Voigt	Asymmetry 2:	0.000000
Background:	Polynomial	Shape Type:	Shape Individual
R (expected)/ %:	0.54393	Shape 1 Left:	0.600000
R (profile)/ %:	3.91754	Shape 2 Left:	0.000000
R (weighted profile)/ %:	5.63832	Shape 3 Left:	0.000000
GOF:	107.45100	Shape 1 Right:	0.600000
d-statistic:	0.14582	Shape 2 Right:	0.000000
U standard:	0.000000	Shape 3 Right:	0.000000
V standard:	0.000000	K a1/a2 intensity ratio:	0.500000
W standard:	0.010000	K alpha/beta intensity ratio:	0.000000
U Left:	0.000000	Crystal Shape Factor K:	1.0000
V Left:	0.000000	Instrumental FWHM Curve Type:	Caglioti function
W Left:	0.010000	Instr. Gauss Curve Coefficient A:	0.0045(5)
U Right:	0.000000	Instr. Gauss Curve Coefficient B:	-0.0032(9)
V Right:	0.000000	Instr. Gauss Curve Coefficient C:	0.0046(3)
W Right:	0.010000	Instr. Lorentz Curve Coefficient A:	0.0062(7)
Asymmetry Type:	No Asymmetry Function	Instr. Lorentz Curve Coefficient B:	-0.004(1)
		Instr. Lorentz Curve Coefficient C:	0.0064(5)

Relevant parameters of $LiYO_2$-LT, 50992-ICSD

Structure and profile data:		Extinction:	0.000000
Formula sum: $Y_{3.88}Li_{4.00}O_{8.00}Yb_{0.08}Er_{0.04}$		Flat Plate Absorption Correction:	0.000000
Formula mass/ g/mol:	521.2477	Porosity:	0.000000
Density (calculated)/ g/cm^3	4.2082	Roughness:	0.000000
F(000):	235.6400	Fitting mode:	Structure Fit
Weight fraction/ %:	86.0(3)	U Left:	0.13(3)
Space group (No.):	P 1 21/c 1 (14)	V Left:	-0.02(2)
Lattice parameters:		W Left:	0.031(3)
a/ A:	6.1130(2)	Preferred orientation direction/ hkl:	0.00 0.00 1.00
b/ A:	6.1905(3)	Preferred orientation parameter:	1.000000
c/ A:	6.1944(3)	Asymmetry parameter 1:	0.39(2)
alpha/ °:	90	Asymmetry parameter 2:	0.000000
beta/ °:	118.680(3)	Peak shape:	
gamma/ °:	90	parameter 1 Left:	0.36(1)
V/ 10^6 pm^3	205.65350	parameter 2 Left:	0.000000
Overall displacement parameter:	0.000000	parameter 3 Left:	0.000000
		R (Bragg)/ %:	4.50199

Occupancy, atomic fract. coordinates and Biso for $LiYO_2$-LT, 50992-ICSD

Atom	Wyck.	s.o.f.	x	y	z	B/ 10^4 pm^2
Y1	4e	0.970000	0.266000	0.131200	0.020700	0.210000
Li1	4e	1.000000	0.791000	0.346000	0.068000	0.600000
O1	4e	1.000000	0.045900	0.107000	0.237800	0.280000
O2	4e	1.000000	0.512100	0.328800	0.343900	0.290000
Yb1	4e	0.020000	0.266000	0.131200	0.020700	0.210000
Er1	4e	0.010000	0.266000	0.131200	0.020700	0.210000

Relevant parameters of $LiYO_2$-HT, 50993-ICSD

Structure and profile data:		Extinction:	0.000000
Formula sum: $Y_{3.88}Li_{4.00}O_{8.00}Yb_{0.08}Er_{0.04}$		Flat Plate Absorption Correction:	0.000000
Formula mass/ g/mol:	521.2477	Porosity:	0.000000
Density (calculated)/ g/cm^3	4.2722	Roughness:	0.000000
F(000):	235.6400	Fitting mode:	Structure Fit
Weight fraction/ %:	9.9(2)	U Left:	0.068937
Space group (No.):	I 41/a m d (141)	V Left:	-0.052995
Lattice parameters:		W Left:	0.027085
a/ A:	4.4374(3)	Preferred orientation direction/ hkl:	0.00 0.00 1.00
b/ A:	4.4374(3)	Preferred orientation parameter:	1.000000
c/ A:	10.2878(9)	Asymmetry parameter 1:	0.170069
alpha/ °:	90	Asymmetry parameter 2:	0.000000
beta/ °:	90	Peak shape:	
gamma/ °:	90	parameter 1 Left:	0.626711
V/ 10^6 pm^3	202.57020	parameter 2 Left:	0.000000
Overall displacement parameter:	0.000000	parameter 3 Left:	0.000000
		R (Bragg)/ %:	3.33383

Occupancy, atomic fract. coordinates and Biso for $LiYO_2$-HT, 50993-ICSD

Atom	Wyck.	s.o.f.	x	y	z	B/ 10^4 pm^2
Y1	4b	0.970000	0.000000	0.250000	0.375000	0.000000
Li1	4b	0.000000	0.000000	0.250000	0.375000	0.000000

Li2	4a	1.000000	0.000000	0.750000	0.125000	0.000000
O1	8e	1.000000	0.000000	0.250000	0.152000	0.000000
Yb1	4b	0.020000	0.000000	0.250000	0.375000	0.000000
Er1	4b	0.010000	0.000000	0.250000	0.375000	0.000000

The data for the $LiYO_2$:2%Yb^{3+}, 1%Er^{3+} at 210 K:

Global Parameters

Number of used phases:	3	Asymmetry Type:	No Asymmetry Function
Number of variables:	22		
Number of constraints:	2	Asymmetry 1:	0.000000
Zero shift/ °2Theta:	0.000000	Asymmetry 2:	0.000000
Specimen displacement/ mm :	0.239(2)	Shape Type:	Shape Individual
Profile function:	Pseudo Voigt	Shape 1 Left:	0.600000
Background:	Polynomial	Shape 2 Left:	0.000000
R (expected)/ %:	0.54507	Shape 3 Left:	0.000000
R (profile)/ %:	3.86800	Shape 1 Right:	0.600000
R (weighted profile)/ %:	5.57989	Shape 2 Right:	0.000000
GOF:	104.79480	Shape 3 Right:	0.000000
d-statistic:	0.14698	K a1/a2 intensity ratio:	0.500000
U standard:	0.000000	K alpha/beta intensity ratio:	0.000000
V standard:	0.000000	Crystal Shape Factor K:	1.0000
W standard:	0.010000	Instrumental FWHM Curve Type:	Caglioti function
U Left:	0.000000	Instr. Gauss Curve Coefficient A:	0.0045(5)
V Left:	0.000000	Instr. Gauss Curve Coefficient B:	-0.0032(9)
W Left:	0.010000	Instr. Gauss Curve Coefficient C:	0.0046(3)
U Right:	0.000000	Instr. Lorentz Curve Coefficient A:	0.0062(7)
V Right:	0.000000	Instr. Lorentz Curve Coefficient B:	-0.004(1)
W Right:	0.010000	Instr. Lorentz Curve Coefficient C:	0.0064(5)

Relevant parameters of $LiYO_2$-LT, 50992-ICSD

Structure and profile data:		Extinction:	0.000000
Formula sum: $Y_{3.88}Li_{4.00}O_{8.00}Yb_{0.08}Er_{0.04}$		Flat Plate Absorption Correction:	0.000000
Formula mass/ g/mol:	521.2477	Porosity:	0.000000
Density (calculated)/ g/cm^3	4.2077	Roughness:	0.000000
F(000):	235.6400	Fitting mode:	Structure Fit
Weight fraction/ %:	83.2(3)	U Left:	0.13(3)
Space group (No.):	P 1 21/c 1 (14)	V Left:	-0.02(2)
Lattice parameters:		W Left:	0.032(3)
a/ A:	6.1124(2)	Preferred orientation direction/ hkl:	0.00 0.00 1.00
b/ A:	6.1925(3)	Preferred orientation parameter:	1.000000
c/ A:	6.1927(3)	Asymmetry parameter 1:	0.36(2)
alpha/ °:	90	Asymmetry parameter 2:	0.000000
beta/ °:	118.662(3)	Peak shape:	
gamma/ °:	90	parameter 1 Left:	0.36(1)
V/ 10^6 pm^3	205.67610	parameter 2 Left:	0.000000
Overall displacement parameter:	0.000000	parameter 3 Left:	0.000000
		R (Bragg)/ %:	4.41107

Occupancy, atomic fract. coordinates and Biso for $LiYO_2$-LT, 50992-ICSD

Atom	Wyck.	s.o.f.	x	y	z	B/ 10^4 pm^2
Y1	4e	0.970000	0.266000	0.131200	0.020700	0.210000
Li1	4e	1.000000	0.791000	0.346000	0.068000	0.600000
O1	4e	1.000000	0.045900	0.107000	0.237800	0.280000
O2	4e	1.000000	0.512100	0.328800	0.343900	0.290000
Yb1	4e	0.020000	0.266000	0.131200	0.020700	0.210000
Er1	4e	0.010000	0.266000	0.131200	0.020700	0.210000

Relevant parameters of $LiYO_2$-HT, 50993-ICSD

Structure and profile data:		alpha/ °:	90
Formula sum: $Y_{3.88}Li_{4.00}O_{8.00}Yb_{0.08}Er_{0.04}$		beta/ °:	90
Formula mass/ g/mol:	521.2477	gamma/ °:	90
Density (calculated)/ g/cm^3	4.2671	V/ 10^6 pm^3	202.81290
F(000):	235.6400	Overall displacement parameter:	0.000000
Weight fraction/ %:	12.5(2)	Extinction:	0.000000
Space group (No.):	I 41/a m d (141)	Flat Plate Absorption Correction:	0.000000
Lattice parameters:		Porosity:	0.000000
a/ A:	4.4403(2)	Roughness:	0.000000
b/ A:	4.4403(2)	Fitting mode:	Structure Fit
c/ A:	10.2867(8)	U Left:	0.068937
		V Left:	-0.052995

W Left:	0.027085	Peak shape:	
Preferred orientation direction/ hkl:	0.00 0.00 1.00	parameter 1 Left:	0.626711
Preferred orientation parameter:	1.000000	parameter 2 Left:	0.000000
Asymmetry parameter 1:	0.170069	parameter 3 Left:	0.000000
Asymmetry parameter 2:	0.000000	R (Bragg)/ %:	3.89554

Occupancy, atomic fract. coordinates and Biso for $LiYO_2$-HT, 50993-ICSD

Atom	Wyck.	s.o.f.	x	y	z	B/ 10^4 pm^2
Y1	4b	0.970000	0.000000	0.250000	0.375000	0.000000
Li1	4b	0.000000	0.000000	0.250000	0.375000	0.000000
Li2	4a	1.000000	0.000000	0.750000	0.125000	0.000000
O1	8e	1.000000	0.000000	0.250000	0.152000	0.000000
Yb1	4b	0.020000	0.000000	0.250000	0.375000	0.000000
Er1	4b	0.010000	0.000000	0.250000	0.375000	0.000000

The data for the $LiYO_2$:2%Yb^{3+}, 1%Er^{3+} at 220 K:

Global Parameters

Number of used phases:	3	Asymmetry Type:	No Asymmetry Function
Number of variables:	22		
Number of constraints:	2	Asymmetry 1:	0.000000
Zero shift/ °2Theta:	0.000000	Asymmetry 2:	0.000000
Specimen displacement/ mm :	0.233(2)	Shape Type:	Shape Individual
Profile function:	Pseudo Voigt	Shape 1 Left:	0.600000
Background:	Polynomial	Shape 2 Left:	0.000000
R (expected)/ %:	0.54548	Shape 3 Left:	0.000000
R (profile)/ %:	3.76495	Shape 1 Right:	0.600000
R (weighted profile)/ %:	5.42485	Shape 2 Right:	0.000000
GOF:	98.90562	Shape 3 Right:	0.000000
d-statistic:	0.16454	K a1/a2 intensity ratio:	0.500000
U standard:	0.000000	K alpha/beta intensity ratio:	0.000000
V standard:	0.000000	Crystal Shape Factor K:	1.0000
W standard:	0.010000	Instrumental FWHM Curve Type:	Caglioti function
U Left:	0.000000	Instr. Gauss Curve Coefficient A:	0.0045(5)
V Left:	0.000000	Instr. Gauss Curve Coefficient B:	-0.0032(9)
W Left:	0.010000	Instr. Gauss Curve Coefficient C:	0.0046(3)
U Right:	0.000000	Instr. Lorentz Curve Coefficient A:	0.0062(7)
V Right:	0.000000	Instr. Lorentz Curve Coefficient B:	-0.004(1)
W Right:	0.010000	Instr. Lorentz Curve Coefficient C:	0.0064(5)

Relevant parameters of $LiYO_2$-LT, 50992-ICSD

Structure and profile data:		Extinction:	0.000000
Formula sum: $Y_{3.88}Li_{4.00}O_{8.00}Yb_{0.08}Er_{0.04}$		Flat Plate Absorption Correction:	0.000000
Formula mass/ g/mol:	521.2477	Porosity:	0.000000
Density (calculated)/ g/cm^3	4.2076	Roughness:	0.000000
F(000):	235.6400	Fitting mode:	Structure Fit
Weight fraction/ %:	79.8(3)	U Left:	0.10(2)
Space group (No.):	P 1 21/c 1 (14)	V Left:	-0.02(2)
Lattice parameters:		W Left:	0.034(3)
a/ A:	6.1119(2)	Preferred orientation direction/ hkl:	0.00 0.00 1.00
b/ A:	6.1943(3)	Preferred orientation parameter:	1.000000
c/ A:	6.1906(2)	Asymmetry parameter 1:	0.44(3)
alpha/ °:	90	Asymmetry parameter 2:	0.000000
beta/ °:	118.647(2)	Peak shape:	
gamma/ °:	90	parameter 1 Left:	0.35(1)
V/ 10^6 pm^3	205.68130	parameter 2 Left:	0.000000
Overall displacement parameter:	0.000000	parameter 3 Left:	0.000000
		R (Bragg)/ %:	4.18055

Occupancy, atomic fract. coordinates and Biso for $LiYO_2$-LT, 50992-ICSD

Atom	Wyck.	s.o.f.	x	y	z	B/ 10^4 pm^2
Y1	4e	0.970000	0.266000	0.131200	0.020700	0.210000
Li1	4e	1.000000	0.791000	0.346000	0.068000	0.600000
O1	4e	1.000000	0.045900	0.107000	0.237800	0.280000
O2	4e	1.000000	0.512100	0.328800	0.343900	0.290000
Yb1	4e	0.020000	0.266000	0.131200	0.020700	0.210000
Er1	4e	0.010000	0.266000	0.131200	0.020700	0.210000

Relevant parameters of $LiYO_2$-HT, 50993-ICSD

Structure and profile data:		Formula sum: $Y_{3.88}Li_{4.00}O_{8.00}Yb_{0.08}Er_{0.04}$	

Formula mass/ g/mol:	521.2477	Flat Plate Absorption Correction:	0.000000
Density (calculated)/ g/cm^3	4.2625	Porosity:	0.000000
F(000):	235.6400	Roughness:	0.000000
Weight fraction/ %:	16.0(2)	Fitting mode:	Structure Fit
Space group (No.):	I 41/a m d (141)	U Left:	0.068937
Lattice parameters:		V Left:	-0.052995
a/ A:	4.4419(2)	W Left:	0.027085
b/ A:	4.4419(2)	Preferred orientation direction/ hkl:	0.00 0.00 1.00
c/ A:	10.2903(6)	Preferred orientation parameter:	1.000000
alpha/ °:	90	Asymmetry parameter 1:	0.170069
beta/ °:	90	Asymmetry parameter 2:	0.000000
gamma/ °:	90	Peak shape:	
V/ 10^6 pm^3	203.03420	parameter 1 Left:	0.626711
Overall displacement parameter:	0.000000	parameter 2 Left:	0.000000
Extinction:	0.000000	parameter 3 Left:	0.000000
		R (Bragg)/ %:	3.8692

Occupancy, atomic fract. coordinates and Biso for LiYO$_2$-HT, 50993-ICSD

Atom	Wyck.	s.o.f.	x	y	z	B/ 10^4 pm^2
Y1	4b	0.970000	0.000000	0.250000	0.375000	0.000000
Li1	4b	0.000000	0.000000	0.250000	0.375000	0.000000
Li2	4a	1.000000	0.000000	0.750000	0.125000	0.000000
O1	8e	1.000000	0.000000	0.250000	0.152000	0.000000
Yb1	4b	0.020000	0.000000	0.250000	0.375000	0.000000
Er1	4b	0.010000	0.000000	0.250000	0.375000	0.000000

The data for the LiYO$_2$:2%Yb^{3+}, 1%Er^{3+} at 230 K:

Global Parameters

Number of used phases:	3		Function
Number of variables:	22	Asymmetry 1:	0.000000
Number of constraints:	2	Asymmetry 2:	0.000000
Zero shift/ °2Theta:	0.000000	Shape Type:	Shape Individual
Specimen displacement/ mm :	0.251(1)	Shape 1 Left:	0.600000
Profile function:	Pseudo Voigt	Shape 2 Left:	0.000000
Background:	Polynomial	Shape 3 Left:	0.000000
R (expected)/ %:	0.53567	Shape 1 Right:	0.600000
R (profile)/ %:	3.67564	Shape 2 Right:	0.000000
R (weighted profile)/ %:	5.02388	Shape 3 Right:	0.000000
GOF:	87.95968	K a1/a2 intensity ratio:	0.500000
d-statistic:	0.19748	K alpha/beta intensity ratio:	0.000000
U standard:	0.000000	Crystal Shape Factor K:	1.0000
V standard:	0.000000	Instrumental FWHM Curve Type:	Caglioti function
W standard:	0.010000	Instr. Gauss Curve Coefficient A:	0.0045(5)
U Left:	0.000000	Instr. Gauss Curve Coefficient B:	-0.0032(9)
V Left:	0.000000	Instr. Gauss Curve Coefficient C:	0.0046(3)
W Left:	0.010000	Instr. Lorentz Curve Coefficient A:	0.0062(7)
U Right:	0.000000	Instr. Lorentz Curve Coefficient B:	-0.004(1)
V Right:	0.000000	Instr. Lorentz Curve Coefficient C:	0.0064(5)
W Right:	0.010000		
Asymmetry Type:	No Asymmetry		

Relevant parameters of LiYO$_2$-LT, 50992-ICSD

Structure and profile data:		Extinction:	0.000000
Formula sum: $Y_{3.88}Li_{4.00}O_{8.00}Yb_{0.08}Er_{0.04}$		Flat Plate Absorption Correction:	0.000000
Formula mass/ g/mol:	521.2477	Porosity:	0.000000
Density (calculated)/ g/cm^3	4.2056	Roughness:	0.000000
F(000):	235.6400	Fitting mode:	Structure Fit
Weight fraction/ %:	74.7(3)	U Left:	0.07(2)
Space group (No.):	P 1 21/c 1 (14)	V Left:	-0.02(1)
Lattice parameters:		W Left:	0.030(2)
a/ A:	6.1124(2)	Preferred orientation direction/ hkl:	0.00 0.00 1.00
b/ A:	6.1965(2)	Preferred orientation parameter:	1.000000
c/ A:	6.1908(2)	Asymmetry parameter 1:	0.52(2)
alpha/ °:	90	Asymmetry parameter 2:	0.000000
beta/ °:	118.645(2)	Peak shape:	
gamma/ °:	90	parameter 1 Left:	0.50(1)
V/ 10^6 pm^3	205.78180	parameter 2 Left:	0.000000
Overall displacement parameter:	0.000000	parameter 3 Left:	0.000000
		R (Bragg)/ %:	3.31612

Occupancy, atomic fract. coordinates and Biso for $LiYO_2$-LT, 50992-ICSD

Atom	Wyck.	s.o.f.	x	y	z	B/ 10^4 pm^2
Y1	4e	0.970000	0.266000	0.131200	0.020700	0.210000
Li1	4e	1.000000	0.791000	0.346000	0.068000	0.600000
O1	4e	1.000000	0.045900	0.107000	0.237800	0.280000
O2	4e	1.000000	0.512100	0.328800	0.343900	0.290000
Yb1	4e	0.020000	0.266000	0.131200	0.020700	0.210000
Er1	4e	0.010000	0.266000	0.131200	0.020700	0.210000

Relevant parameters of $LiYO_2$-HT, 50993-ICSD

Structure and profile data:	
Formula sum: $Y_{3.88}Li_{4.00}O_{8.00}Yb_{0.08}Er_{0.04}$	
Formula mass/ g/mol:	521.2477
Density (calculated)/ g/cm^3	4.2559
F(000):	235.6400
Weight fraction/ %:	20.5(2)
Space group (No.):	I 41/a m d (141)
Lattice parameters:	
a/ A:	4.4432(1)
b/ A:	4.4432(1)
c/ A:	10.3001(4)
alpha/ °:	90
beta/ °:	90
gamma/ °:	90
V/ 10^6 pm^3	203.34780
Overall displacement parameter:	0.000000
Extinction:	0.000000
Flat Plate Absorption Correction:	0.000000
Porosity:	0.000000
Roughness:	0.000000
Fitting mode:	Structure Fit
U Left:	0.068937
V Left:	-0.052995
W Left:	0.027085
Preferred orientation direction/ hkl:	0.00 0.00 1.00
Preferred orientation parameter:	1.000000
Asymmetry parameter 1:	0.170069
Asymmetry parameter 2:	0.000000
Peak shape:	
parameter 1 Left:	0.626711
parameter 2 Left:	0.000000
parameter 3 Left:	0.000000
R (Bragg)/ %:	2.94528

Occupancy, atomic fract. coordinates and Biso for $LiYO_2$-HT, 50993-ICSD

Atom	Wyck.	s.o.f.	x	y	z	B/ 10^4 pm^2
Y1	4b	0.970000	0.000000	0.250000	0.375000	0.000000
Li1	4b	0.000000	0.000000	0.250000	0.375000	0.000000
Li2	4a	1.000000	0.000000	0.750000	0.125000	0.000000
O1	8e	1.000000	0.000000	0.250000	0.152000	0.000000
Yb1	4b	0.020000	0.000000	0.250000	0.375000	0.000000
Er1	4b	0.010000	0.000000	0.250000	0.375000	0.000000

The data for the $LiYO_2$:2%Yb^{3+}, 1%Er^{3+} at 240 K:

Global Parameters

Number of used phases:	3
Number of variables:	26
Number of constraints:	2
Zero shift/ °2Theta:	0.000000
Specimen displacement/ mm :	0.217(1)
Profile function:	Pseudo Voigt
Background:	Polynomial
R (expected)/ %:	0.54499
R (profile)/ %:	3.57660
R (weighted profile)/ %:	5.06205
GOF:	86.27329
d-statistic:	0.21051
U standard:	0.000000
V standard:	0.000000
W standard:	0.010000
U Left:	0.000000
V Left:	0.000000
W Left:	0.010000
U Right:	0.000000
V Right:	0.000000
W Right:	0.010000
Asymmetry Type:	No Asymmetry Function
Asymmetry 1:	0.000000
Asymmetry 2:	0.000000
Shape Type:	Shape Individual
Shape 1 Left:	0.600000
Shape 2 Left:	0.000000
Shape 3 Left:	0.000000
Shape 1 Right:	0.600000
Shape 2 Right:	0.000000
Shape 3 Right:	0.000000
K a1/a2 intensity ratio:	0.500000
K alpha/beta intensity ratio:	0.000000
Crystal Shape Factor K:	1.0000
Instrumental FWHM Curve Type:	Caglioti function
Instr. Gauss Curve Coefficient A:	0.0045(5)
Instr. Gauss Curve Coefficient B:	-0.0032(9)
Instr. Gauss Curve Coefficient C:	0.0046(3)
Instr. Lorentz Curve Coefficient A:	0.0062(7)
Instr. Lorentz Curve Coefficient B:	-0.004(1)
Instr. Lorentz Curve Coefficient C:	0.0064(5)

Relevant parameters of $LiYO_2$-LT, 50992-ICSD

Structure and profile data:	
Formula sum: $Y_{3.88}Li_{4.00}O_{8.00}Yb_{0.08}Er_{0.04}$	
Formula mass/ g/mol:	521.2477
Density (calculated)/ g/cm^3	4.2067
F(000):	235.6400
Weight fraction/ %:	67.2(4)
Space group (No.):	P 1 21/c 1 (14)
Lattice parameters:	
a/ A:	6.1111(2)
b/ A:	6.1972(2)
c/ A:	6.1877(2)
alpha/ °:	90
beta/ °:	118.609(2)

gamma/ °:	90	W Left:	0.039(2)
V/ 10^6 pm^3	205.72590	Preferred orientation direction/ hkl:	0.00 0.00 1.00
Overall displacement parameter:	0.000000	Preferred orientation parameter:	1.000000
Extinction:	0.000000	Asymmetry parameter 1:	0.61(3)
Flat Plate Absorption Correction:	0.000000	Asymmetry parameter 2:	0.000000
Porosity:	0.000000	Peak shape:	
Roughness:	0.000000	parameter 1 Left:	0.39(2)
Fitting mode:	Structure Fit	parameter 2 Left:	0.000000
U Left:	0.04(1)	parameter 3 Left:	0.000000
V Left:	-0.03(1)	R (Bragg)/ %:	3.80401

Occupancy, atomic fract. coordinates and Biso for $LiYO_2$-LT, 50992-ICSD

Atom	Wyck.	s.o.f.	x	y	z	B/ 10^4 pm^2
Y1	4e	0.970000	0.266000	0.131200	0.020700	0.210000
Li1	4e	1.000000	0.791000	0.346000	0.068000	0.600000
O1	4e	1.000000	0.045900	0.107000	0.237800	0.280000
O2	4e	1.000000	0.512100	0.328800	0.343900	0.290000
Yb1	4e	0.020000	0.266000	0.131200	0.020700	0.210000
Er1	4e	0.010000	0.266000	0.131200	0.020700	0.210000

Relevant parameters of $LiYO_2$-HT, 50993-ICSD

Structure and profile data:		Extinction:	0.000000
Formula sum: $Y_{3.88}Li_{4.00}O_{8.00}Yb_{0.08}Er_{0.04}$		Flat Plate Absorption Correction:	0.000000
Formula mass/ g/mol:	521.2477	Porosity:	0.000000
Density (calculated)/ g/cm^3	4.2577	Roughness:	0.000000
F(000):	235.6400	Fitting mode:	Structure Fit
Weight fraction/ %:	29.0(3)	U Left:	0.08(2)
Space group (No.):	I 41/a m d (141)	V Left:	-0.03(1)
Lattice parameters:		W Left:	0.016(2)
a/ A:	4.4418(1)	Preferred orientation direction/ hkl:	0.00 0.00 1.00
b/ A:	4.4418(1)	Preferred orientation parameter:	1.000000
c/ A:	10.3023(4)	Asymmetry parameter 1:	0.000000
alpha/ °:	90	Asymmetry parameter 2:	0.000000
beta/ °:	90	Peak shape:	
gamma/ °:	90	parameter 1 Left:	0.69(3)
V/ 10^6 pm^3	203.26230	parameter 2 Left:	0.000000
Overall displacement parameter:	0.000000	parameter 3 Left:	0.000000
		R (Bragg)/ %:	3.99658

Occupancy, atomic fract. coordinates and Biso for $LiYO_2$-HT, 50993-ICSD

Atom	Wyck.	s.o.f.	x	y	z	B/ 10^4 pm^2
Y1	4b	0.970000	0.000000	0.250000	0.375000	0.000000
Li1	4b	0.000000	0.000000	0.250000	0.375000	0.000000
Li2	4a	1.000000	0.000000	0.750000	0.125000	0.000000
O1	8e	1.000000	0.000000	0.250000	0.152000	0.000000
Yb1	4b	0.020000	0.000000	0.250000	0.375000	0.000000
Er1	4b	0.010000	0.000000	0.250000	0.375000	0.000000

The data for the $LiYO_2$:2%Yb^{3+}, 1%Er^{3+} at 250 K:

Global Parameters

Number of used phases:	3	V Right:	0.000000
Number of variables:	26	W Right:	0.010000
Number of constraints:	2	Asymmetry Type:	No Asymmetry Function
Zero shift/ °2Theta:	0.000000		
Specimen displacement/ mm :	0.240(1)	Asymmetry 1:	0.000000
Profile function:	Pseudo Voigt	Asymmetry 2:	0.000000
Background:	Polynomial	Shape Type:	Shape Individual
R (expected)/ %:	0.53513	Shape 1 Left:	0.600000
R (profile)/ %:	3.37845	Shape 2 Left:	0.000000
R (weighted profile)/ %:	4.62895	Shape 3 Left:	0.000000
GOF:	74.82628	Shape 1 Right:	0.600000
d-statistic:	0.26526	Shape 2 Right:	0.000000
U standard:	0.000000	Shape 3 Right:	0.000000
V standard:	0.000000	K a1/a2 intensity ratio:	0.500000
W standard:	0.010000	K alpha/beta intensity ratio:	0.000000
U Left:	0.000000	Crystal Shape Factor K:	1.0000
V Left:	0.000000	Instrumental FWHM Curve Type:	Caglioti function
W Left:	0.010000	Instr. Gauss Curve Coefficient A:	0.0045(5)
U Right:	0.000000	Instr. Gauss Curve Coefficient B:	-0.0032(9)

Instr. Gauss Curve Coefficient C:	0.0046(3)	Instr. Lorentz Curve Coefficient B:	-0.004(1)
Instr. Lorentz Curve Coefficient A:	0.0062(7)	Instr. Lorentz Curve Coefficient C:	0.0064(5)

Relevant parameters of $LiYO_2$-LT, 50992-ICSD

Structure and profile data:		Extinction:	0.000000
Formula sum: $Y_{3.88}Li_{4.00}O_{8.00}Yb_{0.08}Er_{0.04}$		Flat Plate Absorption Correction:	0.000000
Formula mass/ g/mol:	521.2477	Porosity:	0.000000
Density (calculated)/ g/cm^3	4.2064	Roughness:	0.000000
F(000):	235.6400	Fitting mode:	Structure Fit
Weight fraction/ %:	58.9(3)	U Left:	0.10(1)
Space group (No.):	P 1 21/c 1 (14)	V Left:	-0.08(1)
Lattice parameters:		W Left:	0.038(2)
a/ A:	6.1109(2)	Preferred orientation direction/ hkl:	0.00 0.00 1.00
b/ A:	6.1981(2)	Preferred orientation parameter:	1.000000
c/ A:	6.1875(2)	Asymmetry parameter 1:	0.91(3)
alpha/ °:	90	Asymmetry parameter 2:	0.000000
beta/ °:	118.610(2)	Peak shape:	
gamma/ °:	90	parameter 1 Left:	0.47(2)
V/ 10^6 pm^3	205.73890	parameter 2 Left:	0.000000
Overall displacement parameter:	0.000000	parameter 3 Left:	0.000000
		R (Bragg)/ %:	2.69684

Occupancy, atomic fract. coordinates and Biso for $LiYO_2$-LT, 50992-ICSD

Atom	Wyck.	s.o.f.	x	y	z	B/ 10^4 pm^2
Y1	4e	0.970000	0.266000	0.131200	0.020700	0.210000
Li1	4e	1.000000	0.791000	0.346000	0.068000	0.600000
O1	4e	1.000000	0.045900	0.107000	0.237800	0.280000
O2	4e	1.000000	0.512100	0.328800	0.343900	0.290000
Yb1	4e	0.020000	0.266000	0.131200	0.020700	0.210000
Er1	4e	0.010000	0.266000	0.131200	0.020700	0.210000

Relevant parameters of $LiYO_2$-HT, 50993-ICSD

Structure and profile data:		Extinction:	0.000000
Formula sum: $Y_{3.88}Li_{4.00}O_{8.00}Yb_{0.08}Er_{0.04}$		Flat Plate Absorption Correction:	0.000000
Formula mass/ g/mol:	521.2477	Porosity:	0.000000
Density (calculated)/ g/cm^3	4.2523	Roughness:	0.000000
F(000):	235.6400	Fitting mode:	Structure Fit
Weight fraction/ %:	36.5(3)	U Left:	0.04(1)
Space group (No.):	I 41/a m d (141)	V Left:	-0.013(8)
Lattice parameters:		W Left:	0.015(1)
a/ A:	4.4427(1)	Preferred orientation direction/ hkl:	0.00 0.00 1.00
b/ A:	4.4427(1)	Preferred orientation parameter:	1.000000
c/ A:	10.3115(3)	Asymmetry parameter 1:	0.000000
alpha/ °:	90	Asymmetry parameter 2:	0.000000
beta/ °:	90	Peak shape:	
gamma/ °:	90	parameter 1 Left:	0.75(2)
V/ 10^6 pm^3	203.51910	parameter 2 Left:	0.000000
Overall displacement parameter:	0.000000	parameter 3 Left:	0.000000
		R (Bragg)/ %:	3.22526

Occupancy, atomic fract. coordinates and Biso for $LiYO_2$-HT, 50993-ICSD

Atom	Wyck.	s.o.f.	x	y	z	B/ 10^4 pm^2
Y1	4b	0.970000	0.000000	0.250000	0.375000	0.000000
Li1	4b	0.000000	0.000000	0.250000	0.375000	0.000000
Li2	4a	1.000000	0.000000	0.750000	0.125000	0.000000
O1	8e	1.000000	0.000000	0.250000	0.152000	0.000000
Yb1	4b	0.020000	0.000000	0.250000	0.375000	0.000000
Er1	4b	0.010000	0.000000	0.250000	0.375000	0.000000

The data for the $LiYO_2$:2%Yb^{3+}, 1%Er^{3+} at 260 K:

Global Parameters

Number of used phases:	3	R (weighted profile)/ %:	4.52816
Number of variables:	26	GOF:	68.84109
Number of constraints:	2	d-statistic:	0.26004
Zero shift/ °2Theta:	0.000000	U standard:	0.000000
Specimen displacement/ mm :	0.195(1)	V standard:	0.000000
Profile function:	Pseudo Voigt	W standard:	0.010000
Background:	Polynomial	U Left:	0.000000
R (expected)/ %:	0.54576	V Left:	0.000000
R (profile)/ %:	3.27273	W Left:	0.010000

U Right:	0.000000	Shape 2 Right:	0.000000
V Right:	0.000000	Shape 3 Right:	0.000000
W Right:	0.010000	K a1/a2 intensity ratio:	0.500000
Asymmetry Type:	No Asymmetry Function	K alpha/beta intensity ratio:	0.000000
		Crystal Shape Factor K:	1.0000
Asymmetry 1:	0.000000	Instrumental FWHM Curve Type:	Caglioti function
Asymmetry 2:	0.000000	Instr. Gauss Curve Coefficient A:	0.0045(5)
Shape Type:	Shape Individual	Instr. Gauss Curve Coefficient B:	-0.0032(9)
Shape 1 Left:	0.600000	Instr. Gauss Curve Coefficient C:	0.0046(3)
Shape 2 Left:	0.000000	Instr. Lorentz Curve Coefficient A:	0.0062(7)
Shape 3 Left:	0.000000	Instr. Lorentz Curve Coefficient B:	-0.004(1)
Shape 1 Right:	0.600000	Instr. Lorentz Curve Coefficient C:	0.0064(5)

Relevant parameters of $LiYO_2$-LT, 50992-ICSD

Structure and profile data:		Extinction:	0.000000
Formula sum: $Y_{3.88}Li_{4.00}O_{8.00}Yb_{0.08}Er_{0.04}$		Flat Plate Absorption Correction:	0.000000
Formula mass/ g/mol:	521.2477	Porosity:	0.000000
Density (calculated)/ g/cm^3	4.2046	Roughness:	0.000000
F(000):	235.6400	Fitting mode:	Structure Fit
Weight fraction/ %:	48.8(3)	U Left:	0.13(2)
Space group (No.):	P 1 21/c 1 (14)	V Left:	-0.12(1)
Lattice parameters:		W Left:	0.055(3)
a/ A:	6.1113(2)	Preferred orientation direction/ hkl:	0.00 0.00 1.00
b/ A:	6.2010(2)	Preferred orientation parameter:	1.000000
c/ A:	6.1854(2)	Asymmetry parameter 1:	0.81(3)
alpha/ °:	90	Asymmetry parameter 2:	0.000000
beta/ °:	118.585(3)	Peak shape:	
gamma/ °:	90	parameter 1 Left:	0.46(2)
V/ 10^6 pm^3	205.83070	parameter 2 Left:	0.000000
Overall displacement parameter:	0.000000	parameter 3 Left:	0.000000
		R (Bragg)/ %:	2.78252

Occupancy, atomic fract. coordinates and Biso for $LiYO_2$-LT, 50992-ICSD

Atom	Wyck.	s.o.f.	x	y	z	B/ 10^4 pm^2
Y1	4e	0.970000	0.266000	0.131200	0.020700	0.210000
Li1	4e	1.000000	0.791000	0.346000	0.068000	0.600000
O1	4e	1.000000	0.045900	0.107000	0.237800	0.280000
O2	4e	1.000000	0.512100	0.328800	0.343900	0.290000
Yb1	4e	0.020000	0.266000	0.131200	0.020700	0.210000
Er1	4e	0.010000	0.266000	0.131200	0.020700	0.210000

Relevant parameters of $LiYO_2$-HT, 50993-ICSD

Structure and profile data:		Extinction:	0.000000
Formula sum: $Y_{3.88}Li_{4.00}O_{8.00}Yb_{0.08}Er_{0.04}$		Flat Plate Absorption Correction:	0.000000
Formula mass/ g/mol:	521.2477	Porosity:	0.000000
Density (calculated)/ g/cm^3	4.2507	Roughness:	0.000000
F(000):	235.6400	Fitting mode:	Structure Fit
Weight fraction/ %:	47.4(3)	U Left:	0.072(9)
Space group (No.):	I 41/a m d (141)	V Left:	-0.038(7)
Lattice parameters:		W Left:	0.023(1)
a/ A:	4.44262(9)	Preferred orientation direction/ hkl:	0.00 0.00 1.00
b/ A:	4.44262(9)	Preferred orientation parameter:	1.000000
c/ A:	10.3155(2)	Asymmetry parameter 1:	0.000000
alpha/ °:	90	Asymmetry parameter 2:	0.000000
beta/ °:	90	Peak shape:	
gamma/ °:	90	parameter 1 Left:	0.59(2)
V/ 10^6 pm^3	203.59580	parameter 2 Left:	0.000000
Overall displacement parameter:	0.000000	parameter 3 Left:	0.000000
		R (Bragg)/ %:	3.68846

Occupancy, atomic fract. coordinates and Biso for $LiYO_2$-HT, 50993-ICSD

Atom	Wyck.	s.o.f.	x	y	z	B/ 10^4 pm^2
Y1	4b	0.970000	0.000000	0.250000	0.375000	0.000000
Li1	4b	0.000000	0.000000	0.250000	0.375000	0.000000
Li2	4a	1.000000	0.000000	0.750000	0.125000	0.000000
O1	8e	1.000000	0.000000	0.250000	0.152000	0.000000
Yb1	4b	0.020000	0.000000	0.250000	0.375000	0.000000
Er1	4b	0.010000	0.000000	0.250000	0.375000	0.000000

The data for the $LiYO_2$:2%Yb^{3+}, 1%Er^{3+} at 270 K:

Global Parameters

Number of used phases:	3	Asymmetry Type:	No Asymmetry Function
Number of variables:	26		
Number of constraints:	2	Asymmetry 1:	0.000000
Zero shift/ °2Theta:	0.000000	Asymmetry 2:	0.000000
Specimen displacement/ mm :	0.2234(9)	Shape Type:	Shape Individual
Profile function:	Pseudo Voigt	Shape 1 Left:	0.600000
Background:	Polynomial	Shape 2 Left:	0.000000
R (expected)/ %:	0.53407	Shape 3 Left:	0.000000
R (profile)/ %:	3.38320	Shape 1 Right:	0.600000
R (weighted profile)/ %:	4.65657	Shape 2 Right:	0.000000
GOF:	76.02027	Shape 3 Right:	0.000000
d-statistic:	0.25032	K a1/a2 intensity ratio:	0.500000
U standard:	0.000000	K alpha/beta intensity ratio:	0.000000
V standard:	0.000000	Crystal Shape Factor K:	1.0000
W standard:	0.010000	Instrumental FWHM Curve Type:	Caglioti function
U Left:	0.000000	Instr. Gauss Curve Coefficient A:	0.0045(5)
V Left:	0.000000	Instr. Gauss Curve Coefficient B:	-0.0032(9)
W Left:	0.010000	Instr. Gauss Curve Coefficient C:	0.0046(3)
U Right:	0.000000	Instr. Lorentz Curve Coefficient A:	0.0062(7)
V Right:	0.000000	Instr. Lorentz Curve Coefficient B:	-0.004(1)
W Right:	0.010000	Instr. Lorentz Curve Coefficient C:	0.0064(5)

Relevant parameters of $LiYO_2$-LT, 50992-ICSD

Structure and profile data:		Extinction:	0.000000
Formula sum: $Y_{3.88}Li_{4.00}O_{8.00}Yb_{0.08}Er_{0.04}$		Flat Plate Absorption Correction:	0.000000
Formula mass/ g/mol:	521.2477	Porosity:	0.000000
Density (calculated)/ g/cm^3	4.2066	Roughness:	0.000000
F(000):	235.6400	Fitting mode:	Structure Fit
Weight fraction/ %:	41.5(3)	U Left:	0.16(2)
Space group (No.):	P 1 21/c 1 (14)	V Left:	-0.15(1)
Lattice parameters:		W Left:	0.050(3)
a/ A:	6.1093(2)	Preferred orientation direction/ hkl:	0.00 0.00 1.00
b/ A:	6.2015(2)	Preferred orientation parameter:	1.000000
c/ A:	6.1833(2)	Asymmetry parameter 1:	1.30(4)
alpha/ °:	90	Asymmetry parameter 2:	0.000000
beta/ °:	118.574(3)	Peak shape:	
gamma/ °:	90	parameter 1 Left:	0.57(2)
V/ 10^6 pm^3	205.73340	parameter 2 Left:	0.000000
Overall displacement parameter:	0.000000	parameter 3 Left:	0.000000
		R (Bragg)/ %:	2.42768

Occupancy, atomic fract. coordinates and Biso for $LiYO_2$-LT, 50992-ICSD

Atom	Wyck.	s.o.f.	x	y	z	B/ 10^4 pm^2
Y1	4e	0.970000	0.266000	0.131200	0.020700	0.210000
Li1	4e	1.000000	0.791000	0.346000	0.068000	0.600000
O1	4e	1.000000	0.045900	0.107000	0.237800	0.280000
O2	4e	1.000000	0.512100	0.328800	0.343900	0.290000
Yb1	4e	0.020000	0.266000	0.131200	0.020700	0.210000
Er1	4e	0.010000	0.266000	0.131200	0.020700	0.210000

Relevant parameters of $LiYO_2$-HT, 50993-ICSD

Structure and profile data:		Extinction:	0.000000
Formula sum: $Y_{3.88}Li_{4.00}O_{8.00}Yb_{0.08}Er_{0.04}$		Flat Plate Absorption Correction:	0.000000
Formula mass/ g/mol:	521.2477	Porosity:	0.000000
Density (calculated)/ g/cm^3	4.2486	Roughness:	0.000000
F(000):	235.6400	Fitting mode:	Structure Fit
Weight fraction/ %:	53.8(3)	U Left:	0.040(6)
Space group (No.):	I 41/a m d (141)	V Left:	-0.011(5)
Lattice parameters:		W Left:	0.0146(9)
a/ A:	4.44255(8)	Preferred orientation direction/ hkl:	0.00 0.00 1.00
b/ A:	4.44255(8)	Preferred orientation parameter:	1.000000
c/ A:	10.3209(2)	Asymmetry parameter 1:	0.000000
alpha/ °:	90	Asymmetry parameter 2:	0.000000
beta/ °:	90	Peak shape:	
gamma/ °:	90	parameter 1 Left:	0.64(2)
V/ 10^6 pm^3	203.69640	parameter 2 Left:	0.000000
Overall displacement parameter:	0.000000	parameter 3 Left:	0.000000
		R (Bragg)/ %:	3.48426

Occupancy, atomic fract. coordinates and Biso for $LiYO_2$-HT, 50993-ICSD

Atom	Wyck.	s.o.f.	x	y	z	B/ 10^4 pm^2
Y1	4b	0.970000	0.000000	0.250000	0.375000	0.000000
Li1	4b	0.000000	0.000000	0.250000	0.375000	0.000000
Li2	4a	1.000000	0.000000	0.750000	0.125000	0.000000
O1	8e	1.000000	0.000000	0.250000	0.152000	0.000000
Yb1	4b	0.020000	0.000000	0.250000	0.375000	0.000000
Er1	4b	0.010000	0.000000	0.250000	0.375000	0.000000

The data for the $LiYO_2$:2%Yb^{3+}, 1%Er^{3+} at 280 K:

Global Parameters

Number of used phases:	3	Asymmetry Type:	No Asymmetry Function
Number of variables:	21		
Number of constraints:	2	Asymmetry 1:	0.000000
Zero shift/ °2Theta:	0.000000	Asymmetry 2:	0.000000
Specimen displacement/ mm :	0.1832(9)	Shape Type:	Shape Individual
Profile function:	Pseudo Voigt	Shape 1 Left:	0.600000
Background:	Polynomial	Shape 2 Left:	0.000000
R (expected)/ %:	0.54465	Shape 3 Left:	0.000000
R (profile)/ %:	3.44187	Shape 1 Right:	0.600000
R (weighted profile)/ %:	4.78748	Shape 2 Right:	0.000000
GOF:	77.26488	Shape 3 Right:	0.000000
d-statistic:	0.21525	K a1/a2 intensity ratio:	0.500000
U standard:	0.000000	K alpha/beta intensity ratio:	0.000000
V standard:	0.000000	Crystal Shape Factor K:	1.0000
W standard:	0.010000	Instrumental FWHM Curve Type:	Caglioti function
U Left:	0.000000	Instr. Gauss Curve Coefficient A:	0.0045(5)
V Left:	0.000000	Instr. Gauss Curve Coefficient B:	-0.0032(9)
W Left:	0.010000	Instr. Gauss Curve Coefficient C:	0.0046(3)
U Right:	0.000000	Instr. Lorentz Curve Coefficient A:	0.0062(7)
V Right:	0.000000	Instr. Lorentz Curve Coefficient B:	-0.004(1)
W Right:	0.010000	Instr. Lorentz Curve Coefficient C:	0.0064(5)

Relevant parameters of $LiYO_2$-LT, 50992-ICSD

Structure and profile data:		Extinction:	0.000000
Formula sum: $Y_{3.88}Li_{4.00}O_{8.00}Yb_{0.08}Er_{0.04}$		Flat Plate Absorption Correction:	0.000000
Formula mass/ g/mol:	521.2477	Porosity:	0.000000
Density (calculated)/ g/cm^3	4.2017	Roughness:	0.000000
F(000):	235.6400	Fitting mode:	Structure Fit
Weight fraction/ %:	19.9(2)	U Left:	0.164211
Space group (No.):	P 1 21/c 1 (14)	V Left:	-0.149267
Lattice parameters:		W Left:	0.049318
a/ A:	6.1118(4)	Preferred orientation direction/ hkl:	0.00 0.00 1.00
b/ A:	6.2069(5)	Preferred orientation parameter:	1.000000
c/ A:	6.1804(5)	Asymmetry parameter 1:	1.038700
alpha/ °:	90	Asymmetry parameter 2:	0.000000
beta/ °:	118.536(6)	Peak shape:	
gamma/ °:	90	parameter 1 Left:	0.643958
V/ 10^6 pm^3	205.97190	parameter 2 Left:	0.000000
Overall displacement parameter:	0.000000	parameter 3 Left:	0.000000
		R (Bragg)/ %:	2.61948

Occupancy, atomic fract. coordinates and Biso for $LiYO_2$-LT, 50992-ICSD

Atom	Wyck.	s.o.f.	x	y	z	B/ 10^4 pm^2
Y1	4e	0.970000	0.266000	0.131200	0.020700	0.210000
Li1	4e	1.000000	0.791000	0.346000	0.068000	0.600000
O1	4e	1.000000	0.045900	0.107000	0.237800	0.280000
O2	4e	1.000000	0.512100	0.328800	0.343900	0.290000
Yb1	4e	0.020000	0.266000	0.131200	0.020700	0.210000
Er1	4e	0.010000	0.266000	0.131200	0.020700	0.210000

Relevant parameters of $LiYO_2$-HT, 50993-ICSD

Structure and profile data:		a/ A:	4.44246(7)
Formula sum: $Y_{3.88}Li_{4.00}O_{8.00}Yb_{0.08}Er_{0.04}$		b/ A:	4.44246(7)
Formula mass/ g/mol:	521.2477	c/ A:	10.3248(2)
Density (calculated)/ g/cm^3	4.2472	alpha/ °:	90
F(000):	235.6400	beta/ °:	90
Weight fraction/ %:	75.9(3)	gamma/ °:	90
Space group (No.):	I 41/a m d (141)	V/ 10^6 pm^3	203.76500
Lattice parameters:		Overall displacement parameter:	0.000000
		Extinction:	0.000000

Flat Plate Absorption Correction:	0.000000	Preferred orientation parameter:	1.000000
Porosity:	0.000000	Asymmetry parameter 1:	0.000000
Roughness:	0.000000	Asymmetry parameter 2:	0.000000
Fitting mode:	Structure Fit	Peak shape:	
U Left:	0.047(5)	parameter 1 Left:	0.55(1)
V Left:	-0.023(4)	parameter 2 Left:	0.000000
W Left:	0.0216(8)	parameter 3 Left:	0.000000
Preferred orientation direction/ hkl:	0.00 0.00 1.00	R (Bragg)/ %:	3.61651

Occupancy, atomic fract. coordinates and Biso for LiYO$_2$-HT, 50993-ICSD

Atom	Wyck.	s.o.f.	x	y	z	B/ 10^4 pm^2
Y1	4b	0.970000	0.000000	0.250000	0.375000	0.000000
Li1	4b	0.000000	0.000000	0.250000	0.375000	0.000000
Li2	4a	1.000000	0.000000	0.750000	0.125000	0.000000
O1	8e	1.000000	0.000000	0.250000	0.152000	0.000000
Yb1	4b	0.020000	0.000000	0.250000	0.375000	0.000000
Er1	4b	0.010000	0.000000	0.250000	0.375000	0.000000

The data for the LiYO$_2$:2%Yb^{3+}, 1%Er^{3+} at 290 K:

Global Parameters

Number of used phases:	3	Asymmetry Type:	No Asymmetry Function
Number of variables:	18		
Number of constraints:	2	Asymmetry 1:	0.000000
Zero shift/ °2Theta:	0.000000	Asymmetry 2:	0.000000
Specimen displacement/ mm :	0.2117(8)	Shape Type:	Shape Individual
Profile function:	Pseudo Voigt	Shape 1 Left:	0.600000
Background:	Polynomial	Shape 2 Left:	0.000000
R (expected)/ %:	0.53469	Shape 3 Left:	0.000000
R (profile)/ %:	3.85841	Shape 1 Right:	0.600000
R (weighted profile)/ %:	5.54071	Shape 2 Right:	0.000000
GOF:	107.38270	Shape 3 Right:	0.000000
d-statistic:	0.14068	K a1/a2 intensity ratio:	0.500000
U standard:	0.000000	K alpha/beta intensity ratio:	0.000000
V standard:	0.000000	Crystal Shape Factor K:	1.0000
W standard:	0.010000	Instrumental FWHM Curve Type:	Caglioti function
U Left:	0.000000	Instr. Gauss Curve Coefficient A:	0.0045(5)
V Left:	0.000000	Instr. Gauss Curve Coefficient B:	-0.0032(9)
W Left:	0.010000	Instr. Gauss Curve Coefficient C:	0.0046(3)
U Right:	0.000000	Instr. Lorentz Curve Coefficient A:	0.0062(7)
V Right:	0.000000	Instr. Lorentz Curve Coefficient B:	-0.004(1)
W Right:	0.010000	Instr. Lorentz Curve Coefficient C:	0.0064(5)

Relevant parameters of LiYO$_2$-LT, 50992-ICSD

Structure and profile data:		Extinction:	0.000000
Formula sum: $Y_{3.88}Li_{4.00}O_{8.00}Yb_{0.08}Er_{0.04}$		Flat Plate Absorption Correction:	0.000000
Formula mass/ g/mol:	521.2477	Porosity:	0.000000
Density (calculated)/ g/cm^3	4.2018	Roughness:	0.000000
F(000):	235.6400	Fitting mode:	Structure Fit
Weight fraction/ %:	7.4(2)	U Left:	0.164211
Space group (No.):	P 1 21/c 1 (14)	V Left:	-0.149267
Lattice parameters:		W Left:	0.049318
a/ A:	6.111779	Preferred orientation direction/ hkl:	0.00 0.00 1.00
b/ A:	6.206623	Preferred orientation parameter:	1.000000
c/ A:	6.180401	Asymmetry parameter 1:	1.038700
alpha/ °:	90	Asymmetry parameter 2:	0.000000
beta/ °:	118.535600	Peak shape:	
gamma/ °:	90	parameter 1 Left:	0.643958
V/ 10^6 pm^3	205.96410	parameter 2 Left:	0.000000
Overall displacement parameter:	0.000000	parameter 3 Left:	0.000000
		R (Bragg)/ %:	3.09385

Occupancy, atomic fract. coordinates and Biso for LiYO$_2$-LT, 50992-ICSD

Atom	Wyck.	s.o.f.	x	y	z	B/ 10^4 pm^2
Y1	4e	0.970000	0.266000	0.131200	0.020700	0.210000
Li1	4e	1.000000	0.791000	0.346000	0.068000	0.600000
O1	4e	1.000000	0.045900	0.107000	0.237800	0.280000
O2	4e	1.000000	0.512100	0.328800	0.343900	0.290000
Yb1	4e	0.020000	0.266000	0.131200	0.020700	0.210000
Er1	4e	0.010000	0.266000	0.131200	0.020700	0.210000

Relevant parameters of LiYO$_2$-HT, 50993-ICSD

Structure and profile data:		Extinction:	0.000000
Formula sum: $Y_{3.88}Li_{4.00}O_{8.00}Yb_{0.08}Er_{0.04}$		Flat Plate Absorption Correction:	0.000000
Formula mass/ g/mol:	521.2477	Porosity:	0.000000
Density (calculated)/ g/cm^3	4.2457	Roughness:	0.000000
F(000):	235.6400	Fitting mode:	Structure Fit
Weight fraction/ %:	87.5(3)	U Left:	0.034(4)
Space group (No.):	I 41/a m d (141)	V Left:	-0.011(4)
Lattice parameters:		W Left:	0.0155(7)
a/ A:	4.44169(6)	Preferred orientation direction/ hkl:	0.00 0.00 1.00
b/ A:	4.44169(6)	Preferred orientation parameter:	1.000000
c/ A:	10.3320(2)	Asymmetry parameter 1:	0.58(2)
alpha/ °:	90	Asymmetry parameter 2:	0.000000
beta/ °:	90	Peak shape:	
gamma/ °:	90	parameter 1 Left:	0.59(1)
V/ 10^6 pm^3	203.83530	parameter 2 Left:	0.000000
Overall displacement parameter:	0.000000	parameter 3 Left:	0.000000
		R (Bragg)/ %:	4.53603

Occupancy, atomic fract. coordinates and Biso for $LiYO_2$-HT, 50993-ICSD

Atom	Wyck.	s.o.f.	x	y	z	B/ 10^4 pm^2
Y1	4b	0.970000	0.000000	0.250000	0.375000	0.000000
Li1	4b	0.000000	0.000000	0.250000	0.375000	0.000000
Li2	4a	1.000000	0.000000	0.750000	0.125000	0.000000
O1	8e	1.000000	0.000000	0.250000	0.152000	0.000000
Yb1	4b	0.020000	0.000000	0.250000	0.375000	0.000000
Er1	4b	0.010000	0.000000	0.250000	0.375000	0.000000

The data for the $LiYO_2$:2%Yb^{3+}, 1%Er^{3+} at 300 K:

Global Parameters

Number of used phases:	3	Asymmetry Type:	No Asymmetry Function
Number of variables:	17		
Number of constraints:	2	Asymmetry 1:	0.000000
Zero shift/ °2Theta:	0.000000	Asymmetry 2:	0.000000
Specimen displacement/ mm :	0.2029(8)	Shape Type:	Shape Individual
Profile function:	Pseudo Voigt	Shape 1 Left:	0.600000
Background:	Polynomial	Shape 2 Left:	0.000000
R (expected)/ %:	0.53409	Shape 3 Left:	0.000000
R (profile)/ %:	4.02742	Shape 1 Right:	0.600000
R (weighted profile)/ %:	5.85502	Shape 2 Right:	0.000000
GOF:	120.17870	Shape 3 Right:	0.000000
d-statistic:	0.12683	K a1/a2 intensity ratio:	0.500000
U standard:	0.000000	K alpha/beta intensity ratio:	0.000000
V standard:	0.000000	Crystal Shape Factor K:	1.0000
W standard:	0.010000	Instrumental FWHM Curve Type:	Caglioti function
U Left:	0.000000	Instr. Gauss Curve Coefficient A:	0.0045(5)
V Left:	0.000000	Instr. Gauss Curve Coefficient B:	-0.0032(9)
W Left:	0.010000	Instr. Gauss Curve Coefficient C:	0.0046(3)
U Right:	0.000000	Instr. Lorentz Curve Coefficient A:	0.0062(7)
V Right:	0.000000	Instr. Lorentz Curve Coefficient B:	-0.004(1)
W Right:	0.010000	Instr. Lorentz Curve Coefficient C:	0.0064(5)

Relevant parameters of $LiYO_2$-LT, 50992-ICSD

Structure and profile data:		Flat Plate Absorption Correction:	0.000000
Formula sum: $Y_{3.88}Li_{4.00}O_{8.00}Yb_{0.08}Er_{0.04}$		Porosity:	0.000000
Formula mass/ g/mol:	521.2477	Roughness:	0.000000
Density (calculated)/ g/cm^3	4.2018	Fitting mode:	Structure Fit
F(000):	235.6400	U Left:	0.164211
Weight fraction/ %:	0.000000	V Left:	-0.149267
Space group (No.):	P 1 21/c 1 (14)	W Left:	0.049318
Lattice parameters:		Preferred orientation direction/ hkl:	0.00 0.00 1.00
a/ A:	6.111779	Preferred orientation parameter:	1.000000
b/ A:	6.206623	Asymmetry parameter 1:	1.038700
c/ A:	6.180401	Asymmetry parameter 2:	0.000000
alpha/ °:	90	Peak shape:	
beta/ °:	118.535600	parameter 1 Left:	0.643958
gamma/ °:	90	parameter 2 Left:	0.000000
V/ 10^6 pm^3	205.96410	parameter 3 Left:	0.000000
Overall displacement parameter:	0.000000	R (Bragg)/ %:	-2.98867
Extinction:	0.000000		

Occupancy, atomic fract. coordinates and Biso for $LiYO_2$-LT, 50992-ICSD

Atom	Wyck.	s.o.f.	x	y	z	B/ 10^4 pm^2
Y1	4e	0.970000	0.266000	0.131200	0.020700	0.210000
Li1	4e	1.000000	0.791000	0.346000	0.068000	0.600000
O1	4e	1.000000	0.045900	0.107000	0.237800	0.280000
O2	4e	1.000000	0.512100	0.328800	0.343900	0.290000
Yb1	4e	0.020000	0.266000	0.131200	0.020700	0.210000
Er1	4e	0.010000	0.266000	0.131200	0.020700	0.210000

Relevant parameters of $LiYO_2$-HT, 50993-ICSD

Structure and profile data:	
Formula sum: $Y_{3.88}Li_{4.00}O_{8.00}Yb_{0.08}Er_{0.04}$	
Formula mass/ g/mol:	521.2477
Density (calculated)/ g/cm^3	4.2443
F(000):	235.6400
Weight fraction/ %:	95.3(3)
Space group (No.):	I 41/a m d (141)
Lattice parameters:	
a/ A:	4.44221(6)
b/ A:	4.44221(6)
c/ A:	10.3330(2)
alpha/ °:	90
beta/ °:	90
gamma/ °:	90
V/ 10^6 pm^3	203.90350
Overall displacement parameter:	0.000000
Extinction:	0.000000
Flat Plate Absorption Correction:	0.000000
Porosity:	0.000000
Roughness:	0.000000
Fitting mode:	Structure Fit
U Left:	0.034(4)
V Left:	-0.009(4)
W Left:	0.0153(7)
Preferred orientation direction/ hkl:	0.00 0.00 1.00
Preferred orientation parameter:	1.000000
Asymmetry parameter 1:	0.74(2)
Asymmetry parameter 2:	0.000000
Peak shape:	
parameter 1 Left:	0.593(9)
parameter 2 Left:	0.000000
parameter 3 Left:	0.000000
R (Bragg)/ %:	4.83555

Occupancy, atomic fract. coordinates and Biso for $LiYO_2$-HT, 50993-ICSD

Atom	Wyck.	s.o.f.	x	y	z	B/ 10^4 pm^2
Y1	4b	0.970000	0.000000	0.250000	0.375000	0.000000
Li1	4b	0.000000	0.000000	0.250000	0.375000	0.000000
Li2	4a	1.000000	0.000000	0.750000	0.125000	0.000000
O1	8e	1.000000	0.000000	0.250000	0.152000	0.000000
Yb1	4b	0.020000	0.000000	0.250000	0.375000	0.000000
Er1	4b	0.010000	0.000000	0.250000	0.375000	0.000000

The data for the $LiYO_2$:2%Yb^{3+}, 1%Er^{3+} at 310 K:

Global Parameters

Number of used phases:	3
Number of variables:	17
Number of constraints:	2
Zero shift/ °2Theta:	0.000000
Specimen displacement/ mm :	0.1952(8)
Profile function:	Pseudo Voigt
Background:	Polynomial
R (expected)/ %:	0.53246
R (profile)/ %:	3.98129
R (weighted profile)/ %:	5.80745
GOF:	118.95880
d-statistic:	0.12454
U standard:	0.000000
V standard:	0.000000
W standard:	0.010000
U Left:	0.000000
V Left:	0.000000
W Left:	0.010000
U Right:	0.000000
V Right:	0.000000
W Right:	0.010000
Asymmetry Type:	No Asymmetry Function
Asymmetry 1:	0.000000
Asymmetry 2:	0.000000
Shape Type:	Shape Individual
Shape 1 Left:	0.600000
Shape 2 Left:	0.000000
Shape 3 Left:	0.000000
Shape 1 Right:	0.600000
Shape 2 Right:	0.000000
Shape 3 Right:	0.000000
K a1/a2 intensity ratio:	0.500000
K alpha/beta intensity ratio:	0.000000
Crystal Shape Factor K:	1.0000
Instrumental FWHM Curve Type:	Caglioti function
Instr. Gauss Curve Coefficient A:	0.0045(5)
Instr. Gauss Curve Coefficient B:	-0.0032(9)
Instr. Gauss Curve Coefficient C:	0.0046(3)
Instr. Lorentz Curve Coefficient A:	0.0062(7)
Instr. Lorentz Curve Coefficient B:	-0.004(1)
Instr. Lorentz Curve Coefficient C:	0.0064(5)

Relevant parameters of $LiYO_2$-LT, 50992-ICSD

Structure and profile data:	
Formula sum: $Y_{3.88}Li_{4.00}O_{8.00}Yb_{0.08}Er_{0.04}$	
Formula mass/ g/mol:	521.2477
Density (calculated)/ g/cm^3	4.2018
F(000):	235.6400
Weight fraction/ %:	0.000000
Space group (No.):	P 1 21/c 1 (14)
Lattice parameters:	
a/ A:	6.111779
b/ A:	6.206623
c/ A:	6.180401
alpha/ °:	90

beta/ °:	118.535600	V Left:	-0.149267
gamma/ °:	90	W Left:	0.049318
V/ 10^6 pm^3	205.96410	Preferred orientation direction/ hkl:	0.00 0.00 1.00
Overall displacement parameter:	0.000000	Preferred orientation parameter:	1.000000
Extinction:	0.000000	Asymmetry parameter 1:	1.038700
Flat Plate Absorption Correction:	0.000000	Asymmetry parameter 2:	0.000000
Porosity:	0.000000	Peak shape:	
Roughness:	0.000000	parameter 1 Left:	0.643958
Fitting mode:	Structure Fit	parameter 2 Left:	0.000000
U Left:	0.164211	parameter 3 Left:	0.000000
		R (Bragg)/ %:	-2.77731

Occupancy, atomic fract. coordinates and Biso for $LiYO_2$-LT, 50992-ICSD

Atom	Wyck.	s.o.f.	x	y	z	B/ 10^4 pm^2
Y1	4e	0.970000	0.266000	0.131200	0.020700	0.210000
Li1	4e	1.000000	0.791000	0.346000	0.068000	0.600000
O1	4e	1.000000	0.045900	0.107000	0.237800	0.280000
O2	4e	1.000000	0.512100	0.328800	0.343900	0.290000
Yb1	4e	0.020000	0.266000	0.131200	0.020700	0.210000
Er1	4e	0.010000	0.266000	0.131200	0.020700	0.210000

Relevant parameters of $LiYO_2$-HT, 50993-ICSD

Structure and profile data:		Extinction:	0.000000
Formula sum: $Y_{3.88}Li_{4.00}O_{8.00}Yb_{0.08}Er_{0.04}$		Flat Plate Absorption Correction:	0.000000
Formula mass/ g/mol:	521.2477	Porosity:	0.000000
Density (calculated)/ g/cm^3	4.2432	Roughness:	0.000000
F(000):	235.6400	Fitting mode:	Structure Fit
Weight fraction/ %:	95.0(3)	U Left:	0.042(5)
Space group (No.):	I 41/a m d (141)	V Left:	-0.019(4)
Lattice parameters:		W Left:	0.0185(7)
a/ A:	4.44272(6)	Preferred orientation direction/ hkl:	0.00 0.00 1.00
b/ A:	4.44272(6)	Preferred orientation parameter:	1.000000
c/ A:	10.3334(2)	Asymmetry parameter 1:	0.73(2)
alpha/ °:	90	Asymmetry parameter 2:	0.000000
beta/ °:	90	Peak shape:	
gamma/ °:	90	parameter 1 Left:	0.565(9)
V/ 10^6 pm^3	203.95750	parameter 2 Left:	0.000000
Overall displacement parameter:	0.000000	parameter 3 Left:	0.000000
		R (Bragg)/ %:	4.44152

Occupancy, atomic fract. coordinates and Biso for $LiYO_2$-HT, 50993-ICSD

Atom	Wyck.	s.o.f.	x	y	z	B/ 10^4 pm^2
Y1	4b	0.970000	0.000000	0.250000	0.375000	0.000000
Li1	4b	0.000000	0.000000	0.250000	0.375000	0.000000
Li2	4a	1.000000	0.000000	0.750000	0.125000	0.000000
O1	8e	1.000000	0.000000	0.250000	0.152000	0.000000
Yb1	4b	0.020000	0.000000	0.250000	0.375000	0.000000
Er1	4b	0.010000	0.000000	0.250000	0.375000	0.000000

The data for the $LiYO_2$:2%Yb^{3+}, 1%Er^{3+} at 320 K:

Global Parameters

Number of used phases:	3	V Right:	0.000000
Number of variables:	17	W Right:	0.010000
Number of constraints:	2	Asymmetry Type:	No Asymmetry Function
Zero shift/ °2Theta:	0.000000		
Specimen displacement/ mm :	0.1898(8)	Asymmetry 1:	0.000000
Profile function:	Pseudo Voigt	Asymmetry 2:	0.000000
Background:	Polynomial	Shape Type:	Shape Individual
R (expected)/ %:	0.53092	Shape 1 Left:	0.600000
R (profile)/ %:	4.18934	Shape 2 Left:	0.000000
R (weighted profile)/ %:	6.09113	Shape 3 Left:	0.000000
GOF:	131.62500	Shape 1 Right:	0.600000
d-statistic:	0.11421	Shape 2 Right:	0.000000
U standard:	0.000000	Shape 3 Right:	0.000000
V standard:	0.000000	K a1/a2 intensity ratio:	0.500000
W standard:	0.010000	K alpha/beta intensity ratio:	0.000000
U Left:	0.000000	Crystal Shape Factor K:	1.0000
V Left:	0.000000	Instrumental FWHM Curve Type:	Caglioti function
W Left:	0.010000	Instr. Gauss Curve Coefficient A:	0.0045(5)
U Right:	0.000000	Instr. Gauss Curve Coefficient B:	-0.0032(9)

Instr. Gauss Curve Coefficient C:	0.0046(3)	Instr. Lorentz Curve Coefficient B:	-0.004(1)
Instr. Lorentz Curve Coefficient A:	0.0062(7)	Instr. Lorentz Curve Coefficient C:	0.0064(5)

Relevant parameters of $LiYO_2$-LT, 50992-ICSD

Structure and profile data:		Extinction:	0.000000
Formula sum: $Y_{3.88}Li_{4.00}O_{8.00}Yb_{0.08}Er_{0.04}$		Flat Plate Absorption Correction:	0.000000
Formula mass/ g/mol:	521.2477	Porosity:	0.000000
Density (calculated)/ g/cm^3	4.2018	Roughness:	0.000000
F(000):	235.6400	Fitting mode:	Structure Fit
Weight fraction/ %:	0.000000	U Left:	0.164211
Space group (No.):	P 1 21/c 1 (14)	V Left:	-0.149267
Lattice parameters:		W Left:	0.049318
a/ A:	6.111779	Preferred orientation direction/ hkl:	0.00 0.00 1.00
b/ A:	6.206623	Preferred orientation parameter:	1.000000
c/ A:	6.180401	Asymmetry parameter 1:	1.038700
alpha/ °:	90	Asymmetry parameter 2:	0.000000
beta/ °:	118.535600	Peak shape:	
gamma/ °:	90	parameter 1 Left:	0.643958
V/ 10^6 pm^3	205.96410	parameter 2 Left:	0.000000
Overall displacement parameter:	0.000000	parameter 3 Left:	0.000000
		R (Bragg)/ %:	-2.73396

Occupancy, atomic fract. coordinates and Biso for $LiYO_2$-LT, 50992-ICSD

Atom	Wyck.	s.o.f.	x	y	z	B/ 10^4 pm^2
Y1	4e	0.970000	0.266000	0.131200	0.020700	0.210000
Li1	4e	1.000000	0.791000	0.346000	0.068000	0.600000
O1	4e	1.000000	0.045900	0.107000	0.237800	0.280000
O2	4e	1.000000	0.512100	0.328800	0.343900	0.290000
Yb1	4e	0.020000	0.266000	0.131200	0.020700	0.210000
Er1	4e	0.010000	0.266000	0.131200	0.020700	0.210000

Relevant parameters of $LiYO_2$-HT, 50993-ICSD

Structure and profile data:		Extinction:	0.000000
Formula sum: $Y_{3.88}Li_{4.00}O_{8.00}Yb_{0.08}Er_{0.04}$		Flat Plate Absorption Correction:	0.000000
Formula mass/ g/mol:	521.2477	Porosity:	0.000000
Density (calculated)/ g/cm^3	4.2421	Roughness:	0.000000
F(000):	235.6400	Fitting mode:	Structure Fit
Weight fraction/ %:	94.7(3)	U Left:	0.044(4)
Space group (No.):	I 41/a m d (141)	V Left:	-0.026(4)
Lattice parameters:		W Left:	0.0207(7)
a/ A:	4.44334(6)	Preferred orientation direction/ hkl:	0.00 0.00 1.00
b/ A:	4.44334(6)	Preferred orientation parameter:	1.000000
c/ A:	10.3331(2)	Asymmetry parameter 1:	0.73(2)
alpha/ °:	90	Asymmetry parameter 2:	0.000000
beta/ °:	90	Peak shape:	
gamma/ °:	90	parameter 1 Left:	0.563(9)
V/ 10^6 pm^3	204.00850	parameter 2 Left:	0.000000
Overall displacement parameter:	0.000000	parameter 3 Left:	0.000000
		R (Bragg)/ %:	4.57826

Occupancy, atomic fract. coordinates and Biso for $LiYO_2$-HT, 50993-ICSD

Atom	Wyck.	s.o.f.	x	y	z	B/ 10^4 pm^2
Y1	4b	0.970000	0.000000	0.250000	0.375000	0.000000
Li1	4b	0.000000	0.000000	0.250000	0.375000	0.000000
Li2	4a	1.000000	0.000000	0.750000	0.125000	0.000000
O1	8e	1.000000	0.000000	0.250000	0.152000	0.000000
Yb1	4b	0.020000	0.000000	0.250000	0.375000	0.000000
Er1	4b	0.010000	0.000000	0.250000	0.375000	0.000000

The data for the $LiYO_2$:5%Yb^{3+}, 1%Er^{3+} at 100 K:

Global Parameters

Number of used phases:	3	GOF:	81.13222
Number of variables:	20	d-statistic:	0.16364
Number of constraints:	2	U standard:	0.000000
Zero shift/ °2Theta:	0.000000	V standard:	0.000000
Specimen displacement/ mm :	0.274(1)	W standard:	0.010000
Profile function:	Pseudo Voigt	U Left:	0.000000
Background:	Polynomial	V Left:	0.000000
R (expected)/ %:	0.59279	W Left:	0.010000
R (profile)/ %:	3.67845	U Right:	0.000000
R (weighted profile)/ %:	5.33944	V Right:	0.000000

W Right:	0.010000	K a1/a2 intensity ratio:	0.500000
Asymmetry Type:	No Asymmetry Function	K alpha/beta intensity ratio:	0.000000
		Crystal Shape Factor K:	1.0000
Asymmetry 1:	0.000000	Instrumental FWHM Curve Type:	Caglioti function
Asymmetry 2:	0.000000	Instr. Gauss Curve Coefficient A:	0.0045(5)
Shape Type:	Shape Individual	Instr. Gauss Curve Coefficient B:	-0.0032(9)
Shape 1 Left:	0.600000	Instr. Gauss Curve Coefficient C:	0.0046(3)
Shape 2 Left:	0.000000	Instr. Lorentz Curve Coefficient A:	0.0062(7)
Shape 3 Left:	0.000000	Instr. Lorentz Curve Coefficient B:	-0.004(1)
Shape 1 Right:	0.600000	Instr. Lorentz Curve Coefficient C:	0.0064(5)
Shape 2 Right:	0.000000		
Shape 3 Right:	0.000000		

Relevant parameters of $LiYO_2$-LT, 50992-ICSD

Structure and profile data:

Formula sum: $Y_{3.76}Li_{4.00}O_{8.00}Yb_{0.20}Er_{0.04}$

Formula mass/ g/mol:	531.3438	Flat Plate Absorption Correction:	0.000000
Density (calculated)/ g/cm^3	4.2883	Porosity:	0.000000
F(000):	239.3600	Roughness:	0.000000
Weight fraction/ %:	87.3(3)	Fitting mode:	Structure Fit
Space group (No.):	P 1 21/c 1 (14)	U Left:	0.04(1)
Lattice parameters:		V Left:	-0.006(7)
a/ A:	6.1229(2)	W Left:	0.017(1)
b/ A:	6.1746(2)	Preferred orientation direction/ hkl:	0.00 0.00 1.00
c/ A:	6.2104(2)	Preferred orientation parameter:	1.000000
alpha/ °:	90	Asymmetry parameter 1:	0.06(2)
beta/ °:	118.816(2)	Asymmetry parameter 2:	0.000000
gamma/ °:	90	Peak shape:	
V/ 10^6 pm^3	205.71970	parameter 1 Left:	0.55(1)
Overall displacement parameter:	0.000000	parameter 2 Left:	0.000000
Extinction:	0.000000	parameter 3 Left:	0.000000
		R (Bragg)/ %:	4.95542

Occupancy, atomic fract. coordinates and Biso for $LiYO_2$-LT, 50992-ICSD

Atom	Wyck.	s.o.f.	x	y	z	B/ 10^4 pm^2
Y1	4e	0.940000	0.266000	0.131200	0.020700	0.210000
Li1	4e	1.000000	0.791000	0.346000	0.068000	0.600000
O1	4e	1.000000	0.045900	0.107000	0.237800	0.280000
O2	4e	1.000000	0.512100	0.328800	0.343900	0.290000
Yb1	4e	0.050000	0.266000	0.131200	0.020700	0.210000
Er1	4e	0.010000	0.266000	0.131200	0.020700	0.210000

Relevant parameters of $LiYO_2$-HT, 50993-ICSD

Structure and profile data:

Formula sum: $Y_{3.76}Li_{4.00}O_{8.00}Yb_{0.20}Er_{0.04}$

Formula mass/ g/mol:	531.3438	Flat Plate Absorption Correction:	0.000000
Density (calculated)/ g/cm^3	4.3539	Porosity:	0.000000
F(000):	239.3600	Roughness:	0.000000
Weight fraction/ %:	0.000000	Fitting mode:	Structure Fit
Space group (No.):	I 41/a m d (141)	U Left:	0.068937
Lattice parameters:		V Left:	-0.052995
a/ A: 4(393617702912)		W Left:	0.027085
b/ A: 4(393617702912)		Preferred orientation direction/ hkl:	0.00 0.00 1.00
c/ A: 10(4904589983744)		Preferred orientation parameter:	1.000000
alpha/ °:	90	Asymmetry parameter 1:	0.170069
beta/ °:	90	Asymmetry parameter 2:	0.000000
gamma/ °:	90	Peak shape:	
V/ 10^6 pm^3	202.62080	parameter 1 Left:	0.626711
Overall displacement parameter:	0.000000	parameter 2 Left:	0.000000
Extinction:	0.000000	parameter 3 Left:	0.000000
		R (Bragg)/ %:	999.00000

Occupancy, atomic fract. coordinates and Biso for $LiYO_2$-HT, 50993-ICSD

Atom	Wyck.	s.o.f.	x	y	z	B/ 10^4 pm^2
Y1	4b	0.940000	0.000000	0.250000	0.375000	0.000000
Li1	4b	0.000000	0.000000	0.250000	0.375000	0.000000
Li2	4a	1.000000	0.000000	0.750000	0.125000	0.000000
O1	8e	1.000000	0.000000	0.250000	0.152000	0.000000
Yb1	4b	0.050000	0.000000	0.250000	0.375000	0.000000
Er1	4b	0.010000	0.000000	0.250000	0.375000	0.000000

The data for the $LiYO_2$:5%Yb^{3+}, 1%Er^{3+} at 120 K:

Global Parameters

Number of used phases:	3	Asymmetry Type:	No Asymmetry Function
Number of variables:	20		
Number of constraints:	2	Asymmetry 1:	0.000000
Zero shift/ °2Theta:	0.000000	Asymmetry 2:	0.000000
Specimen displacement/ mm :	0.270(1)	Shape Type:	Shape Individual
Profile function:	Pseudo Voigt	Shape 1 Left:	0.600000
Background:	Polynomial	Shape 2 Left:	0.000000
R (expected)/ %:	0.59355	Shape 3 Left:	0.000000
R (profile)/ %:	3.67440	Shape 1 Right:	0.600000
R (weighted profile)/ %:	5.32039	Shape 2 Right:	0.000000
GOF:	80.34673	Shape 3 Right:	0.000000
d-statistic:	0.16693	K a1/a2 intensity ratio:	0.500000
U standard:	0.000000	K alpha/beta intensity ratio:	0.000000
V standard:	0.000000	Crystal Shape Factor K:	1.0000
W standard:	0.010000	Instrumental FWHM Curve Type:	Caglioti function
U Left:	0.000000	Instr. Gauss Curve Coefficient A:	0.0045(5)
V Left:	0.000000	Instr. Gauss Curve Coefficient B:	-0.0032(9)
W Left:	0.010000	Instr. Gauss Curve Coefficient C:	0.0046(3)
U Right:	0.000000	Instr. Lorentz Curve Coefficient A:	0.0062(7)
V Right:	0.000000	Instr. Lorentz Curve Coefficient B:	-0.004(1)
W Right:	0.010000	Instr. Lorentz Curve Coefficient C:	0.0064(5)

Relevant parameters of $LiYO_2$-LT, 50992-ICSD

Structure and profile data:		Extinction:	0.000000
Formula sum: $Y_{3.76}Li_{4.00}O_{8.00}Yb_{0.20}Er_{0.04}$		Flat Plate Absorption Correction:	0.000000
Formula mass/ g/mol:	531.3438	Porosity:	0.000000
Density (calculated)/ g/cm^3	4.2889	Roughness:	0.000000
F(000):	239.3600	Fitting mode:	Structure Fit
Weight fraction/ %:	87.2(3)	U Left:	0.04(1)
Space group (No.):	P 1 21/c 1 (14)	V Left:	-0.007(7)
Lattice parameters:		W Left:	0.018(1)
a/ A:	6.1212(2)	Preferred orientation direction/ hkl:	0.00 0.00 1.00
b/ A:	6.1762(2)	Preferred orientation parameter:	1.000000
c/ A:	6.2082(2)	Asymmetry parameter 1:	0.07(2)
alpha/ °:	90	Asymmetry parameter 2:	0.000000
beta/ °:	118.789(2)	Peak shape:	
gamma/ °:	90	parameter 1 Left:	0.55(1)
V/ 10^6 pm^3	205.69400	parameter 2 Left:	0.000000
Overall displacement parameter:	0.000000	parameter 3 Left:	0.000000
		R (Bragg)/ %:	4.86963

Occupancy, atomic fract. coordinates and Biso for $LiYO_2$-LT, 50992-ICSD

Atom	Wyck.	s.o.f.	x	y	z	B/ 10^4 pm^2
Y1	4e	0.940000	0.266000	0.131200	0.020700	0.210000
Li1	4e	1.000000	0.791000	0.346000	0.068000	0.600000
O1	4e	1.000000	0.045900	0.107000	0.237800	0.280000
O2	4e	1.000000	0.512100	0.328800	0.343900	0.290000
Yb1	4e	0.050000	0.266000	0.131200	0.020700	0.210000
Er1	4e	0.010000	0.266000	0.131200	0.020700	0.210000

Relevant parameters of $LiYO_2$-HT, 50993-ICSD

Structure and profile data:		V/ 10^6 pm^3	202.62080
Formula sum: $Y_{3.76}Li_{4.00}O_{8.00}Yb_{0.20}Er_{0.04}$		Overall displacement parameter:	0.000000
Formula mass/ g/mol:	531.3438	Extinction:	0.000000
Density (calculated)/ g/cm^3	4.3539	Flat Plate Absorption Correction:	0.000000
F(000):	239.3600	Porosity:	0.000000
Weight fraction/ %:	0.000000	Roughness:	0.000000
Space group (No.):	I 41/a m d (141)	Fitting mode:	Structure Fit
		U Left:	0.068937
Lattice parameters:		V Left:	-0.052995
a/ A: 4(391707623424)		W Left:	0.027085
b/ A: 4(391707623424)		Preferred orientation direction/ hkl:	0.00 0.00 1.00
c/ A: 10(4880789929984)		Preferred orientation parameter:	1.000000
alpha/ °:	90	Asymmetry parameter 1:	0.170069
beta/ °:	90	Asymmetry parameter 2:	0.000000
gamma/ °:	90	Peak shape:	
		parameter 1 Left:	0.626711

parameter 2 Left:	0.000000	R (Bragg)/ %:	999.00000
parameter 3 Left:	0.000000		

Occupancy, atomic fract. coordinates and Biso for $LiYO_2$-HT, 50993-ICSD

Atom	Wyck.	s.o.f.	x	y	z	B/ 10^4 pm^2
Y1	4b	0.940000	0.000000	0.250000	0.375000	0.000000
Li1	4b	0.000000	0.000000	0.250000	0.375000	0.000000
Li2	4a	1.000000	0.000000	0.750000	0.125000	0.000000
O1	8e	1.000000	0.000000	0.250000	0.152000	0.000000
Yb1	4b	0.050000	0.000000	0.250000	0.375000	0.000000
Er1	4b	0.010000	0.000000	0.250000	0.375000	0.000000

The data for the $LiYO_2$:5%Yb^{3+}, 1%Er^{3+} at 140 K:

Global Parameters

Number of used phases:	3	Asymmetry Type:	No Asymmetry Function
Number of variables:	20		
Number of constraints:	2	Asymmetry 1:	0.000000
Zero shift/ °2Theta:	0.000000	Asymmetry 2:	0.000000
Specimen displacement/ mm :	0.263(1)	Shape Type:	Shape Individual
Profile function:	Pseudo Voigt	Shape 1 Left:	0.600000
Background:	Polynomial	Shape 2 Left:	0.000000
R (expected)/ %:	0.59218	Shape 3 Left:	0.000000
R (profile)/ %:	3.70089	Shape 1 Right:	0.600000
R (weighted profile)/ %:	5.34995	Shape 2 Right:	0.000000
GOF:	81.61800	Shape 3 Right:	0.000000
d-statistic:	0.16474	K a1/a2 intensity ratio:	0.500000
U standard:	0.000000	K alpha/beta intensity ratio:	0.000000
V standard:	0.000000	Crystal Shape Factor K:	1.0000
W standard:	0.010000	Instrumental FWHM Curve Type:	Caglioti function
U Left:	0.000000	Instr. Gauss Curve Coefficient A:	0.0045(5)
V Left:	0.000000	Instr. Gauss Curve Coefficient B:	-0.0032(9)
W Left:	0.010000	Instr. Gauss Curve Coefficient C:	0.0046(3)
U Right:	0.000000	Instr. Lorentz Curve Coefficient A:	0.0062(7)
V Right:	0.000000	Instr. Lorentz Curve Coefficient B:	-0.004(1)
W Right:	0.010000	Instr. Lorentz Curve Coefficient C:	0.0064(5)

Relevant parameters of $LiYO_2$-LT, 50992-ICSD

Structure and profile data:		Extinction:	0.000000
Formula sum: $Y_{3.76}Li_{4.00}O_{8.00}Yb_{0.20}Er_{0.04}$		Flat Plate Absorption Correction:	0.000000
Formula mass/ g/mol:	531.3438	Porosity:	0.000000
Density (calculated)/ g/cm^3	4.2891	Roughness:	0.000000
F(000):	239.3600	Fitting mode:	Structure Fit
Weight fraction/ %:	87.1(3)	U Left:	0.041(9)
Space group (No.):	P 1 21/c 1 (14)	V Left:	-0.008(7)
Lattice parameters:		W Left:	0.018(1)
a/ A:	6.1194(2)	Preferred orientation direction/ hkl:	0.00 0.00 1.00
b/ A:	6.1784(2)	Preferred orientation parameter:	1.000000
c/ A:	6.2057(2)	Asymmetry parameter 1:	0.10(2)
alpha/ °:	90	Asymmetry parameter 2:	0.000000
beta/ °:	118.762(2)	Peak shape:	
gamma/ °:	90	parameter 1 Left:	0.56(1)
V/ 10^6 pm^3	205.67980	parameter 2 Left:	0.000000
Overall displacement parameter:	0.000000	parameter 3 Left:	0.000000
		R (Bragg)/ %:	4.92714

Occupancy, atomic fract. coordinates and Biso for $LiYO_2$-LT, 50992-ICSD

Atom	Wyck.	s.o.f.	x	y	z	B/ 10^4 pm^2
Y1	4e	0.940000	0.266000	0.131200	0.020700	0.210000
Li1	4e	1.000000	0.791000	0.346000	0.068000	0.600000
O1	4e	1.000000	0.045900	0.107000	0.237800	0.280000
O2	4e	1.000000	0.512100	0.328800	0.343900	0.290000
Yb1	4e	0.050000	0.266000	0.131200	0.020700	0.210000
Er1	4e	0.010000	0.266000	0.131200	0.020700	0.210000

Relevant parameters of $LiYO_2$-HT, 50993-ICSD

Structure and profile data:		Density (calculated)/ g/cm^3	4.3539
Formula sum: $Y_{3.76}Li_{4.00}O_{8.00}Yb_{0.20}Er_{0.04}$		F(000):	239.3600
Formula mass/ g/mol:	531.3438	Weight fraction/ %:	0.000000
		Space group (No.): I 41/a m d (141)	

Lattice parameters:		Fitting mode:	Structure Fit
a/ A: 4(394794336256)		U Left:	0.068937
b/ A: 4(394794336256)		V Left:	-0.052995
c/ A: 10(4919251697664)		W Left:	0.027085
alpha/ °:	90	Preferred orientation direction/ hkl:	0.00 0.00 1.00
beta/ °:	90	Preferred orientation parameter:	1.000000
gamma/ °:	90	Asymmetry parameter 1:	0.170069
V/ 10^6 pm^3	202.62080	Asymmetry parameter 2:	0.000000
Overall displacement parameter:	0.000000	Peak shape:	
Extinction:	0.000000	parameter 1 Left:	0.626711
Flat Plate Absorption Correction:	0.000000	parameter 2 Left:	0.000000
Porosity:	0.000000	parameter 3 Left:	0.000000
Roughness:	0.000000	R (Bragg)/ %:	999.00000

Occupancy, atomic fract. coordinates and Biso for $LiYO_2$-HT, 50993-ICSD

Atom	Wyck.	s.o.f.	x	y	z	B/ 10^4 pm^2
Y1	4b	0.940000	0.000000	0.250000	0.375000	0.000000
Li1	4b	0.000000	0.000000	0.250000	0.375000	0.000000
Li2	4a	1.000000	0.000000	0.750000	0.125000	0.000000
O1	8e	1.000000	0.000000	0.250000	0.152000	0.000000
Yb1	4b	0.050000	0.000000	0.250000	0.375000	0.000000
Er1	4b	0.010000	0.000000	0.250000	0.375000	0.000000

The data for the $LiYO_2$:5%Yb^{3+}, 1%Er^{3+} at 160 K:

Global Parameters

Number of used phases:	3	Asymmetry Type:	No Asymmetry Function
Number of variables:	20		
Number of constraints:	2	Asymmetry 1:	0.000000
Zero shift/ °2Theta:	0.000000	Asymmetry 2:	0.000000
Specimen displacement/ mm :	0.258(1)	Shape Type:	Shape Individual
Profile function:	Pseudo Voigt	Shape 1 Left:	0.600000
Background:	Polynomial	Shape 2 Left:	0.000000
R (expected)/ %:	0.59255	Shape 3 Left:	0.000000
R (profile)/ %:	3.71782	Shape 1 Right:	0.600000
R (weighted profile)/ %:	5.42251	Shape 2 Right:	0.000000
GOF:	83.74342	Shape 3 Right:	0.000000
d-statistic:	0.15451	K a1/a2 intensity ratio:	0.500000
U standard:	0.000000	K alpha/beta intensity ratio:	0.000000
V standard:	0.000000	Crystal Shape Factor K:	1.0000
W standard:	0.010000	Instrumental FWHM Curve Type:	Caglioti function
U Left:	0.000000	Instr. Gauss Curve Coefficient A:	0.0045(5)
V Left:	0.000000	Instr. Gauss Curve Coefficient B:	-0.0032(9)
W Left:	0.010000	Instr. Gauss Curve Coefficient C:	0.0046(3)
U Right:	0.000000	Instr. Lorentz Curve Coefficient A:	0.0062(7)
V Right:	0.000000	Instr. Lorentz Curve Coefficient B:	-0.004(1)
W Right:	0.010000	Instr. Lorentz Curve Coefficient C:	0.0064(5)

Relevant parameters of $LiYO_2$-LT, 50992-ICSD

Structure and profile data:		Flat Plate Absorption Correction:	0.000000
Formula sum: $Y_{3.76}Li_{4.00}O_{8.00}Yb_{0.20}Er_{0.04}$		Porosity:	0.000000
Formula mass/ g/mol:	531.3438	Roughness:	0.000000
Density (calculated)/ g/cm^3	4.2896	Fitting mode:	Structure Fit
F(000):	239.3600	U Left:	0.05(1)
Weight fraction/ %:	87.3(3)	V Left:	-0.014(7)
Space group (No.):	P 1 21/c 1 (14)	W Left:	0.020(1)
Lattice parameters:		Preferred orientation direction/ hkl:	0.00 0.00 1.00
a/ A:	6.1174(2)	Preferred orientation parameter:	1.000000
b/ A:	6.1811(2)	Asymmetry parameter 1:	0.09(2)
c/ A:	6.2025(2)	Asymmetry parameter 2:	0.000000
alpha/ °:	90	Peak shape:	
beta/ °:	118.730(2)	parameter 1 Left:	0.54(1)
gamma/ °:	90	parameter 2 Left:	0.000000
V/ 10^6 pm^3	205.65860	parameter 3 Left:	0.000000
Overall displacement parameter:	0.000000	R (Bragg)/ %:	4.98985
Extinction:	0.000000		

Occupancy, atomic fract. coordinates and Biso for $LiYO_2$-LT, 50992-ICSD

Atom	Wyck.	s.o.f.	x	y	z	B/ 10^4 pm^2
Y1	4e	0.940000	0.266000	0.131200	0.020700	0.210000

Li1	4e	1.000000	0.791000	0.346000	0.068000	0.600000
O1	4e	1.000000	0.045900	0.107000	0.237800	0.280000
O2	4e	1.000000	0.512100	0.328800	0.343900	0.290000
Yb1	4e	0.050000	0.266000	0.131200	0.020700	0.210000
Er1	4e	0.010000	0.266000	0.131200	0.020700	0.210000

Relevant parameters of $LiYO_2$-HT, 50993-ICSD

Structure and profile data:		Extinction:	0.000000
Formula sum: $Y_{3.76}Li_{4.00}O_{8.00}Yb_{0.20}Er_{0.04}$		Flat Plate Absorption Correction:	0.000000
Formula mass/ g/mol:	531.3438	Porosity:	0.000000
Density (calculated)/ g/cm^3	4.3539	Roughness:	0.000000
F(000):	239.3600	Fitting mode:	Structure Fit
Weight fraction/ %:	0.000000	U Left:	0.068937
Space group (No.): I 41/a m d (141)		V Left:	-0.052995
Lattice parameters:		W Left:	0.027085
a/ A: 4(399901687808)		Preferred orientation direction/ hkl:	0.00 0.00 1.00
b/ A: 4(399901687808)		Preferred orientation parameter:	1.000000
c/ A: 10(4982890823680)		Asymmetry parameter 1:	0.170069
alpha/ °:	90	Asymmetry parameter 2:	0.000000
beta/ °:	90	Peak shape:	
gamma/ °:	90	parameter 1 Left:	0.626711
V/ 10^6 pm^3	202.62080	parameter 2 Left:	0.000000
Overall displacement parameter:	0.000000	parameter 3 Left:	0.000000
		R (Bragg)/ %:	999.00000

Occupancy, atomic fract. coordinates and Biso for $LiYO_2$-HT, 50993-ICSD

Atom	Wyck.	s.o.f.	x	y	z	B/ 10^4 pm^2
Y1	4b	0.940000	0.000000	0.250000	0.375000	0.000000
Li1	4b	0.000000	0.000000	0.250000	0.375000	0.000000
Li2	4a	1.000000	0.000000	0.750000	0.125000	0.000000
O1	8e	1.000000	0.000000	0.250000	0.152000	0.000000
Yb1	4b	0.050000	0.000000	0.250000	0.375000	0.000000
Er1	4b	0.010000	0.000000	0.250000	0.375000	0.000000

The data for the $LiYO_2$:5%Yb^{3+}, 1%Er^{3+} at 180 K:

Global Parameters

Number of used phases:	3		Function
Number of variables:	20	Asymmetry 1:	0.000000
Number of constraints:	2	Asymmetry 2:	0.000000
Zero shift/ °2Theta:	0.000000	Shape Type:	Shape Individual
Specimen displacement/ mm :	0.254(1)	Shape 1 Left:	0.600000
Profile function:	Pseudo Voigt	Shape 2 Left:	0.000000
Background:	Polynomial	Shape 3 Left:	0.000000
R (expected)/ %:	0.59345	Shape 1 Right:	0.600000
R (profile)/ %:	3.75798	Shape 2 Right:	0.000000
R (weighted profile)/ %:	5.46123	Shape 3 Right:	0.000000
GOF:	84.68628	K a1/a2 intensity ratio:	0.500000
d-statistic:	0.15381	K alpha/beta intensity ratio:	0.000000
U standard:	0.000000	Crystal Shape Factor K:	1.0000
V standard:	0.000000	Instrumental FWHM Curve Type:	Caglioti function
W standard:	0.010000	Instr. Gauss Curve Coefficient A:	0.0045(5)
U Left:	0.000000	Instr. Gauss Curve Coefficient B:	-0.0032(9)
V Left:	0.000000	Instr. Gauss Curve Coefficient C:	0.0046(3)
W Left:	0.010000	Instr. Lorentz Curve Coefficient A:	0.0062(7)
U Right:	0.000000	Instr. Lorentz Curve Coefficient B:	-0.004(1)
V Right:	0.000000	Instr. Lorentz Curve Coefficient C:	0.0064(5)
W Right:	0.010000		
Asymmetry Type:	No Asymmetry		

Relevant parameters of $LiYO_2$-LT, 50992-ICSD

Structure and profile data:		a/ A:	6.1151(2)
Formula sum: $Y_{3.76}Li_{4.00}O_{8.00}Yb_{0.20}Er_{0.04}$		b/ A:	6.1841(2)
Formula mass/ g/mol:	531.3438	c/ A:	6.1988(2)
Density (calculated)/ g/cm^3	4.2904	alpha/ °:	90
F(000):	239.3600	beta/ °:	118.698(2)
Weight fraction/ %:	87.2(3)	gamma/ °:	90
Space group (No.):	P 1 21/c 1 (14)	V/ 10^6 pm^3	205.62040
Lattice parameters:		Overall displacement parameter:	0.000000

Extinction:	0.000000	Preferred orientation parameter:	1.000000
Flat Plate Absorption Correction:	0.000000	Asymmetry parameter 1:	0.09(2)
Porosity:	0.000000	Asymmetry parameter 2:	0.000000
Roughness:	0.000000	Peak shape:	
Fitting mode:	Structure Fit	parameter 1 Left:	0.54(1)
U Left:	0.05(1)	parameter 2 Left:	0.000000
V Left:	-0.018(8)	parameter 3 Left:	0.000000
W Left:	0.020(1)	R (Bragg)/ %:	5.12497
Preferred orientation direction/ hkl:	0.00 0.00 1.00		

Occupancy, atomic fract. coordinates and Biso for $LiYO_2$-LT, 50992-ICSD

Atom	Wyck.	s.o.f.	x	y	z	B/ 10^4 pm^2
Y1	4e	0.940000	0.266000	0.131200	0.020700	0.210000
Li1	4e	1.000000	0.791000	0.346000	0.068000	0.600000
O1	4e	1.000000	0.045900	0.107000	0.237800	0.280000
O2	4e	1.000000	0.512100	0.328800	0.343900	0.290000
Yb1	4e	0.050000	0.266000	0.131200	0.020700	0.210000
Er1	4e	0.010000	0.266000	0.131200	0.020700	0.210000

Relevant parameters of $LiYO_2$-HT, 50993-ICSD

Structure and profile data:		Extinction:	0.000000
Formula sum: $Y_{3.76}Li_{4.00}O_{8.00}Yb_{0.20}Er_{0.04}$		Flat Plate Absorption Correction:	0.000000
Formula mass/ g/mol:	531.3438	Porosity:	0.000000
Density (calculated)/ g/cm^3	4.3539	Roughness:	0.000000
F(000):	239.3600	Fitting mode:	Structure Fit
Weight fraction/ %:	0.000000	U Left:	0.068937
Space group (No.): I 41/a m d (141)		V Left:	-0.052995
Lattice parameters:		W Left:	0.027085
a/ A: 4(402146656256)		Preferred orientation direction/ hkl:	0.00 0.00 1.00
b/ A: 4(402146656256)		Preferred orientation parameter:	1.000000
c/ A: 10(5010863685632)		Asymmetry parameter 1:	0.170069
alpha/ °:	90	Asymmetry parameter 2:	0.000000
beta/ °:	90	Peak shape:	
gamma/ °:	90	parameter 1 Left:	0.626711
V/ 10^6 pm^3	202.62080	parameter 2 Left:	0.000000
Overall displacement parameter:	0.000000	parameter 3 Left:	0.000000
		R (Bragg)/ %:	999.00000

Occupancy, atomic fract. coordinates and Biso for $LiYO_2$-HT, 50993-ICSD

Atom	Wyck.	s.o.f.	x	y	z	B/ 10^4 pm^2
Y1	4b	0.940000	0.000000	0.250000	0.375000	0.000000
Li1	4b	0.000000	0.000000	0.250000	0.375000	0.000000
Li2	4a	1.000000	0.000000	0.750000	0.125000	0.000000
O1	8e	1.000000	0.000000	0.250000	0.152000	0.000000
Yb1	4b	0.050000	0.000000	0.250000	0.375000	0.000000
Er1	4b	0.010000	0.000000	0.250000	0.375000	0.000000

The data for the $LiYO_2$:5%Yb^{3+}, 1%Er^{3+} at 190 K:

Global Parameters

Number of used phases:	3	V Right:	0.000000
Number of variables:	20	W Right:	0.010000
Number of constraints:	2	Asymmetry Type:	No Asymmetry Function
Zero shift/ °2Theta:	0.000000		
Specimen displacement/ mm :	0.249(1)	Asymmetry 1:	0.000000
Profile function:	Pseudo Voigt	Asymmetry 2:	0.000000
Background:	Polynomial	Shape Type:	Shape Individual
R (expected)/ %:	0.59133	Shape 1 Left:	0.600000
R (profile)/ %:	3.73461	Shape 2 Left:	0.000000
R (weighted profile)/ %:	5.42317	Shape 3 Left:	0.000000
GOF:	84.10866	Shape 1 Right:	0.600000
d-statistic:	0.16134	Shape 2 Right:	0.000000
U standard:	0.000000	Shape 3 Right:	0.000000
V standard:	0.000000	K a1/a2 intensity ratio:	0.500000
W standard:	0.010000	K alpha/beta intensity ratio:	0.000000
U Left:	0.000000	Crystal Shape Factor K:	1.0000
V Left:	0.000000	Instrumental FWHM Curve Type:	Caglioti function
W Left:	0.010000	Instr. Gauss Curve Coefficient A:	0.0045(5)
U Right:	0.000000	Instr. Gauss Curve Coefficient B:	-0.0032(9)

Instr. Gauss Curve Coefficient C:	0.0046(3)	Instr. Lorentz Curve Coefficient B:	-0.004(1)
Instr. Lorentz Curve Coefficient A:	0.0062(7)	Instr. Lorentz Curve Coefficient C:	0.0064(5)

Relevant parameters of $LiYO_2$-LT, 50992-ICSD

Structure and profile data:		Extinction:	0.000000
Formula sum: $Y_{3.76}Li_{4.00}O_{8.00}Yb_{0.20}Er_{0.04}$		Flat Plate Absorption Correction:	0.000000
Formula mass/ g/mol:	531.3438	Porosity:	0.000000
Density (calculated)/ g/cm^3	4.2903	Roughness:	0.000000
F(000):	239.3600	Fitting mode:	Structure Fit
Weight fraction/ %:	87.4(3)	U Left:	0.06(1)
Space group (No.):	P 1 21/c 1 (14)	V Left:	-0.020(8)
Lattice parameters:		W Left:	0.021(1)
a/ A:	6.1142(2)	Preferred orientation direction/ hkl:	0.00 0.00 1.00
b/ A:	6.1859(2)	Preferred orientation parameter:	1.000000
c/ A:	6.1971(2)	Asymmetry parameter 1:	0.11(2)
alpha/ °:	90	Asymmetry parameter 2:	0.000000
beta/ °:	118.682(2)	Peak shape:	
gamma/ °:	90	parameter 1 Left:	0.54(1)
V/ 10^6 pm^3	205.62460	parameter 2 Left:	0.000000
Overall displacement parameter:	0.000000	parameter 3 Left:	0.000000
		R (Bragg)/ %:	4.82554

Occupancy, atomic fract. coordinates and Biso for $LiYO_2$-LT, 50992-ICSD

Atom	Wyck.	s.o.f.	x	y	z	B/ 10^4 pm^2
Y1	4e	0.940000	0.266000	0.131200	0.020700	0.210000
Li1	4e	1.000000	0.791000	0.346000	0.068000	0.600000
O1	4e	1.000000	0.045900	0.107000	0.237800	0.280000
O2	4e	1.000000	0.512100	0.328800	0.343900	0.290000
Yb1	4e	0.050000	0.266000	0.131200	0.020700	0.210000
Er1	4e	0.010000	0.266000	0.131200	0.020700	0.210000

Relevant parameters of $LiYO_2$-HT, 50993-ICSD

Structure and profile data:		Extinction:	0.000000
Formula sum: $Y_{3.76}Li_{4.00}O_{8.00}Yb_{0.20}Er_{0.04}$		Flat Plate Absorption Correction:	0.000000
Formula mass/ g/mol:	531.3438	Porosity:	0.000000
Density (calculated)/ g/cm^3	4.3539	Roughness:	0.000000
F(000):	239.3600	Fitting mode:	Structure Fit
Weight fraction/ %:	0.000000	U Left:	0.068937
Space group (No.): I 41/a m d (141)		V Left:	-0.052995
Lattice parameters:		W Left:	0.027085
a/ A: 4(400772825088)		Preferred orientation direction/ hkl:	0.00 0.00 1.00
b/ A: 4(400772825088)		Preferred orientation parameter:	1.000000
c/ A:10(4993745158144)		Asymmetry parameter 1:	0.170069
alpha/ °:	90	Asymmetry parameter 2:	0.000000
beta/ °:	90	Peak shape:	
gamma/ °:	90	parameter 1 Left:	0.626711
V/ 10^6 pm^3	202.62080	parameter 2 Left:	0.000000
Overall displacement parameter:	0.000000	parameter 3 Left:	0.000000
		R (Bragg)/ %:	999.00000

Occupancy, atomic fract. coordinates and Biso for $LiYO_2$-HT, 50993-ICSD

Atom	Wyck.	s.o.f.	x	y	z	B/ 10^4 pm^2
Y1	4b	0.940000	0.000000	0.250000	0.375000	0.000000
Li1	4b	0.000000	0.000000	0.250000	0.375000	0.000000
Li2	4a	1.000000	0.000000	0.750000	0.125000	0.000000
O1	8e	1.000000	0.000000	0.250000	0.152000	0.000000
Yb1	4b	0.050000	0.000000	0.250000	0.375000	0.000000
Er1	4b	0.010000	0.000000	0.250000	0.375000	0.000000

The data for the $LiYO_2$:5%Yb^{3+}, 1%Er^{3+} at 200 K:

Global Parameters

Number of used phases:	3	R (weighted profile)/ %:	5.42193
Number of variables:	20	GOF:	84.41157
Number of constraints:	2	d-statistic:	0.16156
Zero shift/ °2Theta:	0.000000	U standard:	0.000000
Specimen displacement/ mm :	0.248(1)	V standard:	0.000000
Profile function:	Pseudo Voigt	W standard:	0.010000
Background:	Polynomial	U Left:	0.000000
R (expected)/ %:	0.59014	V Left:	0.000000
R (profile)/ %:	3.72705	W Left:	0.010000

U Right:	0.000000	Shape 2 Right:	0.000000
V Right:	0.000000	Shape 3 Right:	0.000000
W Right:	0.010000	K a1/a2 intensity ratio:	0.500000
Asymmetry Type:	No Asymmetry Function	K alpha/beta intensity ratio:	0.000000
		Crystal Shape Factor K:	1.0000
Asymmetry 1:	0.000000	Instrumental FWHM Curve Type:	Caglioti function
Asymmetry 2:	0.000000	Instr. Gauss Curve Coefficient A:	0.0045(5)
Shape Type:	Shape Individual	Instr. Gauss Curve Coefficient B:	-0.0032(9)
Shape 1 Left:	0.600000	Instr. Gauss Curve Coefficient C:	0.0046(3)
Shape 2 Left:	0.000000	Instr. Lorentz Curve Coefficient A:	0.0062(7)
Shape 3 Left:	0.000000	Instr. Lorentz Curve Coefficient B:	-0.004(1)
Shape 1 Right:	0.600000	Instr. Lorentz Curve Coefficient C:	0.0064(5)

Relevant parameters of $LiYO_2$-LT, 50992-ICSD

Structure and profile data:		Extinction:	0.000000
Formula sum: $Y_{3.76}Li_{4.00}O_{8.00}Yb_{0.20}Er_{0.04}$		Flat Plate Absorption Correction:	0.000000
Formula mass/ g/mol:	531.3438	Porosity:	0.000000
Density (calculated)/ g/cm^3	4.2906	Roughness:	0.000000
F(000):	239.3600	Fitting mode:	Structure Fit
Weight fraction/ %:	87.3(3)	U Left:	0.06(1)
Space group (No.):	P 1 21/c 1 (14)	V Left:	-0.022(8)
Lattice parameters:		W Left:	0.020(1)
a/ A:	6.1132(2)	Preferred orientation direction/ hkl:	0.00 0.00 1.00
b/ A:	6.1876(2)	Preferred orientation parameter:	1.000000
c/ A:	6.1951(2)	Asymmetry parameter 1:	0.09(2)
alpha/ °:	90	Asymmetry parameter 2:	0.000000
beta/ °:	118.667(2)	Peak shape:	
gamma/ °:	90	parameter 1 Left:	0.54(1)
V/ 10^6 pm^3	205.60950	parameter 2 Left:	0.000000
Overall displacement parameter:	0.000000	parameter 3 Left:	0.000000
		R (Bragg)/ %:	4.82878

Occupancy, atomic fract. coordinates and Biso for $LiYO_2$-LT, 50992-ICSD

Atom	Wyck.	s.o.f.	x	y	z	B/ 10^4 pm^2
Y1	4e	0.940000	0.266000	0.131200	0.020700	0.210000
Li1	4e	1.000000	0.791000	0.346000	0.068000	0.600000
O1	4e	1.000000	0.045900	0.107000	0.237800	0.280000
O2	4e	1.000000	0.512100	0.328800	0.343900	0.290000
Yb1	4e	0.050000	0.266000	0.131200	0.020700	0.210000
Er1	4e	0.010000	0.266000	0.131200	0.020700	0.210000

Relevant parameters of $LiYO_2$-HT, 50993-ICSD

Structure and profile data:		Extinction:	0.000000
Formula sum: $Y_{3.76}Li_{4.00}O_{8.00}Yb_{0.20}Er_{0.04}$		Flat Plate Absorption Correction:	0.000000
Formula mass/ g/mol:	531.3438	Porosity:	0.000000
Density (calculated)/ g/cm^3	4.3539	Roughness:	0.000000
F(000):	239.3600	Fitting mode:	Structure Fit
Weight fraction/ %:	0.000000	U Left:	0.068937
Space group (No.): I 41/a m d (141)		V Left:	-0.052995
Lattice parameters:		W Left:	0.027085
a/ A: 4(401493852160)		Preferred orientation direction/ hkl:	0.00 0.00 1.00
b/ A: 4(401493852160)		Preferred orientation parameter:	1.000000
c/ A: 10(5002729881600)		Asymmetry parameter 1:	0.170069
alpha/ °:	90	Asymmetry parameter 2:	0.000000
beta/ °:	90	Peak shape:	
gamma/ °:	90	parameter 1 Left:	0.626711
V/ 10^6 pm^3	202.62080	parameter 2 Left:	0.000000
Overall displacement parameter:	0.000000	parameter 3 Left:	0.000000
		R (Bragg)/ %:	999.00000

Occupancy, atomic fract. coordinates and Biso for $LiYO_2$-HT, 50993-ICSD

Atom	Wyck.	s.o.f.	x	y	z	B/ 10^4 pm^2
Y1	4b	0.940000	0.000000	0.250000	0.375000	0.000000
Li1	4b	0.000000	0.000000	0.250000	0.375000	0.000000
Li2	4a	1.000000	0.000000	0.750000	0.125000	0.000000
O1	8e	1.000000	0.000000	0.250000	0.152000	0.000000
Yb1	4b	0.050000	0.000000	0.250000	0.375000	0.000000
Er1	4b	0.010000	0.000000	0.250000	0.375000	0.000000

The data for the $LiYO_2$:5%Yb^{3+}, 1%Er^{3+} at 210 K:

Global Parameters

Number of used phases:	3		Function
Number of variables:	21	Asymmetry 1:	0.000000
Number of constraints:	2	Asymmetry 2:	0.000000
Zero shift/ °2Theta:	0.000000	Shape Type:	Shape Individual
Specimen displacement/ mm :	0.243(1)	Shape 1 Left:	0.600000
Profile function:	Pseudo Voigt	Shape 2 Left:	0.000000
Background:	Polynomial	Shape 3 Left:	0.000000
R (expected)/ %:	0.59082	Shape 1 Right:	0.600000
R (profile)/ %:	3.71584	Shape 2 Right:	0.000000
R (weighted profile)/ %:	5.42113	Shape 3 Right:	0.000000
GOF:	84.19193	K a1/a2 intensity ratio:	0.500000
d-statistic:	0.16573	K alpha/beta intensity ratio:	0.000000
U standard:	0.000000	Crystal Shape Factor K:	1.0000
V standard:	0.000000	Instrumental FWHM Curve Type:	Caglioti function
W standard:	0.010000	Instr. Gauss Curve Coefficient A:	0.0045(5)
U Left:	0.000000	Instr. Gauss Curve Coefficient B:	-0.0032(9)
V Left:	0.000000	Instr. Gauss Curve Coefficient C:	0.0046(3)
W Left:	0.010000	Instr. Lorentz Curve Coefficient A:	0.0062(7)
U Right:	0.000000	Instr. Lorentz Curve Coefficient B:	-0.004(1)
V Right:	0.000000	Instr. Lorentz Curve Coefficient C:	0.0064(5)
W Right:	0.010000		
Asymmetry Type:	No Asymmetry		

Relevant parameters of $LiYO_2$-LT, 50992-ICSD

Structure and profile data:		Flat Plate Absorption Correction:	0.000000
Formula sum: $Y_{3.76}Li_{4.00}O_{8.00}Yb_{0.20}Er_{0.04}$		Porosity:	0.000000
Formula mass/ g/mol:	531.3438	Roughness:	0.000000
Density (calculated)/ g/cm^3	4.2905	Fitting mode:	Structure Fit
F(000):	239.3600	U Left:	0.07(1)
Weight fraction/ %:	83.7(3)	V Left:	-0.028(8)
Space group (No.):	P 1 21/c 1 (14)	W Left:	0.022(1)
Lattice parameters:		Preferred orientation direction/ hkl:	0.00 0.00 1.00
a/ A:	6.1118(2)	Preferred orientation parameter:	1.000000
b/ A:	6.1900(2)	Asymmetry parameter 1:	0.12(2)
c/ A:	6.1933(2)	Asymmetry parameter 2:	0.000000
alpha/ °:	90	Peak shape:	
beta/ °:	118.652(2)	parameter 1 Left:	0.53(1)
gamma/ °:	90	parameter 2 Left:	0.000000
V/ 10^6 pm^3	205.61320	parameter 3 Left:	0.000000
Overall displacement parameter:	0.000000	R (Bragg)/ %:	4.97795
Extinction:	0.000000		

Occupancy, atomic fract. coordinates and Biso for $LiYO_2$-LT, 50992-ICSD

Atom	Wyck.	s.o.f.	x	y	z	B/ 10^4 pm^2
Y1	4e	0.940000	0.266000	0.131200	0.020700	0.210000
Li1	4e	1.000000	0.791000	0.346000	0.068000	0.600000
O1	4e	1.000000	0.045900	0.107000	0.237800	0.280000
O2	4e	1.000000	0.512100	0.328800	0.343900	0.290000
Yb1	4e	0.050000	0.266000	0.131200	0.020700	0.210000
Er1	4e	0.010000	0.266000	0.131200	0.020700	0.210000

Relevant parameters of $LiYO_2$-HT, 50993-ICSD

Structure and profile data:		Extinction:	0.000000
Formula sum: $Y_{3.76}Li_{4.00}O_{8.00}Yb_{0.20}Er_{0.04}$		Flat Plate Absorption Correction:	0.000000
Formula mass/ g/mol:	531.3438	Porosity:	0.000000
Density (calculated)/ g/cm^3	4.3355	Roughness:	0.000000
F(000):	239.3600	Fitting mode:	Structure Fit
Weight fraction/ %:	3.3(2)	U Left:	0.068937
Space group (No.): I 41/a m d (141)		V Left:	-0.052995
Lattice parameters:		W Left:	0.027085
a/ A:	4.4370(7)	Preferred orientation direction/ hkl:	0.00 0.00 1.00
b/ A:	4.4370(7)	Preferred orientation parameter:	1.000000
c/ A:	10.336(3)	Asymmetry parameter 1:	0.170069
alpha/ °:	90	Asymmetry parameter 2:	0.000000
beta/ °:	90	Peak shape:	
gamma/ °:	90	parameter 1 Left:	0.626711
V/ 10^6 pm^3	203.48010	parameter 2 Left:	0.000000
Overall displacement parameter:	0.000000	parameter 3 Left:	0.000000
		R (Bragg)/ %:	3.27337

Occupancy, atomic fract. coordinates and Biso for $LiYO_2$-HT, 50993-ICSD

Atom	Wyck.	s.o.f.	x	y	z	B/ 10^4 pm^2
Y1	4b	0.940000	0.000000	0.250000	0.375000	0.000000
Li1	4b	0.000000	0.000000	0.250000	0.375000	0.000000
Li2	4a	1.000000	0.000000	0.750000	0.125000	0.000000
O1	8e	1.000000	0.000000	0.250000	0.152000	0.000000
Yb1	4b	0.050000	0.000000	0.250000	0.375000	0.000000
Er1	4b	0.010000	0.000000	0.250000	0.375000	0.000000

The data for the $LiYO_2$:5%Yb^{3+}, 1%Er^{3+} at 220 K:

Global Parameters

Number of used phases:	3	Asymmetry Type:	No Asymmetry Function
Number of variables:	21		
Number of constraints:	2	Asymmetry 1:	0.000000
Zero shift/ °2Theta:	0.000000	Asymmetry 2:	0.000000
Specimen displacement/ mm :	0.240(1)	Shape Type:	Shape Individual
Profile function:	Pseudo Voigt	Shape 1 Left:	0.600000
Background:	Polynomial	Shape 2 Left:	0.000000
R (expected)/ %:	0.58982	Shape 3 Left:	0.000000
R (profile)/ %:	3.67136	Shape 1 Right:	0.600000
R (weighted profile)/ %:	5.31341	Shape 2 Right:	0.000000
GOF:	81.15485	Shape 3 Right:	0.000000
d-statistic:	0.17550	K a1/a2 intensity ratio:	0.500000
U standard:	0.000000	K alpha/beta intensity ratio:	0.000000
V standard:	0.000000	Crystal Shape Factor K:	1.0000
W standard:	0.010000	Instrumental FWHM Curve Type:	Caglioti function
U Left:	0.000000	Instr. Gauss Curve Coefficient A:	0.0045(5)
V Left:	0.000000	Instr. Gauss Curve Coefficient B:	-0.0032(9)
W Left:	0.010000	Instr. Gauss Curve Coefficient C:	0.0046(3)
U Right:	0.000000	Instr. Lorentz Curve Coefficient A:	0.0062(7)
V Right:	0.000000	Instr. Lorentz Curve Coefficient B:	-0.004(1)
W Right:	0.010000	Instr. Lorentz Curve Coefficient C:	0.0064(5)

Relevant parameters of $LiYO_2$-LT, 50992-ICSD

Structure and profile data:		Extinction:	0.000000
Formula sum: $Y_{3.76}Li_{4.00}O_{8.00}Yb_{0.20}Er_{0.04}$		Flat Plate Absorption Correction:	0.000000
Formula mass/ g/mol:	531.3438	Porosity:	0.000000
Density (calculated)/ g/cm^3	4.2904	Roughness:	0.000000
F(000):	239.3600	Fitting mode:	Structure Fit
Weight fraction/ %:	82.7(3)	U Left:	0.06(1)
Space group (No.):	P 1 21/c 1 (14)	V Left:	-0.029(8)
Lattice parameters:		W Left:	0.023(1)
a/ A:	6.1107(2)	Preferred orientation direction/ hkl:	0.00 0.00 1.00
b/ A:	6.1924(2)	Preferred orientation parameter:	1.000000
c/ A:	6.1909(2)	Asymmetry parameter 1:	0.09(2)
alpha/ °:	90	Asymmetry parameter 2:	0.000000
beta/ °:	118.631(2)	Peak shape:	
gamma/ °:	90	parameter 1 Left:	0.55(1)
V/ 10^6 pm^3	205.61900	parameter 2 Left:	0.000000
Overall displacement parameter:	0.000000	parameter 3 Left:	0.000000
		R (Bragg)/ %:	4.76319

Occupancy, atomic fract. coordinates and Biso for $LiYO_2$-LT, 50992-ICSD

Atom	Wyck.	s.o.f.	x	y	z	B/ 10^4 pm^2
Y1	4e	0.940000	0.266000	0.131200	0.020700	0.210000
Li1	4e	1.000000	0.791000	0.346000	0.068000	0.600000
O1	4e	1.000000	0.045900	0.107000	0.237800	0.280000
O2	4e	1.000000	0.512100	0.328800	0.343900	0.290000
Yb1	4e	0.050000	0.266000	0.131200	0.020700	0.210000
Er1	4e	0.010000	0.266000	0.131200	0.020700	0.210000

Relevant parameters of $LiYO_2$-HT, 50993-ICSD

Structure and profile data:		Lattice parameters:	
Formula sum: $Y_{3.76}Li_{4.00}O_{8.00}Yb_{0.20}Er_{0.04}$		a/ A:	4.4383(6)
Formula mass/ g/mol:	531.3438	b/ A:	4.4383(6)
Density (calculated)/ g/cm^3	4.3394	c/ A:	10.320(2)
F(000):	239.3600	alpha/ °:	90
Weight fraction/ %:	4.4(2)	beta/ °:	90
Space group (No.): I 41/a m d (141)		gamma/ °:	90

V/ 10^6 pm^3	203.29800	Preferred orientation direction/ hkl:	0.00 0.00 1.00
Overall displacement parameter:	0.000000	Preferred orientation parameter:	1.000000
Extinction:	0.000000	Asymmetry parameter 1:	0.170069
Flat Plate Absorption Correction:	0.000000	Asymmetry parameter 2:	0.000000
Porosity:	0.000000	Peak shape:	
Roughness:	0.000000	parameter 1 Left:	0.626711
Fitting mode:	Structure Fit	parameter 2 Left:	0.000000
U Left:	0.068937	parameter 3 Left:	0.000000
V Left:	-0.052995	R (Bragg)/ %:	3.58031
W Left:	0.027085		

Occupancy, atomic fract. coordinates and Biso for $LiYO_2$-HT, 50993-ICSD

Atom	Wyck.	s.o.f.	x	y	z	B/ 10^4 pm^2
Y1	4b	0.940000	0.000000	0.250000	0.375000	0.000000
Li1	4b	0.000000	0.000000	0.250000	0.375000	0.000000
Li2	4a	1.000000	0.000000	0.750000	0.125000	0.000000
O1	8e	1.000000	0.000000	0.250000	0.152000	0.000000
Yb1	4b	0.050000	0.000000	0.250000	0.375000	0.000000
Er1	4b	0.010000	0.000000	0.250000	0.375000	0.000000

The data for the $LiYO_2$:5%Yb^{3+}, 1%Er^{3+} at 230 K:

Global Parameters

Number of used phases:	3	Asymmetry Type:	No Asymmetry Function
Number of variables:	21		
Number of constraints:	2	Asymmetry 1:	0.000000
Zero shift/ °2Theta:	0.000000	Asymmetry 2:	0.000000
Specimen displacement/ mm :	0.234(1)	Shape Type:	Shape Individual
Profile function:	Pseudo Voigt	Shape 1 Left:	0.600000
Background:	Polynomial	Shape 2 Left:	0.000000
R (expected)/ %:	0.58810	Shape 3 Left:	0.000000
R (profile)/ %:	3.65755	Shape 1 Right:	0.600000
R (weighted profile)/ %:	5.22901	Shape 2 Right:	0.000000
GOF:	79.05634	Shape 3 Right:	0.000000
d-statistic:	0.18395	K a1/a2 intensity ratio:	0.500000
U standard:	0.000000	K alpha/beta intensity ratio:	0.000000
V standard:	0.000000	Crystal Shape Factor K:	1.0000
W standard:	0.010000	Instrumental FWHM Curve Type:	Caglioti function
U Left:	0.000000	Instr. Gauss Curve Coefficient A:	0.0045(5)
V Left:	0.000000	Instr. Gauss Curve Coefficient B:	-0.0032(9)
W Left:	0.010000	Instr. Gauss Curve Coefficient C:	0.0046(3)
U Right:	0.000000	Instr. Lorentz Curve Coefficient A:	0.0062(7)
V Right:	0.000000	Instr. Lorentz Curve Coefficient B:	-0.004(1)
W Right:	0.010000	Instr. Lorentz Curve Coefficient C:	0.0064(5)

Relevant parameters of $LiYO_2$-LT, 50992-ICSD

Structure and profile data:		Extinction:	0.000000
Formula sum: $Y_{3.76}Li_{4.00}O_{8.00}Yb_{0.20}Er_{0.04}$		Flat Plate Absorption Correction:	0.000000
Formula mass/ g/mol:	531.3438	Porosity:	0.000000
Density (calculated)/ g/cm^3	4.2906	Roughness:	0.000000
F(000):	239.3600	Fitting mode:	Structure Fit
Weight fraction/ %:	79.7(3)	U Left:	0.06(1)
Space group (No.):	P 1 21/c 1 (14)	V Left:	-0.029(7)
Lattice parameters:		W Left:	0.022(1)
a/ A:	6.1094(2)	Preferred orientation direction/ hkl:	0.00 0.00 1.00
b/ A:	6.1948(2)	Preferred orientation parameter:	1.000000
c/ A:	6.1882(2)	Asymmetry parameter 1:	0.10(2)
alpha/ °:	90	Asymmetry parameter 2:	0.000000
beta/ °:	118.608(2)	Peak shape:	
gamma/ °:	90	parameter 1 Left:	0.60(1)
V/ 10^6 pm^3	205.60920	parameter 2 Left:	0.000000
Overall displacement parameter:	0.000000	parameter 3 Left:	0.000000
		R (Bragg)/ %:	4.61754

Occupancy, atomic fract. coordinates and Biso for $LiYO_2$-LT, 50992-ICSD

Atom	Wyck.	s.o.f.	x	y	z	B/ 10^4 pm^2
Y1	4e	0.940000	0.266000	0.131200	0.020700	0.210000
Li1	4e	1.000000	0.791000	0.346000	0.068000	0.600000
O1	4e	1.000000	0.045900	0.107000	0.237800	0.280000
O2	4e	1.000000	0.512100	0.328800	0.343900	0.290000
Yb1	4e	0.050000	0.266000	0.131200	0.020700	0.210000
Er1	4e	0.010000	0.266000	0.131200	0.020700	0.210000

Relevant parameters of $LiYO_2$-HT, 50993-ICSD

Structure and profile data:		Extinction:	0.000000
Formula sum: $Y_{3.76}Li_{4.00}O_{8.00}Yb_{0.20}Er_{0.04}$		Flat Plate Absorption Correction:	0.000000
Formula mass/ g/mol:	531.3438	Porosity:	0.000000
Density (calculated)/ g/cm^3	4.3381	Roughness:	0.000000
F(000):	239.3600	Fitting mode:	Structure Fit
Weight fraction/ %:	7.5(2)	U Left:	0.068937
Space group (No.): I 41/a m d (141)		V Left:	-0.052995
Lattice parameters:		W Left:	0.027085
a/ A:	4.4391(3)	Preferred orientation direction/ hkl:	0.00 0.00 1.00
b/ A:	4.4391(3)	Preferred orientation parameter:	1.000000
c/ A:	10.320(1)	Asymmetry parameter 1:	0.170069
alpha/ °:	90	Asymmetry parameter 2:	0.000000
beta/ °:	90	Peak shape:	
gamma/ °:	90	parameter 1 Left:	0.626711
V/ 10^6 pm^3	203.35910	parameter 2 Left:	0.000000
Overall displacement parameter:	0.000000	parameter 3 Left:	0.000000
		R (Bragg)/ %:	4.43187

Occupancy, atomic fract. coordinates and Biso for $LiYO_2$-HT, 50993-ICSD

Atom	Wyck.	s.o.f.	x	y	z	B/ 10^4 pm^2
Y1	4b	0.940000	0.000000	0.250000	0.375000	0.000000
Li1	4b	0.000000	0.000000	0.250000	0.375000	0.000000
Li2	4a	1.000000	0.000000	0.750000	0.125000	0.000000
O1	8e	1.000000	0.000000	0.250000	0.152000	0.000000
Yb1	4b	0.050000	0.000000	0.250000	0.375000	0.000000
Er1	4b	0.010000	0.000000	0.250000	0.375000	0.000000

The data for the $LiYO_2$:5%Yb^{3+}, 1%Er^{3+} at 240 K:

Global Parameters

Number of used phases:	3	Asymmetry Type:	No Asymmetry Function
Number of variables:	21		
Number of constraints:	2	Asymmetry 1:	0.000000
Zero shift/ °2Theta:	0.000000	Asymmetry 2:	0.000000
Specimen displacement/ mm :	0.232(1)	Shape Type:	Shape Individual
Profile function:	Pseudo Voigt	Shape 1 Left:	0.600000
Background:	Polynomial	Shape 2 Left:	0.000000
R (expected)/ %:	0.58916	Shape 3 Left:	0.000000
R (profile)/ %:	3.69303	Shape 1 Right:	0.600000
R (weighted profile)/ %:	5.22014	Shape 2 Right:	0.000000
GOF:	78.50413	Shape 3 Right:	0.000000
d-statistic:	0.19771	K a1/a2 intensity ratio:	0.500000
U standard:	0.000000	K alpha/beta intensity ratio:	0.000000
V standard:	0.000000	Crystal Shape Factor K:	1.0000
W standard:	0.010000	Instrumental FWHM Curve Type:	Caglioti function
U Left:	0.000000	Instr. Gauss Curve Coefficient A:	0.0045(5)
V Left:	0.000000	Instr. Gauss Curve Coefficient B:	-0.0032(9)
W Left:	0.010000	Instr. Gauss Curve Coefficient C:	0.0046(3)
U Right:	0.000000	Instr. Lorentz Curve Coefficient A:	0.0062(7)
V Right:	0.000000	Instr. Lorentz Curve Coefficient B:	-0.004(1)
W Right:	0.010000	Instr. Lorentz Curve Coefficient C:	0.0064(5)

Relevant parameters of $LiYO_2$-LT, 50992-ICSD

Structure and profile data:		V/ 10^6 pm^3	205.55930
Formula sum: $Y_{3.76}Li_{4.00}O_{8.00}Yb_{0.20}Er_{0.04}$		Overall displacement parameter:	0.000000
Formula mass/ g/mol:	531.3438	Extinction:	0.000000
Density (calculated)/ g/cm^3	4.2917	Flat Plate Absorption Correction:	0.000000
F(000):	239.3600	Porosity:	0.000000
Weight fraction/ %:	74.8(3)	Roughness:	0.000000
Space group (No.):	P 1 21/c 1 (14)	Fitting mode:	Structure Fit
Lattice parameters:		U Left:	0.021(8)
a/ A:	6.1078(2)	V Left:	-0.002(6)
b/ A:	6.1967(2)	W Left:	0.017(1)
c/ A:	6.1852(2)	Preferred orientation direction/ hkl:	0.00 0.00 1.00
alpha/ °:	90	Preferred orientation parameter:	1.000000
beta/ °:	118.588(2)	Asymmetry parameter 1:	0.11(2)
gamma/ °:	90	Asymmetry parameter 2:	0.000000
		Peak shape:	

parameter 1 Left:	0.59(1)	R (Bragg)/ %:	4.64032
parameter 2 Left:	0.000000		
parameter 3 Left:	0.000000		

Occupancy, atomic fract. coordinates and Biso for $LiYO_2$-LT, 50992-ICSD

Atom	Wyck.	s.o.f.	x	y	z	B/ 10^4 pm^2
Y1	4e	0.940000	0.266000	0.131200	0.020700	0.210000
Li1	4e	1.000000	0.791000	0.346000	0.068000	0.600000
O1	4e	1.000000	0.045900	0.107000	0.237800	0.280000
O2	4e	1.000000	0.512100	0.328800	0.343900	0.290000
Yb1	4e	0.050000	0.266000	0.131200	0.020700	0.210000
Er1	4e	0.010000	0.266000	0.131200	0.020700	0.210000

Relevant parameters of $LiYO_2$-HT, 50993-ICSD

Structure and profile data:	
Formula sum: $Y_{3.76}Li_{4.00}O_{8.00}Yb_{0.20}Er_{0.04}$	
Formula mass/ g/mol:	531.3438
Density (calculated)/ g/cm^3	4.3391
F(000):	239.3600
Weight fraction/ %:	12.2(2)
Space group (No.): I 41/a m d (141)	
Lattice parameters:	
a/ A:	4.4395(2)
b/ A:	4.4395(2)
c/ A:	10.3158(8)
alpha/ °:	90
beta/ °:	90
gamma/ °:	90
V/ 10^6 pm^3	203.31230
Overall displacement parameter:	0.000000
Extinction:	0.000000
Flat Plate Absorption Correction:	0.000000
Porosity:	0.000000
Roughness:	0.000000
Fitting mode:	Structure Fit
U Left:	0.068937
V Left:	-0.052995
W Left:	0.027085
Preferred orientation direction/ hkl:	0.00 0.00 1.00
Preferred orientation parameter:	1.000000
Asymmetry parameter 1:	0.170069
Asymmetry parameter 2:	0.000000
Peak shape:	
parameter 1 Left:	0.626711
parameter 2 Left:	0.000000
parameter 3 Left:	0.000000
R (Bragg)/ %:	4.88727

Occupancy, atomic fract. coordinates and Biso for $LiYO_2$-HT, 50993-ICSD

Atom	Wyck.	s.o.f.	x	y	z	B/ 10^4 pm^2
Y1	4b	0.940000	0.000000	0.250000	0.375000	0.000000
Li1	4b	0.000000	0.000000	0.250000	0.375000	0.000000
Li2	4a	1.000000	0.000000	0.750000	0.125000	0.000000
O1	8e	1.000000	0.000000	0.250000	0.152000	0.000000
Yb1	4b	0.050000	0.000000	0.250000	0.375000	0.000000
Er1	4b	0.010000	0.000000	0.250000	0.375000	0.000000

The data for the $LiYO_2$:5%Yb^{3+}, 1%Er^{3+} at 260 K:

Global Parameters

Number of used phases:	3
Number of variables:	25
Number of constraints:	2
Zero shift/ °2Theta:	0.000000
Specimen displacement/ mm :	0.233(1)
Profile function:	Pseudo Voigt
Background:	Polynomial
R (expected)/ %:	0.58992
R (profile)/ %:	3.44317
R (weighted profile)/ %:	4.81255
GOF:	66.55289
d-statistic:	0.23782
U standard:	0.000000
V standard:	0.000000
W standard:	0.010000
U Left:	0.000000
V Left:	0.000000
W Left:	0.010000
U Right:	0.000000
V Right:	0.000000
W Right:	0.010000
Asymmetry Type:	No Asymmetry
	Function
Asymmetry 1:	0.000000
Asymmetry 2:	0.000000
Shape Type:	Shape Individual
Shape 1 Left:	0.600000
Shape 2 Left:	0.000000
Shape 3 Left:	0.000000
Shape 1 Right:	0.600000
Shape 2 Right:	0.000000
Shape 3 Right:	0.000000
K a1/a2 intensity ratio:	0.500000
K alpha/beta intensity ratio:	0.000000
Crystal Shape Factor K:	1.0000
Instrumental FWHM Curve Type:	Caglioti function
Instr. Gauss Curve Coefficient A:	0.0045(5)
Instr. Gauss Curve Coefficient B:	-0.0032(9)
Instr. Gauss Curve Coefficient C:	0.0046(3)
Instr. Lorentz Curve Coefficient A:	0.0062(7)
Instr. Lorentz Curve Coefficient B:	-0.004(1)
Instr. Lorentz Curve Coefficient C:	0.0064(5)

Relevant parameters of $LiYO_2$-LT, 50992-ICSD

Structure and profile data:

Formula sum: $Y_{3.76}Li_{4.00}O_{8.00}Yb_{0.20}Er_{0.04}$

Formula mass/ g/mol:	531.3438	Porosity:	0.000000
Density (calculated)/ g/cm^3	4.2933	Roughness:	0.000000
F(000):	239.3600	Fitting mode:	Structure Fit
Weight fraction/ %:	53.3(3)	U Left:	0.08(1)
Space group (No.):	P 1 21/c 1 (14)	V Left:	-0.073(9)
Lattice parameters:		W Left:	0.035(2)
a/ A:	6.1056(2)	Preferred orientation direction/ hkl:	0.00 0.00 1.00
b/ A:	6.2006(2)	Preferred orientation parameter:	1.000000
c/ A:	6.1785(2)	Asymmetry parameter 1:	0.04(3)
alpha/ °:	90	Asymmetry parameter 2:	0.000000
beta/ °:	118.541(2)	Peak shape:	
gamma/ °:	90	parameter 1 Left:	0.41(2)
V/ 10^6 pm^3	205.48130	parameter 2 Left:	0.000000
Overall displacement parameter:	0.000000	parameter 3 Left:	0.000000
Extinction:	0.000000	R (Bragg)/ %:	4.70147
Flat Plate Absorption Correction:	0.000000		

Occupancy, atomic fract. coordinates and Biso for $LiYO_2$-LT, 50992-ICSD

Atom	Wyck.	s.o.f.	x	y	z	B/ 10^4 pm^2
Y1	4e	0.940000	0.266000	0.131200	0.020700	0.210000
Li1	4e	1.000000	0.791000	0.346000	0.068000	0.600000
O1	4e	1.000000	0.045900	0.107000	0.237800	0.280000
O2	4e	1.000000	0.512100	0.328800	0.343900	0.290000
Yb1	4e	0.050000	0.266000	0.131200	0.020700	0.210000
Er1	4e	0.010000	0.266000	0.131200	0.020700	0.210000

Relevant parameters of LiYO2-HT, 50993-ICSD

Structure and profile data:		Extinction:	0.000000
Formula sum: $Y_{3.76}Li_{4.00}O_{8.00}Yb_{0.20}Er_{0.04}$		Flat Plate Absorption Correction:	0.000000
Formula mass/ g/mol:	531.3438	Porosity:	0.000000
Density (calculated)/ g/cm^3	4.3365	Roughness:	0.000000
F(000):	239.3600	Fitting mode:	Structure Fit
Weight fraction/ %:	33.6(3)	U Left:	0.062(7)
Space group (No.): I 41/a m d (141)		V Left:	-0.051(6)
Lattice parameters:		W Left:	0.021(1)
a/ A:	4.43962(9)	Preferred orientation direction/ hkl:	0.00 0.00 1.00
b/ A:	4.43962(9)	Preferred orientation parameter:	1.000000
c/ A:	10.3213(3)	Asymmetry parameter 1:	0.170069
alpha/ °:	90	Asymmetry parameter 2:	0.000000
beta/ °:	90	Peak shape:	
gamma/ °:	90	parameter 1 Left:	0.76(3)
V/ 10^6 pm^3	203.43410	parameter 2 Left:	0.000000
Overall displacement parameter:	0.000000	parameter 3 Left:	0.000000
		R (Bragg)/ %:	4.53203

Occupancy, atomic fract. coordinates and Biso for $LiYO_2$-HT, 50993-ICSD

Atom	Wyck.	s.o.f.	x	y	z	B/ 10^4 pm^2
Y1	4b	0.940000	0.000000	0.250000	0.375000	0.000000
Li1	4b	0.000000	0.000000	0.250000	0.375000	0.000000
Li2	4a	1.000000	0.000000	0.750000	0.125000	0.000000
O1	8e	1.000000	0.000000	0.250000	0.152000	0.000000
Yb1	4b	0.050000	0.000000	0.250000	0.375000	0.000000
Er1	4b	0.010000	0.000000	0.250000	0.375000	0.000000

The data for the $LiYO_2$:5%Yb^{3+}, 1%Er^{3+} at 280 K:

Global Parameters

Number of used phases:	3	V standard:	0.000000
Number of variables:	16	W standard:	0.010000
Number of constraints:	2	U Left:	0.000000
Zero shift/ °2Theta:	0.000000	V Left:	0.000000
Specimen displacement/ mm :	0.2064(7)	W Left:	0.010000
Profile function:	Pseudo Voigt	U Right:	0.000000
Background:	Polynomial	V Right:	0.000000
R (expected)/ %:	0.59093	W Right:	0.010000
R (profile)/ %:	3.46121	Asymmetry Type:	No Asymmetry Function
R (weighted profile)/ %:	4.96787		
GOF:	70.67464	Asymmetry 1:	0.000000
d-statistic:	0.22149	Asymmetry 2:	0.000000
U standard:	0.000000	Shape Type:	Shape Individual

Shape 1 Left:	0.600000	Instrumental FWHM Curve Type:	Caglioti function
Shape 2 Left:	0.000000	Instr. Gauss Curve Coefficient A:	0.0045(5)
Shape 3 Left:	0.000000	Instr. Gauss Curve Coefficient B:	-0.0032(9)
Shape 1 Right:	0.600000	Instr. Gauss Curve Coefficient C:	0.0046(3)
Shape 2 Right:	0.000000	Instr. Lorentz Curve Coefficient A:	0.0062(7)
Shape 3 Right:	0.000000	Instr. Lorentz Curve Coefficient B:	-0.004(1)
K a1/a2 intensity ratio:	0.500000	Instr. Lorentz Curve Coefficient C:	0.0064(5)
K alpha/beta intensity ratio:	0.000000		
Crystal Shape Factor K:	1.0000		

Relevant parameters of $LiYO_2$-LT, 50992-ICSD

Structure and profile data:		Extinction:	0.000000
Formula sum: $Y_{3.76}Li_{4.00}O_{8.00}Yb_{0.20}Er_{0.04}$		Flat Plate Absorption Correction:	0.000000
Formula mass/ g/mol:	531.3438	Porosity:	0.000000
Density (calculated)/ g/cm^3	4.2932	Roughness:	0.000000
F(000):	239.3600	Fitting mode:	Structure Fit
Weight fraction/ %:	0.000000	U Left:	0.078837
Space group (No.):	P 1 21/c 1 (14)	V Left:	-0.072365
Lattice parameters:		W Left:	0.035028
a/ A:	6.105643	Preferred orientation direction/ hkl:	0.00 0.00 1.00
b/ A:	6.200608	Preferred orientation parameter:	1.000000
c/ A:	6.178551	Asymmetry parameter 1:	0.037058
alpha/ °:	90	Asymmetry parameter 2:	0.000000
beta/ °:	118.542100	Peak shape:	
gamma/ °:	90	parameter 1 Left:	0.401383
V/ 10^6 pm^3	205.48380	parameter 2 Left:	0.000000
Overall displacement parameter:	0.000000	parameter 3 Left:	0.000000
		R (Bragg)/ %:	999.00000

Occupancy, atomic fract. coordinates and Biso for $LiYO_2$-LT, 50992-ICSD

Atom	Wyck.	s.o.f.	x	y	z	B/ 10^4 pm^2
Y1	4e	0.940000	0.266000	0.131200	0.020700	0.210000
Li1	4e	1.000000	0.791000	0.346000	0.068000	0.600000
O1	4e	1.000000	0.045900	0.107000	0.237800	0.280000
O2	4e	1.000000	0.512100	0.328800	0.343900	0.290000
Yb1	4e	0.050000	0.266000	0.131200	0.020700	0.210000
Er1	4e	0.010000	0.266000	0.131200	0.020700	0.210000

Relevant parameters of $LiYO_2$-HT, 50993-ICSD

Structure and profile data:		Extinction:	0.000000
Formula sum: $Y_{3.76}Li_{4.00}O_{8.00}Yb_{0.20}Er_{0.04}$		Flat Plate Absorption Correction:	0.000000
Formula mass/ g/mol:	531.3438	Porosity:	0.000000
Density (calculated)/ g/cm^3	4.3318	Roughness:	0.000000
F(000):	239.3600	Fitting mode:	Structure Fit
Weight fraction/ %:	85.8(2)	U Left:	0.046(3)
Space group (No.): I 41/a m d (141)		V Left:	-0.047(2)
Lattice parameters:		W Left:	0.0227(5)
a/ A:	4.43984(4)	Preferred orientation direction/ hkl:	0.00 0.00 1.00
b/ A:	4.43984(4)	Preferred orientation parameter:	1.000000
c/ A:	10.3314(1)	Asymmetry parameter 1:	0.16(2)
alpha/ °:	90	Asymmetry parameter 2:	0.000000
beta/ °:	90	Peak shape:	
gamma/ °:	90	parameter 1 Left:	0.662(9)
V/ 10^6 pm^3	203.65490	parameter 2 Left:	0.000000
Overall displacement parameter:	0.000000	parameter 3 Left:	0.000000
		R (Bragg)/ %:	4.26433

Occupancy, atomic fract. coordinates and Biso for $LiYO_2$-HT, 50993-ICSD

Atom	Wyck.	s.o.f.	x	y	z	B/ 10^4 pm^2
Y1	4b	0.940000	0.000000	0.250000	0.375000	0.000000
Li1	4b	0.000000	0.000000	0.250000	0.375000	0.000000
Li2	4a	1.000000	0.000000	0.750000	0.125000	0.000000
O1	8e	1.000000	0.000000	0.250000	0.152000	0.000000
Yb1	4b	0.050000	0.000000	0.250000	0.375000	0.000000
Er1	4b	0.010000	0.000000	0.250000	0.375000	0.000000

The data for the $LiYO_2$:5%Yb^{3+}, 1%Er^{3+} at 298 K:

Global Parameters

Number of used phases:	3	Number of variables:	16

Number of constraints:	2		Function
Zero shift/ °2Theta:	0.000000	Asymmetry 1:	0.000000
Specimen displacement/ mm :	0.2055(7)	Asymmetry 2:	0.000000
Profile function:	Pseudo Voigt	Shape Type:	Shape Individual
Background:	Polynomial	Shape 1 Left:	0.600000
R (expected)/ %:	0.58691	Shape 2 Left:	0.000000
R (profile)/ %:	3.62472	Shape 3 Left:	0.000000
R (weighted profile)/ %:	5.54751	Shape 1 Right:	0.600000
GOF:	89.34216	Shape 2 Right:	0.000000
d-statistic:	0.18040	Shape 3 Right:	0.000000
U standard:	0.000000	K a1/a2 intensity ratio:	0.500000
V standard:	0.000000	K alpha/beta intensity ratio:	0.000000
W standard:	0.010000	Crystal Shape Factor K:	1.0000
U Left:	0.000000	Instrumental FWHM Curve Type:	Caglioti function
V Left:	0.000000	Instr. Gauss Curve Coefficient A:	0.0045(5)
W Left:	0.010000	Instr. Gauss Curve Coefficient B:	-0.0032(9)
U Right:	0.000000	Instr. Gauss Curve Coefficient C:	0.0046(3)
V Right:	0.000000	Instr. Lorentz Curve Coefficient A:	0.0062(7)
W Right:	0.010000	Instr. Lorentz Curve Coefficient B:	-0.004(1)
Asymmetry Type:	No Asymmetry	Instr. Lorentz Curve Coefficient C:	0.0064(5)

Relevant parameters of $LiYO_2$-LT, 50992-ICSD

Structure and profile data:		Extinction:	0.000000
Formula sum: $Y_{3.76}Li_{4.00}O_{8.00}Yb_{0.20}Er_{0.04}$		Flat Plate Absorption Correction:	0.000000
Formula mass/ g/mol:	531.3438	Porosity:	0.000000
Density (calculated)/ g/cm^3	4.2932	Roughness:	0.000000
F(000):	239.3600	Fitting mode:	Structure Fit
Weight fraction/ %:	0.000000	U Left:	0.078837
Space group (No.):	P 1 21/c 1 (14)	V Left:	-0.072365
Lattice parameters:		W Left:	0.035028
a/ A:	6.105643	Preferred orientation direction/ hkl:	0.00 0.00 1.00
b/ A:	6.200608	Preferred orientation parameter:	1.000000
c/ A:	6.178551	Asymmetry parameter 1:	0.037058
alpha/ °:	90	Asymmetry parameter 2:	0.000000
beta/ °:	118.542100	Peak shape:	
gamma/ °:	90	parameter 1 Left:	0.401383
V/ 10^6 pm^3	205.48380	parameter 2 Left:	0.000000
Overall displacement parameter:	0.000000	parameter 3 Left:	0.000000
		R (Bragg)/ %:	999.00000

Occupancy, atomic fract. coordinates and Biso for $LiYO_2$-LT, 50992-ICSD

Atom	Wyck.	s.o.f.	x	y	z	B/ 10^4 pm^2
Y1	4e	0.940000	0.266000	0.131200	0.020700	0.210000
Li1	4e	1.000000	0.791000	0.346000	0.068000	0.600000
O1	4e	1.000000	0.045900	0.107000	0.237800	0.280000
O2	4e	1.000000	0.512100	0.328800	0.343900	0.290000
Yb1	4e	0.050000	0.266000	0.131200	0.020700	0.210000
Er1	4e	0.010000	0.266000	0.131200	0.020700	0.210000

Relevant parameters of $LiYO_2$-HT, 50993-ICSD

Structure and profile data:		Extinction:	0.000000
Formula sum: $Y_{3.76}Li_{4.00}O_{8.00}Yb_{0.20}Er_{0.04}$		Flat Plate Absorption Correction:	0.000000
Formula mass/ g/mol:	531.3438	Porosity:	0.000000
Density (calculated)/ g/cm^3	4.3301	Roughness:	0.000000
F(000):	239.3600	Fitting mode:	Structure Fit
Weight fraction/ %:	86.3(2)	U Left:	0.046(3)
Space group (No.): I 41/a m d (141)		V Left:	-0.046(3)
Lattice parameters:		W Left:	0.0220(5)
a/ A:	4.44027(5)	Preferred orientation direction/ hkl:	0.00 0.00 1.00
b/ A:	4.44027(5)	Preferred orientation parameter:	1.000000
c/ A:	10.3335(1)	Asymmetry parameter 1:	0.20(2)
alpha/ °:	90	Asymmetry parameter 2:	0.000000
beta/ °:	90	Peak shape:	
gamma/ °:	90	parameter 1 Left:	0.555(9)
V/ 10^6 pm^3	203.73570	parameter 2 Left:	0.000000
Overall displacement parameter:	0.000000	parameter 3 Left:	0.000000
		R (Bragg)/ %:	5.33567

Occupancy, atomic fract. coordinates and Biso for $LiYO_2$-HT, 50993-ICSD

Atom	Wyck.	s.o.f.	x	y	z	B/ 10^4 pm^2
Y1	4b	0.940000	0.000000	0.250000	0.375000	0.000000

Li1	4b	0.000000	0.000000	0.250000	0.375000	0.000000
Li2	4a	1.000000	0.000000	0.750000	0.125000	0.000000
O1	8e	1.000000	0.000000	0.250000	0.152000	0.000000
Yb1	4b	0.050000	0.000000	0.250000	0.375000	0.000000
Er1	4b	0.010000	0.000000	0.250000	0.375000	0.000000

The data for the $LiYO_2$:10%Yb^{3+}, 1%Er^{3+} at 100 K:

Global Parameters

Number of used phases:	3		Function
Number of variables:	18	Asymmetry 1:	0.000000
Number of constraints:	1	Asymmetry 2:	0.000000
Zero shift/ °2Theta:	0.000000	Shape Type:	Shape Individual
Specimen displacement/ mm :	0.210(2)	Shape 1 Left:	0.600000
Profile function:	Pseudo Voigt	Shape 2 Left:	0.000000
Background:	Polynomial	Shape 3 Left:	0.000000
R (expected)/ %:	0.58987	Shape 1 Right:	0.600000
R (profile)/ %:	4.28735	Shape 2 Right:	0.000000
R (weighted profile)/ %:	6.20166	Shape 3 Right:	0.000000
GOF:	110.53670	K a1/a2 intensity ratio:	0.500000
d-statistic:	0.12153	K alpha/beta intensity ratio:	0.000000
U standard:	0.000000	Crystal Shape Factor K:	1.0000
V standard:	0.000000	Instrumental FWHM Curve Type:	Caglioti function
W standard:	0.010000	Instr. Gauss Curve Coefficient A:	0.0045(5)
U Left:	0.000000	Instr. Gauss Curve Coefficient B:	-0.0032(9)
V Left:	0.000000	Instr. Gauss Curve Coefficient C:	0.0046(3)
W Left:	0.010000	Instr. Lorentz Curve Coefficient A:	0.0062(7)
U Right:	0.000000	Instr. Lorentz Curve Coefficient B:	-0.004(1)
V Right:	0.000000	Instr. Lorentz Curve Coefficient C:	0.0064(5)
W Right:	0.010000		
Asymmetry Type:	No Asymmetry		

Relevant parameters of $LiYO_2$-LT, 50992-ICSD

Structure and profile data:		Extinction:	0.000000
Formula sum: $Y_{3.56}Li_{4.00}O_{8.00}Yb_{0.40}Er_{0.04}$		Flat Plate Absorption Correction:	0.000000
Formula mass/ g/mol:	548.1706	Porosity:	0.000000
Density (calculated)/ g/cm^3	4.4411	Roughness:	0.000000
F(000):	245.5600	Fitting mode:	Structure Fit
Weight fraction/ %:	92.3(3)	U Left:	0.14(2)
Space group (No.):	P 1 21/c 1 (14)	V Left:	-0.04(1)
Lattice parameters:		W Left:	0.029(2)
a/ A:	6.1097(2)	Preferred orientation direction/ hkl:	0.00 0.00 1.00
b/ A:	6.1766(2)	Preferred orientation parameter:	1.000000
c/ A:	6.1930(2)	Asymmetry parameter 1:	0.07(2)
alpha/ °:	90	Asymmetry parameter 2:	0.000000
beta/ °:	118.731(2)	Peak shape:	
gamma/ °:	90	parameter 1 Left:	0.49(1)
V/ 10^6 pm^3	204.93270	parameter 2 Left:	0.000000
Overall displacement parameter:	0.000000	parameter 3 Left:	0.000000
		R (Bragg)/ %:	5.95958

Occupancy, atomic fract. coordinates and Biso for $LiYO_2$-LT, 50992-ICSD

Atom	Wyck.	s.o.f.	x	y	z	B/ 10^4 pm^2
Y1	4e	0.890000	0.266000	0.131200	0.020700	0.210000
Li1	4e	1.000000	0.791000	0.346000	0.068000	0.600000
O1	4e	1.000000	0.045900	0.107000	0.237800	0.280000
O2	4e	1.000000	0.512100	0.328800	0.343900	0.290000
Yb1	4e	0.100000	0.266000	0.131200	0.020700	0.210000
Er1	4e	0.010000	0.266000	0.131200	0.020700	0.210000

Relevant parameters of $LiYO_2$-HT, 50993-ICSD

Structure and profile data:		b/ A:	4.437816
Formula sum: $Y_{3.56}Li_{4.00}O_{8.00}Yb_{0.40}Er_{0.04}$		c/ A:	10.288340
Formula mass/ g/mol:	548.1706	alpha/ °:	90
Density (calculated)/ g/cm^3	4.4918	beta/ °:	90
F(000):	245.5600	gamma/ °:	90
Weight fraction/ %:	0.000000	V/ 10^6 pm^3	202.62080
Space group (No.): I 41/a m d (141)		Overall displacement parameter:	0.000000
Lattice parameters:		Extinction:	0.000000
a/ A:	4.437816	Flat Plate Absorption Correction:	0.000000

Porosity:	0.000000	Asymmetry parameter 1:	0.170069
Roughness:	0.000000	Asymmetry parameter 2:	0.000000
Fitting mode:	Structure Fit	Peak shape:	
U Left:	0.068937	parameter 1 Left:	0.626711
V Left:	-0.052995	parameter 2 Left:	0.000000
W Left:	0.027085	parameter 3 Left:	0.000000
Preferred orientation direction/ hkl:	0.00 0.00 1.00	R (Bragg)/ %:	999.00000
Preferred orientation parameter:	1.000000		

Occupancy, atomic fract. coordinates and Biso for $LiYO_2$-HT, 50993-ICSD

Atom	Wyck.	s.o.f.	x	y	z	B/ 10^4 pm^2
Y1	4b	0.890000	0.000000	0.250000	0.375000	0.000000
Li1	4b	0.000000	0.000000	0.250000	0.375000	0.000000
Li2	4a	1.000000	0.000000	0.750000	0.125000	0.000000
O1	8e	1.000000	0.000000	0.250000	0.152000	0.000000
Yb1	4b	0.100000	0.000000	0.250000	0.375000	0.000000
Er1	4b	0.010000	0.000000	0.250000	0.375000	0.000000

The data for the $LiYO_2$:10%Yb^{3+}, 1%Er^{3+} at 120 K:

Global Parameters

Number of used phases:	3	Asymmetry Type:	No Asymmetry Function
Number of variables:	20		
Number of constraints:	2	Asymmetry 1:	0.000000
Zero shift/ °2Theta:	0.000000	Asymmetry 2:	0.000000
Specimen displacement/ mm :	0.207(2)	Shape Type:	Shape Individual
Profile function:	Pseudo Voigt	Shape 1 Left:	0.600000
Background:	Polynomial	Shape 2 Left:	0.000000
R (expected)/ %:	0.59040	Shape 3 Left:	0.000000
R (profile)/ %:	4.30382	Shape 1 Right:	0.600000
R (weighted profile)/ %:	6.23410	Shape 2 Right:	0.000000
GOF:	111.49600	Shape 3 Right:	0.000000
d-statistic:	0.12035	K a1/a2 intensity ratio:	0.500000
U standard:	0.000000	K alpha/beta intensity ratio:	0.000000
V standard:	0.000000	Crystal Shape Factor K:	1.0000
W standard:	0.010000	Instrumental FWHM Curve Type:	Caglioti function
U Left:	0.000000	Instr. Gauss Curve Coefficient A:	0.0045(5)
V Left:	0.000000	Instr. Gauss Curve Coefficient B:	-0.0032(9)
W Left:	0.010000	Instr. Gauss Curve Coefficient C:	0.0046(3)
U Right:	0.000000	Instr. Lorentz Curve Coefficient A:	0.0062(7)
V Right:	0.000000	Instr. Lorentz Curve Coefficient B:	-0.004(1)
W Right:	0.010000	Instr. Lorentz Curve Coefficient C:	0.0064(5)

Relevant parameters of $LiYO_2$-LT, 50992-ICSD

Structure and profile data:		Flat Plate Absorption Correction:	0.000000
Formula sum: $Y_{3.56}Li_{4.00}O_{8.00}Yb_{0.40}Er_{0.04}$		Porosity:	0.000000
Formula mass/ g/mol:	548.1706	Roughness:	0.000000
Density (calculated)/ g/cm^3	4.4422	Fitting mode:	Structure Fit
F(000):	245.5600	U Left:	0.15(2)
Weight fraction/ %:	92.5(3)	V Left:	-0.04(2)
Space group (No.):	P 1 21/c 1 (14)	W Left:	0.029(2)
Lattice parameters:		Preferred orientation direction/ hkl:	0.00 0.00 1.00
a/ A:	6.1076(2)	Preferred orientation parameter:	1.000000
b/ A:	6.1784(3)	Asymmetry parameter 1:	0.07(2)
c/ A:	6.1903(2)	Asymmetry parameter 2:	0.000000
alpha/ °:	90	Peak shape:	
beta/ °:	118.706(2)	parameter 1 Left:	0.47(1)
gamma/ °:	90	parameter 2 Left:	0.000000
V/ 10^6 pm^3	204.88310	parameter 3 Left:	0.000000
Overall displacement parameter:	0.000000	R (Bragg)/ %:	5.95607
Extinction:	0.000000		

Occupancy, atomic fract. coordinates and Biso for $LiYO_2$-LT, 50992-ICSD

Atom	Wyck.	s.o.f.	x	y	z	B/ 10^4 pm^2
Y1	4e	0.890000	0.266000	0.131200	0.020700	0.210000
Li1	4e	1.000000	0.791000	0.346000	0.068000	0.600000
O1	4e	1.000000	0.045900	0.107000	0.237800	0.280000
O2	4e	1.000000	0.512100	0.328800	0.343900	0.290000
Yb1	4e	0.100000	0.266000	0.131200	0.020700	0.210000
Er1	4e	0.010000	0.266000	0.131200	0.020700	0.210000

Relevant parameters of $LiYO_2$-HT, 50993-ICSD

Structure and profile data:	
Formula sum: $Y_{3.56}Li_{4.00}O_{8.00}Yb_{0.40}Er_{0.04}$	
Formula mass/ g/mol:	548.1706
Density (calculated)/ g/cm^3	4.4918
F(000):	245.5600
Weight fraction/ %:	0.000000
Space group (No.): I 41/a m d (141)	
Lattice parameters:	
a/ A: 4(461431898112)	
b/ A: 4(461431898112)	
c/ A: 10(5749574991872)	
alpha/ °:	90
beta/ °:	90
gamma/ °:	90
V/ 10^6 pm^3	202.62080
Overall displacement parameter:	0.000000
Extinction:	0.000000
Flat Plate Absorption Correction:	0.000000
Porosity:	0.000000
Roughness:	0.000000
Fitting mode:	Structure Fit
U Left:	0.068937
V Left:	-0.052995
W Left:	0.027085
Preferred orientation direction/ hkl:	0.00 0.00 1.00
Preferred orientation parameter:	1.000000
Asymmetry parameter 1:	0.170069
Asymmetry parameter 2:	0.000000
Peak shape:	
parameter 1 Left:	0.626711
parameter 2 Left:	0.000000
parameter 3 Left:	0.000000
R (Bragg)/ %:	999.00000

Occupancy, atomic fract. coordinates and Biso for $LiYO_2$-HT, 50993-ICSD

Atom	Wyck.	s.o.f.	x	y	z	B/ 10^4 pm^2
Y1	4b	0.890000	0.000000	0.250000	0.375000	0.000000
Li1	4b	0.000000	0.000000	0.250000	0.375000	0.000000
Li2	4a	1.000000	0.000000	0.750000	0.125000	0.000000
O1	8e	1.000000	0.000000	0.250000	0.152000	0.000000
Yb1	4b	0.100000	0.000000	0.250000	0.375000	0.000000
Er1	4b	0.010000	0.000000	0.250000	0.375000	0.000000

The data for the $LiYO_2$:10%Yb^{3+}, 1%Er^{3+} at 140 K:

Global Parameters

Number of used phases:	3
Number of variables:	20
Number of constraints:	2
Zero shift/ °2Theta:	0.000000
Specimen displacement/ mm :	0.203(2)
Profile function:	Pseudo Voigt
Background:	Polynomial
R (expected)/ %:	0.59093
R (profile)/ %:	4.38514
R (weighted profile)/ %:	6.40270
GOF:	117.39760
d-statistic:	0.11443
U standard:	0.000000
V standard:	0.000000
W standard:	0.010000
U Left:	0.000000
V Left:	0.000000
W Left:	0.010000
U Right:	0.000000
V Right:	0.000000
W Right:	0.010000
Asymmetry Type:	No Asymmetry
	Function
Asymmetry 1:	0.000000
Asymmetry 2:	0.000000
Shape Type:	Shape Individual
Shape 1 Left:	0.600000
Shape 2 Left:	0.000000
Shape 3 Left:	0.000000
Shape 1 Right:	0.600000
Shape 2 Right:	0.000000
Shape 3 Right:	0.000000
K a1/a2 intensity ratio:	0.500000
K alpha/beta intensity ratio:	0.000000
Crystal Shape Factor K:	1.0000
Instrumental FWHM Curve Type:	Caglioti function
Instr. Gauss Curve Coefficient A:	0.0045(5)
Instr. Gauss Curve Coefficient B:	-0.0032(9)
Instr. Gauss Curve Coefficient C:	0.0046(3)
Instr. Lorentz Curve Coefficient A:	0.0062(7)
Instr. Lorentz Curve Coefficient B:	-0.004(1)
Instr. Lorentz Curve Coefficient C:	0.0064(5)

Relevant parameters of $LiYO_2$-LT, 50992-ICSD

Structure and profile data:	
Formula sum:	$Y_{3.56}Li_{4.00}O_{8.00}Yb_{0.40}Er_{0.04}$
Formula mass/ g/mol:	548.1706
Density (calculated)/ g/cm^3	4.4431
F(000):	245.5600
Weight fraction/ %:	92.4(3)
Space group (No.):	P 1 21/c 1 (14)
Lattice parameters:	
a/ A:	6.1056(2)
b/ A:	6.1808(3)
c/ A:	6.1873(2)
alpha/ °:	90
beta/ °:	118.680(2)
gamma/ °:	90
V/ 10^6 pm^3	204.84320
Overall displacement parameter:	0.000000
Extinction:	0.000000
Flat Plate Absorption Correction:	0.000000
Porosity:	0.000000
Roughness:	0.000000
Fitting mode:	Structure Fit
U Left:	0.13(2)
V Left:	-0.02(2)
W Left:	0.027(2)
Preferred orientation direction/ hkl:	0.00 0.00 1.00
Preferred orientation parameter:	1.000000
Asymmetry parameter 1:	0.06(2)

Asymmetry parameter 2:	0.000000	parameter 2 Left:	0.000000
Peak shape:		parameter 3 Left:	0.000000
parameter 1 Left:	0.47(1)	R (Bragg)/ %:	6.29724

Occupancy, atomic fract. coordinates and Biso for $LiYO_2$-LT, 50992-ICSD

Atom	Wyck.	s.o.f.	x	y	z	B/ 10^4 pm^2
Y1	4e	0.890000	0.266000	0.131200	0.020700	0.210000
Li1	4e	1.000000	0.791000	0.346000	0.068000	0.600000
O1	4e	1.000000	0.045900	0.107000	0.237800	0.280000
O2	4e	1.000000	0.512100	0.328800	0.343900	0.290000
Yb1	4e	0.100000	0.266000	0.131200	0.020700	0.210000
Er1	4e	0.010000	0.266000	0.131200	0.020700	0.210000

Relevant parameters of $LiYO_2$-HT, 50993-ICSD

Structure and profile data:		Extinction:	0.000000
Formula sum: $Y_{3.56}Li_{4.00}O_{8.00}Yb_{0.40}Er_{0.04}$		Flat Plate Absorption Correction:	0.000000
Formula mass/ g/mol:	548.1706	Porosity:	0.000000
Density (calculated)/ g/cm^3	4.4918	Roughness:	0.000000
F(000):	245.5600	Fitting mode:	Structure Fit
Weight fraction/ %:	0.000000	U Left:	0.068937
Space group (No.): I 41/a m d (141)		V Left:	-0.052995
Lattice parameters:		W Left:	0.027085
a/ A: 4(473486393344)		Preferred orientation direction/ hkl:	0.00 0.00 1.00
b/ A: 4(473486393344)		Preferred orientation parameter:	1.000000
c/ A: 10(5899777212416)		Asymmetry parameter 1:	0.170069
alpha/ °:	90	Asymmetry parameter 2:	0.000000
beta/ °:	90	Peak shape:	
gamma/ °:	90	parameter 1 Left:	0.626711
V/ 10^6 pm^3	202.62080	parameter 2 Left:	0.000000
Overall displacement parameter:	0.000000	parameter 3 Left:	0.000000
		R (Bragg)/ %:	999.00000

Occupancy, atomic fract. coordinates and Biso for $LiYO_2$-HT, 50993-ICSD

Atom	Wyck.	s.o.f.	x	y	z	B/ 10^4 pm^2
Y1	4b	0.890000	0.000000	0.250000	0.375000	0.000000
Li1	4b	0.000000	0.000000	0.250000	0.375000	0.000000
Li2	4a	1.000000	0.000000	0.750000	0.125000	0.000000
O1	8e	1.000000	0.000000	0.250000	0.152000	0.000000
Yb1	4b	0.100000	0.000000	0.250000	0.375000	0.000000
Er1	4b	0.010000	0.000000	0.250000	0.375000	0.000000

The data for the $LiYO_2$:10%Yb^{3+}, 1%Er^{3+} at 160 K:

Global Parameters

Number of used phases:	3		Function
Number of variables:	21	Asymmetry 1:	0.000000
Number of constraints:	2	Asymmetry 2:	0.000000
Zero shift/ °2Theta:	0.000000	Shape Type:	Shape Individual
Specimen displacement/ mm :	0.198(2)	Shape 1 Left:	0.600000
Profile function:	Pseudo Voigt	Shape 2 Left:	0.000000
Background:	Polynomial	Shape 3 Left:	0.000000
R (expected)/ %:	0.59075	Shape 1 Right:	0.600000
R (profile)/ %:	4.22539	Shape 2 Right:	0.000000
R (weighted profile)/ %:	6.11096	Shape 3 Right:	0.000000
GOF:	107.00730	K a1/a2 intensity ratio:	0.500000
d-statistic:	0.11776	K alpha/beta intensity ratio:	0.000000
U standard:	0.000000	Crystal Shape Factor K:	1.0000
V standard:	0.000000	Instrumental FWHM Curve Type:	Caglioti function
W standard:	0.010000	Instr. Gauss Curve Coefficient A:	0.0045(5)
U Left:	0.000000	Instr. Gauss Curve Coefficient B:	-0.0032(9)
V Left:	0.000000	Instr. Gauss Curve Coefficient C:	0.0046(3)
W Left:	0.010000	Instr. Lorentz Curve Coefficient A:	0.0062(7)
U Right:	0.000000	Instr. Lorentz Curve Coefficient B:	-0.004(1)
V Right:	0.000000	Instr. Lorentz Curve Coefficient C:	0.0064(5)
W Right:	0.010000		
Asymmetry Type:	No Asymmetry		

Relevant parameters of $LiYO_2$-LT, 50992-ICSD

Structure and profile data:		Formula mass/ g/mol:	548.1706
Formula sum: $Y_{3.56}Li_{4.00}O_{8.00}Yb_{0.40}Er_{0.04}$		Density (calculated)/ g/cm^3	4.4437
		F(000):	245.5600

Weight fraction/ %:	85.7(3)	Roughness:	0.000000
Space group (No.):	P 1 21/c 1 (14)	Fitting mode:	Structure Fit
Lattice parameters:		U Left:	0.08(2)
a/ A:	6.1037(2)	V Left:	-0.01(1)
b/ A:	6.1821(2)	W Left:	0.025(2)
c/ A:	6.1848(2)	Preferred orientation direction/ hkl:	0.00 0.00 1.00
alpha/ °:	90	Preferred orientation parameter:	1.000000
beta/ °:	118.645(2)	Asymmetry parameter 1:	0.09(2)
gamma/ °:	90	Asymmetry parameter 2:	0.000000
V/ 10^6 pm^3	204.81420	Peak shape:	
Overall displacement parameter:	0.000000	parameter 1 Left:	0.50(1)
Extinction:	0.000000	parameter 2 Left:	0.000000
Flat Plate Absorption Correction:	0.000000	parameter 3 Left:	0.000000
Porosity:	0.000000	R (Bragg)/ %:	6.18401

Occupancy, atomic fract. coordinates and Biso for $LiYO_2$-LT, 50992-ICSD

Atom	Wyck.	s.o.f.	x	y	z	B/ 10^4 pm^2
Y1	4e	0.890000	0.266000	0.131200	0.020700	0.210000
Li1	4e	1.000000	0.791000	0.346000	0.068000	0.600000
O1	4e	1.000000	0.045900	0.107000	0.237800	0.280000
O2	4e	1.000000	0.512100	0.328800	0.343900	0.290000
Yb1	4e	0.100000	0.266000	0.131200	0.020700	0.210000
Er1	4e	0.010000	0.266000	0.131200	0.020700	0.210000

Relevant parameters of $LiYO_2$-HT, 50993-ICSD

Structure and profile data:		Extinction:	0.000000
Formula sum: $Y_{3.56}Li_{4.00}O_{8.00}Yb_{0.40}Er_{0.04}$		Flat Plate Absorption Correction:	0.000000
Formula mass/ g/mol:	548.1706	Porosity:	0.000000
Density (calculated)/ g/cm^3	4.5153	Roughness:	0.000000
F(000):	245.5600	Fitting mode:	Structure Fit
Weight fraction/ %:	6.9(2)	U Left:	0.068937
Space group (No.): I 41/a m d (141)		V Left:	-0.052995
Lattice parameters:		W Left:	0.027085
a/ A:	4.4272(4)	Preferred orientation direction/ hkl:	0.00 0.00 1.00
b/ A:	4.4272(4)	Preferred orientation parameter:	1.000000
c/ A:	10.284(1)	Asymmetry parameter 1:	0.170069
alpha/ °:	90	Asymmetry parameter 2:	0.000000
beta/ °:	90	Peak shape:	
gamma/ °:	90	parameter 1 Left:	0.626711
V/ 10^6 pm^3	201.56540	parameter 2 Left:	0.000000
Overall displacement parameter:	0.000000	parameter 3 Left:	0.000000
		R (Bragg)/ %:	5.43740

Occupancy, atomic fract. coordinates and Biso for $LiYO_2$-HT, 50993-ICSD

Atom	Wyck.	s.o.f.	x	y	z	B/ 10^4 pm^2
Y1	4b	0.890000	0.000000	0.250000	0.375000	0.000000
Li1	4b	0.000000	0.000000	0.250000	0.375000	0.000000
Li2	4a	1.000000	0.000000	0.750000	0.125000	0.000000
O1	8e	1.000000	0.000000	0.250000	0.152000	0.000000
Yb1	4b	0.100000	0.000000	0.250000	0.375000	0.000000
Er1	4b	0.010000	0.000000	0.250000	0.375000	0.000000

The data for the $LiYO_2$:10%Yb^{3+}, 1%Er^{3+} at 170 K:

Global Parameters

Number of used phases:	3	V Left:	0.000000
Number of variables:	21	W Left:	0.010000
Number of constraints:	2	U Right:	0.000000
Zero shift/ °2Theta:	0.000000	V Right:	0.000000
Specimen displacement/ mm :	0.193(2)	W Right:	0.010000
Profile function:	Pseudo Voigt	Asymmetry Type:	No Asymmetry Function
Background:	Polynomial		
R (expected)/ %:	0.59089	Asymmetry 1:	0.000000
R (profile)/ %:	4.22703	Asymmetry 2:	0.000000
R (weighted profile)/ %:	6.12393	Shape Type:	Shape Individual
GOF:	107.41120	Shape 1 Left:	0.600000
d-statistic:	0.12548	Shape 2 Left:	0.000000
U standard:	0.000000	Shape 3 Left:	0.000000
V standard:	0.000000	Shape 1 Right:	0.600000
W standard:	0.010000	Shape 2 Right:	0.000000
U Left:	0.000000	Shape 3 Right:	0.000000

K a1/a2 intensity ratio:	0.500000	Instr. Gauss Curve Coefficient B:	-0.0032(9)
K alpha/beta intensity ratio:	0.000000	Instr. Gauss Curve Coefficient C:	0.0046(3)
Crystal Shape Factor K:	1.0000	Instr. Lorentz Curve Coefficient A:	0.0062(7)
Instrumental FWHM Curve Type:	Caglioti function	Instr. Lorentz Curve Coefficient B:	-0.004(1)
Instr. Gauss Curve Coefficient A:	0.0045(5)	Instr. Lorentz Curve Coefficient C:	0.0064(5)

Relevant parameters of $LiYO_2$-LT, 50992-ICSD

Structure and profile data:		Extinction:	0.000000
Formula sum: $Y_{3.56}Li_{4.00}O_{8.00}Yb_{0.40}Er_{0.04}$		Flat Plate Absorption Correction:	0.000000
Formula mass/ g/mol:	548.1706	Porosity:	0.000000
Density (calculated)/ g/cm^3	4.4438	Roughness:	0.000000
F(000):	245.5600	Fitting mode:	Structure Fit
Weight fraction/ %:	84.1(3)	U Left:	0.07(2)
Space group (No.):	P 1 21/c 1 (14)	V Left:	-0.02(1)
Lattice parameters:		W Left:	0.026(2)
a/ A:	6.1028(2)	Preferred orientation direction/ hkl:	0.00 0.00 1.00
b/ A:	6.1837(2)	Preferred orientation parameter:	1.000000
c/ A:	6.1831(2)	Asymmetry parameter 1:	0.07(2)
alpha/ °:	90	Asymmetry parameter 2:	0.000000
beta/ °:	118.630(2)	Peak shape:	
gamma/ °:	90	parameter 1 Left:	0.51(1)
V/ 10^6 pm^3	204.80720	parameter 2 Left:	0.000000
Overall displacement parameter:	0.000000	parameter 3 Left:	0.000000
		R (Bragg)/ %:	6.21808

Occupancy, atomic fract. coordinates and Biso for $LiYO_2$-LT, 50992-ICSD

Atom	Wyck.	s.o.f.	x	y	z	B/ 10^4 pm^2
Y1	4e	0.890000	0.266000	0.131200	0.020700	0.210000
Li1	4e	1.000000	0.791000	0.346000	0.068000	0.600000
O1	4e	1.000000	0.045900	0.107000	0.237800	0.280000
O2	4e	1.000000	0.512100	0.328800	0.343900	0.290000
Yb1	4e	0.100000	0.266000	0.131200	0.020700	0.210000
Er1	4e	0.010000	0.266000	0.131200	0.020700	0.210000

Relevant parameters of $LiYO_2$-HT, 50993-ICSD

Structure and profile data:		Extinction:	0.000000
Formula sum: $Y_{3.56}Li_{4.00}O_{8.00}Yb_{0.40}Er_{0.04}$		Flat Plate Absorption Correction:	0.000000
Formula mass/ g/mol:	548.1706	Porosity:	0.000000
Density (calculated)/ g/cm^3	4.5091	Roughness:	0.000000
F(000):	245.5600	Fitting mode:	Structure Fit
Weight fraction/ %:	8.7(2)	U Left:	0.068937
Space group (No.): I 41/a m d (141)		V Left:	-0.052995
Lattice parameters:		W Left:	0.027085
a/ A:	4.4319(3)	Preferred orientation direction/ hkl:	0.00 0.00 1.00
b/ A:	4.4319(3)	Preferred orientation parameter:	1.000000
c/ A:	10.276(1)	Asymmetry parameter 1:	0.170069
alpha/ °:	90	Asymmetry parameter 2:	0.000000
beta/ °:	90	Peak shape:	
gamma/ °:	90	parameter 1 Left:	0.626711
V/ 10^6 pm^3	201.84460	parameter 2 Left:	0.000000
Overall displacement parameter:	0.000000	parameter 3 Left:	0.000000
		R (Bragg)/ %:	5.13472

Occupancy, atomic fract. coordinates and Biso for $LiYO_2$-HT, 50993-ICSD

Atom	Wyck.	s.o.f.	x	y	z	B/ 10^4 pm^2
Y1	4b	0.890000	0.000000	0.250000	0.375000	0.000000
Li1	4b	0.000000	0.000000	0.250000	0.375000	0.000000
Li2	4a	1.000000	0.000000	0.750000	0.125000	0.000000
O1	8e	1.000000	0.000000	0.250000	0.152000	0.000000
Yb1	4b	0.100000	0.000000	0.250000	0.375000	0.000000
Er1	4b	0.010000	0.000000	0.250000	0.375000	0.000000

The data for the $LiYO_2$:10%Yb^{3+}, 1%Er^{3+} at 180 K:

Global Parameters

Number of used phases:	3	Profile function:	Pseudo Voigt
Number of variables:	21	Background:	Polynomial
Number of constraints:	2	R (expected)/ %:	0.59016
Zero shift/ °2Theta:	0.000000	R (profile)/ %:	4.07370
Specimen displacement/ mm :	0.188(1)	R (weighted profile)/ %:	5.90325

GOF:	100.05460	Shape 1 Left:	0.600000
d-statistic:	0.13433	Shape 2 Left:	0.000000
U standard:	0.000000	Shape 3 Left:	0.000000
V standard:	0.000000	Shape 1 Right:	0.600000
W standard:	0.010000	Shape 2 Right:	0.000000
U Left:	0.000000	Shape 3 Right:	0.000000
V Left:	0.000000	K a1/a2 intensity ratio:	0.500000
W Left:	0.010000	K alpha/beta intensity ratio:	0.000000
U Right:	0.000000	Crystal Shape Factor K:	1.0000
V Right:	0.000000	Instrumental FWHM Curve Type:	Caglioti function
W Right:	0.010000	Instr. Gauss Curve Coefficient A:	0.0045(5)
Asymmetry Type:	No Asymmetry Function	Instr. Gauss Curve Coefficient B:	-0.0032(9)
		Instr. Gauss Curve Coefficient C:	0.0046(3)
Asymmetry 1:	0.000000	Instr. Lorentz Curve Coefficient A:	0.0062(7)
Asymmetry 2:	0.000000	Instr. Lorentz Curve Coefficient B:	-0.004(1)
Shape Type:	Shape Individual	Instr. Lorentz Curve Coefficient C:	0.0064(5)

Relevant parameters of $LiYO_2$-LT, 50992-ICSD

Structure and profile data:		Flat Plate Absorption Correction:	0.000000
Formula sum: $Y_{3.56}Li_{4.00}O_{8.00}Yb_{0.40}Er_{0.04}$		Porosity:	0.000000
Formula mass/ g/mol:	548.1706	Roughness:	0.000000
Density (calculated)/ g/cm^3	4.4440	Fitting mode:	Structure Fit
F(000):	245.5600	U Left:	0.10(2)
Weight fraction/ %:	81.8(3)	V Left:	-0.04(1)
Space group (No.):	P 1 21/c 1 (14)	W Left:	0.030(2)
Lattice parameters:		Preferred orientation direction/ hkl:	0.00 0.00 1.00
a/ A:	6.1017(2)	Preferred orientation parameter:	1.000000
b/ A:	6.1856(2)	Asymmetry parameter 1:	0.11(2)
c/ A:	6.1811(2)	Asymmetry parameter 2:	0.000000
alpha/ °:	90	Peak shape:	
beta/ °:	118.615(2)	parameter 1 Left:	0.49(1)
gamma/ °:	90	parameter 2 Left:	0.000000
V/ 10^6 pm^3	204.79760	parameter 3 Left:	0.000000
Overall displacement parameter:	0.000000	R (Bragg)/ %:	6.06398
Extinction:	0.000000		

Occupancy, atomic fract. coordinates and Biso for $LiYO_2$-LT, 50992-ICSD

Atom	Wyck.	s.o.f.	x	y	z	B/ 10^4 pm^2
Y1	4e	0.890000	0.266000	0.131200	0.020700	0.210000
Li1	4e	1.000000	0.791000	0.346000	0.068000	0.600000
O1	4e	1.000000	0.045900	0.107000	0.237800	0.280000
O2	4e	1.000000	0.512100	0.328800	0.343900	0.290000
Yb1	4e	0.100000	0.266000	0.131200	0.020700	0.210000
Er1	4e	0.010000	0.266000	0.131200	0.020700	0.210000

Relevant parameters of $LiYO_2$-HT, 50993-ICSD

Structure and profile data:		Extinction:	0.000000
Formula sum: $Y_{3.56}Li_{4.00}O_{8.00}Yb_{0.40}Er_{0.04}$		Flat Plate Absorption Correction:	0.000000
Formula mass/ g/mol:	548.1706	Porosity:	0.000000
Density (calculated)/ g/cm^3	4.5051	Roughness:	0.000000
F(000):	245.5600	Fitting mode:	Structure Fit
Weight fraction/ %:	10.9(2)	U Left:	0.068937
Space group (No.): I 41/a m d (141)		V Left:	-0.052995
Lattice parameters:		W Left:	0.027085
a/ A:	4.4344(2)	Preferred orientation direction/ hkl:	0.00 0.00 1.00
b/ A:	4.4344(2)	Preferred orientation parameter:	1.000000
c/ A:	10.2737(8)	Asymmetry parameter 1:	0.170069
alpha/ °:	90	Asymmetry parameter 2:	0.000000
beta/ °:	90	Peak shape:	
gamma/ °:	90	parameter 1 Left:	0.626711
V/ 10^6 pm^3	202.02090	parameter 2 Left:	0.000000
Overall displacement parameter:	0.000000	parameter 3 Left:	0.000000
		R (Bragg)/ %:	4.92864

Occupancy, atomic fract. coordinates and Biso for $LiYO_2$-HT, 50993-ICSD

Atom	Wyck.	s.o.f.	x	y	z	B/ 10^4 pm^2
Y1	4b	0.890000	0.000000	0.250000	0.375000	0.000000
Li1	4b	0.000000	0.000000	0.250000	0.375000	0.000000
Li2	4a	1.000000	0.000000	0.750000	0.125000	0.000000
O1	8e	1.000000	0.000000	0.250000	0.152000	0.000000
Yb1	4b	0.100000	0.000000	0.250000	0.375000	0.000000

Er1	4b	0.010000	0.000000	0.250000	0.375000	0.000000

The data for the $LiYO_2$:10%Yb^{3+}, 1%Er^{3+} at 190 K:

Global Parameters

Number of used phases:	3	Asymmetry Type:	No Asymmetry Function
Number of variables:	21		
Number of constraints:	2	Asymmetry 1:	0.000000
Zero shift/ °2Theta:	0.000000	Asymmetry 2:	0.000000
Specimen displacement/ mm :	0.183(1)	Shape Type:	Shape Individual
Profile function:	Pseudo Voigt	Shape 1 Left:	0.600000
Background:	Polynomial	Shape 2 Left:	0.000000
R (expected)/ %:	0.59154	Shape 3 Left:	0.000000
R (profile)/ %:	4.11880	Shape 1 Right:	0.600000
R (weighted profile)/ %:	5.85555	Shape 2 Right:	0.000000
GOF:	97.98842	Shape 3 Right:	0.000000
d-statistic:	0.13971	K a1/a2 intensity ratio:	0.500000
U standard:	0.000000	K alpha/beta intensity ratio:	0.000000
V standard:	0.000000	Crystal Shape Factor K:	1.0000
W standard:	0.010000	Instrumental FWHM Curve Type:	Caglioti function
U Left:	0.000000	Instr. Gauss Curve Coefficient A:	0.0045(5)
V Left:	0.000000	Instr. Gauss Curve Coefficient B:	-0.0032(9)
W Left:	0.010000	Instr. Gauss Curve Coefficient C:	0.0046(3)
U Right:	0.000000	Instr. Lorentz Curve Coefficient A:	0.0062(7)
V Right:	0.000000	Instr. Lorentz Curve Coefficient B:	-0.004(1)
W Right:	0.010000	Instr. Lorentz Curve Coefficient C:	0.0064(5)

Relevant parameters of $LiYO_2$-LT, 50992-ICSD

Structure and profile data:		Extinction:	0.000000
Formula sum: $Y_{3.56}Li_{4.00}O_{8.00}Yb_{0.40}Er_{0.04}$		Flat Plate Absorption Correction:	0.000000
Formula mass/ g/mol:	548.1706	Porosity:	0.000000
Density (calculated)/ g/cm^3	4.4442	Roughness:	0.000000
F(000):	245.5600	Fitting mode:	Structure Fit
Weight fraction/ %:	79.2(3)	U Left:	0.06(1)
Space group (No.):	P 1 21/c 1 (14)	V Left:	-0.01(1)
Lattice parameters:		W Left:	0.027(2)
a/ A:	6.1011(2)	Preferred orientation direction/ hkl:	0.00 0.00 1.00
b/ A:	6.1872(2)	Preferred orientation parameter:	1.000000
c/ A:	6.1793(2)	Asymmetry parameter 1:	0.09(2)
alpha/ °:	90	Asymmetry parameter 2:	0.000000
beta/ °:	118.604(2)	Peak shape:	
gamma/ °:	90	parameter 1 Left:	0.47(1)
V/ 10^6 pm^3	204.79090	parameter 2 Left:	0.000000
Overall displacement parameter:	0.000000	parameter 3 Left:	0.000000
		R (Bragg)/ %:	6.04073

Occupancy, atomic fract. coordinates and Biso for $LiYO_2$-LT, 50992-ICSD

Atom	Wyck.	s.o.f.	x	y	z	B/ 10^4 pm^2
Y1	4e	0.890000	0.266000	0.131200	0.020700	0.210000
Li1	4e	1.000000	0.791000	0.346000	0.068000	0.600000
O1	4e	1.000000	0.045900	0.107000	0.237800	0.280000
O2	4e	1.000000	0.512100	0.328800	0.343900	0.290000
Yb1	4e	0.100000	0.266000	0.131200	0.020700	0.210000
Er1	4e	0.010000	0.266000	0.131200	0.020700	0.210000

Relevant parameters of $LiYO_2$-HT, 50993-ICSD

Structure and profile data:		gamma/ °:	90
Formula sum: $Y_{3.56}Li_{4.00}O_{8.00}Yb_{0.40}Er_{0.04}$		V/ 10^6 pm^3	202.19570
Formula mass/ g/mol:	548.1706	Overall displacement parameter:	0.000000
Density (calculated)/ g/cm^3	4.5012	Extinction:	0.000000
F(000):	245.5600	Flat Plate Absorption Correction:	0.000000
Weight fraction/ %:	13.7(2)	Porosity:	0.000000
Space group (No.): I 41/a m d (141)		Roughness:	0.000000
Lattice parameters:		Fitting mode:	Structure Fit
a/ A:	4.4361(2)	U Left:	0.068937
b/ A:	4.4361(2)	V Left:	-0.052995
c/ A:	10.2748(6)	W Left:	0.027085
alpha/ °:	90	Preferred orientation direction/ hkl:	0.00 0.00 1.00
beta/ °:	90	Preferred orientation parameter:	1.000000
		Asymmetry parameter 1:	0.170069

Asymmetry parameter 2:	0.000000	parameter 2 Left:	0.000000
Peak shape:		parameter 3 Left:	0.000000
parameter 1 Left:	0.626711	R (Bragg)/ %:	5.20558

Occupancy, atomic fract. coordinates and Biso for $LiYO_2$-HT, 50993-ICSD

Atom	Wyck.	s.o.f.	x	y	z	B/ 10^4 pm^2
Y1	4b	0.890000	0.000000	0.250000	0.375000	0.000000
Li1	4b	0.000000	0.000000	0.250000	0.375000	0.000000
Li2	4a	1.000000	0.000000	0.750000	0.125000	0.000000
O1	8e	1.000000	0.000000	0.250000	0.152000	0.000000
Yb1	4b	0.100000	0.000000	0.250000	0.375000	0.000000
Er1	4b	0.010000	0.000000	0.250000	0.375000	0.000000

The data for the $LiYO_2$:10%Yb^{3+}, 1%Er^{3+} at 200 K:

Global Parameters

Number of used phases:	3		Function
Number of variables:	21	Asymmetry 1:	0.000000
Number of constraints:	2	Asymmetry 2:	0.000000
Zero shift/ °2Theta:	0.000000	Shape Type:	Shape Individual
Specimen displacement/ mm :	0.181(1)	Shape 1 Left:	0.600000
Profile function:	Pseudo Voigt	Shape 2 Left:	0.000000
Background:	Polynomial	Shape 3 Left:	0.000000
R (expected)/ %:	0.59146	Shape 1 Right:	0.600000
R (profile)/ %:	3.99934	Shape 2 Right:	0.000000
R (weighted profile)/ %:	5.73383	Shape 3 Right:	0.000000
GOF:	93.98151	K a1/a2 intensity ratio:	0.500000
d-statistic:	0.14743	K alpha/beta intensity ratio:	0.000000
U standard:	0.000000	Crystal Shape Factor K:	1.0000
V standard:	0.000000	Instrumental FWHM Curve Type:	Caglioti function
W standard:	0.010000	Instr. Gauss Curve Coefficient A:	0.0045(5)
U Left:	0.000000	Instr. Gauss Curve Coefficient B:	-0.0032(9)
V Left:	0.000000	Instr. Gauss Curve Coefficient C:	0.0046(3)
W Left:	0.010000	Instr. Lorentz Curve Coefficient A:	0.0062(7)
U Right:	0.000000	Instr. Lorentz Curve Coefficient B:	-0.004(1)
V Right:	0.000000	Instr. Lorentz Curve Coefficient C:	0.0064(5)
W Right:	0.010000		
Asymmetry Type:	No Asymmetry		

Relevant parameters of $LiYO_2$-LT, 50992-ICSD

Structure and profile data:		Extinction:	0.000000
Formula sum: $Y_{3.56}Li_{4.00}O_{8.00}Yb_{0.40}Er_{0.04}$		Flat Plate Absorption Correction:	0.000000
Formula mass/ g/mol:	548.1706	Porosity:	0.000000
Density (calculated)/ g/cm^3	4.4445	Roughness:	0.000000
F(000):	245.5600	Fitting mode:	Structure Fit
Weight fraction/ %:	76.4(3)	U Left:	0.05(1)
Space group (No.):	P 1 21/c 1 (14)	V Left:	-0.01(1)
Lattice parameters:		W Left:	0.027(2)
a/ A:	6.1003(2)	Preferred orientation direction/ hkl:	0.00 0.00 1.00
b/ A:	6.1887(2)	Preferred orientation parameter:	1.000000
c/ A:	6.1770(2)	Asymmetry parameter 1:	0.10(2)
alpha/ °:	90	Asymmetry parameter 2:	0.000000
beta/ °:	118.585(2)	Peak shape:	
gamma/ °:	90	parameter 1 Left:	0.44(1)
V/ 10^6 pm^3	204.77470	parameter 2 Left:	0.000000
Overall displacement parameter:	0.000000	parameter 3 Left:	0.000000
		R (Bragg)/ %:	5.88292

Occupancy, atomic fract. coordinates and Biso for $LiYO_2$-LT, 50992-ICSD

Atom	Wyck.	s.o.f.	x	y	z	B/ 10^4 pm^2
Y1	4e	0.890000	0.266000	0.131200	0.020700	0.210000
Li1	4e	1.000000	0.791000	0.346000	0.068000	0.600000
O1	4e	1.000000	0.045900	0.107000	0.237800	0.280000
O2	4e	1.000000	0.512100	0.328800	0.343900	0.290000
Yb1	4e	0.100000	0.266000	0.131200	0.020700	0.210000
Er1	4e	0.010000	0.266000	0.131200	0.020700	0.210000

Relevant parameters of $LiYO_2$-HT, 50993-ICSD

Structure and profile data:		Formula sum: $Y_{3.56}Li_{4.00}O_{8.00}Yb_{0.40}Er_{0.04}$	

Formula mass/ g/mol:	548.1706	Flat Plate Absorption Correction:	0.000000
Density (calculated)/ g/cm^3	4.5003	Porosity:	0.000000
F(000):	245.5600	Roughness:	0.000000
Weight fraction/ %:	16.3(2)	Fitting mode:	Structure Fit
Space group (No.):	I 41/a m d (141)	U Left:	0.068937
		V Left:	-0.052995
Lattice parameters:		W Left:	0.027085
a/ A:	4.4365(2)	Preferred orientation direction/ hkl:	0.00 0.00 1.00
b/ A:	4.4365(2)	Preferred orientation parameter:	1.000000
c/ A:	10.2751(5)	Asymmetry parameter 1:	0.170069
alpha/ °:	90	Asymmetry parameter 2:	0.000000
beta/ °:	90	Peak shape:	
gamma/ °:	90	parameter 1 Left:	0.626711
V/ 10^6 pm^3	202.23650	parameter 2 Left:	0.000000
Overall displacement parameter:	0.000000	parameter 3 Left:	0.000000
Extinction:	0.000000	R (Bragg)/ %:	5.37426

Occupancy, atomic fract. coordinates and Biso for $LiYO_2$-HT, 50993-ICSD

Atom	Wyck.	s.o.f.	x	y	z	B/ 10^4 pm^2
Y1	4b	0.890000	0.000000	0.250000	0.375000	0.000000
Li1	4b	0.000000	0.000000	0.250000	0.375000	0.000000
Li2	4a	1.000000	0.000000	0.750000	0.125000	0.000000
O1	8e	1.000000	0.000000	0.250000	0.152000	0.000000
Yb1	4b	0.100000	0.000000	0.250000	0.375000	0.000000
Er1	4b	0.010000	0.000000	0.250000	0.375000	0.000000

The data for the $LiYO_2$:10%Yb^{3+}, 1%Er^{3+} at 210 K:

Global Parameters

Number of used phases:	3	Asymmetry Type:	No Asymmetry Function
Number of variables:	21		
Number of constraints:	2	Asymmetry 1:	0.000000
Zero shift/ °2Theta:	0.000000	Asymmetry 2:	0.000000
Specimen displacement/ mm :	0.177(1)	Shape Type:	Shape Individual
Profile function:	Pseudo Voigt	Shape 1 Left:	0.600000
Background:	Polynomial	Shape 2 Left:	0.000000
R (expected)/ %:	0.59207	Shape 3 Left:	0.000000
R (profile)/ %:	3.97807	Shape 1 Right:	0.600000
R (weighted profile)/ %:	5.66528	Shape 2 Right:	0.000000
GOF:	91.55856	Shape 3 Right:	0.000000
d-statistic:	0.14841	K a1/a2 intensity ratio:	0.500000
U standard:	0.000000	K alpha/beta intensity ratio:	0.000000
V standard:	0.000000	Crystal Shape Factor K:	1.0000
W standard:	0.010000	Instrumental FWHM Curve Type:	Caglioti function
U Left:	0.000000	Instr. Gauss Curve Coefficient A:	0.0045(5)
V Left:	0.000000	Instr. Gauss Curve Coefficient B:	-0.0032(9)
W Left:	0.010000	Instr. Gauss Curve Coefficient C:	0.0046(3)
U Right:	0.000000	Instr. Lorentz Curve Coefficient A:	0.0062(7)
V Right:	0.000000	Instr. Lorentz Curve Coefficient B:	-0.004(1)
W Right:	0.010000	Instr. Lorentz Curve Coefficient C:	0.0064(5)

Relevant parameters of $LiYO_2$-LT, 50992-ICSD

Structure and profile data:		Extinction:	0.000000
Formula sum: $Y_{3.56}Li_{4.00}O_{8.00}Yb_{0.40}Er_{0.04}$		Flat Plate Absorption Correction:	0.000000
Formula mass/ g/mol:	548.1706	Porosity:	0.000000
		Roughness:	0.000000
Density (calculated)/ g/cm^3	4.4444	Fitting mode:	Structure Fit
F(000):	245.5600	U Left:	0.06(1)
Weight fraction/ %:	72.4(3)	V Left:	-0.03(1)
Space group (No.):	P 1 21/c 1 (14)	W Left:	0.030(2)
Lattice parameters:		Preferred orientation direction/ hkl:	0.00 0.00 1.00
a/ A:	6.0995(2)	Preferred orientation parameter:	1.000000
b/ A:	6.1907(2)	Asymmetry parameter 1:	0.14(2)
c/ A:	6.1750(2)	Asymmetry parameter 2:	0.000000
alpha/ °:	90	Peak shape:	
beta/ °:	118.569(2)	parameter 1 Left:	0.43(1)
gamma/ °:	90	parameter 2 Left:	0.000000
V/ 10^6 pm^3	204.77910	parameter 3 Left:	0.000000
Overall displacement parameter:	0.000000	R (Bragg)/ %:	5.72081

Occupancy, atomic fract. coordinates and Biso for $LiYO_2$-LT, 50992-ICSD

Atom	Wyck.	s.o.f.	x	y	z	B/ 10^4 pm^2
Y1	4e	0.890000	0.266000	0.131200	0.020700	0.210000
Li1	4e	1.000000	0.791000	0.346000	0.068000	0.600000
O1	4e	1.000000	0.045900	0.107000	0.237800	0.280000
O2	4e	1.000000	0.512100	0.328800	0.343900	0.290000
Yb1	4e	0.100000	0.266000	0.131200	0.020700	0.210000
Er1	4e	0.010000	0.266000	0.131200	0.020700	0.210000

Relevant parameters of $LiYO_2$-HT, 50993-ICSD

Structure and profile data:
Formula sum: $Y_{3.56}Li_{4.00}O_{8.00}Yb_{0.40}Er_{0.04}$

Formula mass/ g/mol:	548.1706
Density (calculated)/ g/cm^3	4.4972
F(000):	245.5600
Weight fraction/ %:	20.4(2)
Space group (No.): I 41/a m d (141)	
Lattice parameters:	
a/ A:	4.4369(1)
b/ A:	4.4369(1)
c/ A:	10.2803(4)
alpha/ °:	90
beta/ °:	90
gamma/ °:	90
V/ 10^6 pm^3	202.37900
Overall displacement parameter:	0.000000
Extinction:	0.000000
Flat Plate Absorption Correction:	0.000000
Porosity:	0.000000
Roughness:	0.000000
Fitting mode:	Structure Fit
U Left:	0.068937
V Left:	-0.052995
W Left:	0.027085
Preferred orientation direction/ hkl:	0.00 0.00 1.00
Preferred orientation parameter:	1.000000
Asymmetry parameter 1:	0.170069
Asymmetry parameter 2:	0.000000
Peak shape:	
parameter 1 Left:	0.626711
parameter 2 Left:	0.000000
parameter 3 Left:	0.000000
R (Bragg)/ %:	5.59368

Occupancy, atomic fract. coordinates and Biso for $LiYO_2$-HT, 50993-ICSD

Atom	Wyck.	s.o.f.	x	y	z	B/ 10^4 pm^2
Y1	4b	0.890000	0.000000	0.250000	0.375000	0.000000
Li1	4b	0.000000	0.000000	0.250000	0.375000	0.000000
Li2	4a	1.000000	0.000000	0.750000	0.125000	0.000000
O1	8e	1.000000	0.000000	0.250000	0.152000	0.000000
Yb1	4b	0.100000	0.000000	0.250000	0.375000	0.000000
Er1	4b	0.010000	0.000000	0.250000	0.375000	0.000000

The data for the $LiYO_2$:10%Yb^{3+}, 1%Er^{3+} at 220 K:

Global Parameters

Number of used phases:	3
Number of variables:	23
Number of constraints:	2
Zero shift/ °2Theta:	0.000000
Specimen displacement/ mm :	0.170(1)
Profile function:	Pseudo Voigt
Background:	Polynomial
R (expected)/ %:	0.59317
R (profile)/ %:	3.90880
R (weighted profile)/ %:	5.61894
GOF:	89.73110
d-statistic:	0.14992
U standard:	0.000000
V standard:	0.000000
W standard:	0.010000
U Left:	0.000000
V Left:	0.000000
W Left:	0.010000
U Right:	0.000000
V Right:	0.000000
W Right:	0.010000
Asymmetry Type:	No Asymmetry Function
Asymmetry 1:	0.000000
Asymmetry 2:	0.000000
Shape Type:	Shape Individual
Shape 1 Left:	0.600000
Shape 2 Left:	0.000000
Shape 3 Left:	0.000000
Shape 1 Right:	0.600000
Shape 2 Right:	0.000000
Shape 3 Right:	0.000000
K a1/a2 intensity ratio:	0.500000
K alpha/beta intensity ratio:	0.000000
Crystal Shape Factor K:	1.0000
Instrumental FWHM Curve Type:	Caglioti function
Instr. Gauss Curve Coefficient A:	0.0045(5)
Instr. Gauss Curve Coefficient B:	-0.0032(9)
Instr. Gauss Curve Coefficient C:	0.0046(3)
Instr. Lorentz Curve Coefficient A:	0.0062(7)
Instr. Lorentz Curve Coefficient B:	-0.004(1)
Instr. Lorentz Curve Coefficient C:	0.0064(5)

Relevant parameters of $LiYO_2$-LT, 50992-ICSD

Structure and profile data:
Formula sum: $Y_{3.56}Li_{4.00}O_{8.00}Yb_{0.40}Er_{0.04}$

Formula mass/ g/mol:	548.1706
Density (calculated)/ g/cm^3	4.4444
F(000):	245.5600
Weight fraction/ %:	66.4(4)
Space group (No.):	P 1 21/c 1 (14)
Lattice parameters:	
a/ A:	6.0991(2)
b/ A:	6.1923(2)
c/ A:	6.1731(2)
alpha/ °:	90
beta/ °:	118.555(2)
gamma/ °:	90

V/ 10^6 pm^3	204.78150	W Left:	0.027(2)
Overall displacement parameter:	0.000000	Preferred orientation direction/ hkl:	0.00 0.00 1.00
Extinction:	0.000000	Preferred orientation parameter:	1.000000
Flat Plate Absorption Correction:	0.000000	Asymmetry parameter 1:	0.09(2)
Porosity:	0.000000	Asymmetry parameter 2:	0.000000
Roughness:	0.000000	Peak shape:	
Fitting mode:	Structure Fit	parameter 1 Left:	0.43(2)
U Left:	0.04(1)	parameter 2 Left:	0.000000
V Left:	-0.012(9)	parameter 3 Left:	0.000000
		R (Bragg)/ %:	5.47725

Occupancy, atomic fract. coordinates and Biso for $LiYO_2$-LT, 50992-ICSD

Atom	Wyck.	s.o.f.	x	y	z	B/ 10^4 pm^2
Y1	4e	0.890000	0.266000	0.131200	0.020700	0.210000
Li1	4e	1.000000	0.791000	0.346000	0.068000	0.600000
O1	4e	1.000000	0.045900	0.107000	0.237800	0.280000
O2	4e	1.000000	0.512100	0.328800	0.343900	0.290000
Yb1	4e	0.100000	0.266000	0.131200	0.020700	0.210000
Er1	4e	0.010000	0.266000	0.131200	0.020700	0.210000

Relevant parameters of $LiYO_2$-HT, 50993-ICSD

Structure and profile data:		Extinction:	0.000000
Formula sum: $Y_{3.56}Li_{4.00}O_{8.00}Yb_{0.40}Er_{0.04}$		Flat Plate Absorption Correction:	0.000000
Formula mass/ g/mol:	548.1706	Porosity:	0.000000
Density (calculated)/ g/cm^3	4.4944	Roughness:	0.000000
F(000):	245.5600	Fitting mode:	Structure Fit
Weight fraction/ %:	26.0(3)	U Left:	0.068937
Space group (No.): I 41/a m d (141)		V Left:	-0.052995
Lattice parameters:		W Left:	0.0306(7)
a/ A:	4.4371(1)	Preferred orientation direction/ hkl:	0.00 0.00 1.00
b/ A:	4.4371(1)	Preferred orientation parameter:	1.000000
c/ A:	10.2855(4)	Asymmetry parameter 1:	0.170069
alpha/ °:	90	Asymmetry parameter 2:	0.000000
beta/ °:	90	Peak shape:	
gamma/ °:	90	parameter 1 Left:	0.60(4)
V/ 10^6 pm^3	202.50110	parameter 2 Left:	0.000000
Overall displacement parameter:	0.000000	parameter 3 Left:	0.000000
		R (Bragg)/ %:	5.91716

Occupancy, atomic fract. coordinates and Biso for $LiYO_2$-HT, 50993-ICSD

Atom	Wyck.	s.o.f.	x	y	z	B/ 10^4 pm^2
Y1	4b	0.890000	0.000000	0.250000	0.375000	0.000000
Li1	4b	0.000000	0.000000	0.250000	0.375000	0.000000
Li2	4a	1.000000	0.000000	0.750000	0.125000	0.000000
O1	8e	1.000000	0.000000	0.250000	0.152000	0.000000
Yb1	4b	0.100000	0.000000	0.250000	0.375000	0.000000
Er1	4b	0.010000	0.000000	0.250000	0.375000	0.000000

The data for the $LiYO_2$:10%Yb^{3+}, 1%Er^{3+} at 240 K:

Global Parameters

Number of used phases:	3	Asymmetry Type:	No Asymmetry Function
Number of variables:	24		
Number of constraints:	2	Asymmetry 1:	0.000000
Zero shift/ °2Theta:	0.000000	Asymmetry 2:	0.000000
Specimen displacement/ mm :	0.153(1)	Shape Type:	Shape Individual
Profile function:	Pseudo Voigt	Shape 1 Left:	0.600000
Background:	Polynomial	Shape 2 Left:	0.000000
R (expected)/ %:	0.59078	Shape 3 Left:	0.000000
R (profile)/ %:	3.87008	Shape 1 Right:	0.600000
R (weighted profile)/ %:	5.63503	Shape 2 Right:	0.000000
GOF:	90.97943	Shape 3 Right:	0.000000
d-statistic:	0.13566	K a1/a2 intensity ratio:	0.500000
U standard:	0.000000	K alpha/beta intensity ratio:	0.000000
V standard:	0.000000	Crystal Shape Factor K:	1.0000
W standard:	0.010000	Instrumental FWHM Curve Type:	Caglioti function
U Left:	0.000000	Instr. Gauss Curve Coefficient A:	0.0045(5)
V Left:	0.000000	Instr. Gauss Curve Coefficient B:	-0.0032(9)
W Left:	0.010000	Instr. Gauss Curve Coefficient C:	0.0046(3)
U Right:	0.000000	Instr. Lorentz Curve Coefficient A:	0.0062(7)
V Right:	0.000000	Instr. Lorentz Curve Coefficient B:	-0.004(1)
W Right:	0.010000	Instr. Lorentz Curve Coefficient C:	0.0064(5)

Relevant parameters of $LiYO_2$-LT, 50992-ICSD

Structure and profile data:		Extinction:	0.000000
Formula sum: $Y_{3.56}Li_{4.00}O_{8.00}Yb_{0.40}Er_{0.04}$		Flat Plate Absorption Correction:	0.000000
Formula mass/ g/mol:	548.1706	Porosity:	0.000000
Density (calculated)/ g/cm^3	4.4434	Roughness:	0.000000
F(000):	245.5600	Fitting mode:	Structure Fit
Weight fraction/ %:	43.8(4)	U Left:	0.06(1)
Space group (No.):	P 1 21/c 1 (14)	V Left:	-0.05(1)
Lattice parameters:		W Left:	0.032(2)
a/ A:	6.0988(2)	Preferred orientation direction/ hkl:	0.00 0.00 1.00
b/ A:	6.1952(2)	Preferred orientation parameter:	1.000000
c/ A:	6.1703(2)	Asymmetry parameter 1:	0.026145
alpha/ °:	90	Asymmetry parameter 2:	0.000000
beta/ °:	118.530(3)	Peak shape:	
gamma/ °:	90	parameter 1 Left:	0.37(3)
V/ 10^6 pm^3	204.82560	parameter 2 Left:	0.000000
Overall displacement parameter:	0.000000	parameter 3 Left:	0.000000
		R (Bragg)/ %:	5.64792

Occupancy, atomic fract. coordinates and Biso for $LiYO_2$-LT, 50992-ICSD

Atom	Wyck.	s.o.f.	x	y	z	B/ 10^4 pm^2
Y1	4e	0.890000	0.266000	0.131200	0.020700	0.210000
Li1	4e	1.000000	0.791000	0.346000	0.068000	0.600000
O1	4e	1.000000	0.045900	0.107000	0.237800	0.280000
O2	4e	1.000000	0.512100	0.328800	0.343900	0.290000
Yb1	4e	0.100000	0.266000	0.131200	0.020700	0.210000
Er1	4e	0.010000	0.266000	0.131200	0.020700	0.210000

Relevant parameters of $LiYO_2$-HT, 50993-ICSD

Structure and profile data:		Extinction:	0.000000
Formula sum: $Y_{3.56}Li_{4.00}O_{8.00}Yb_{0.40}Er_{0.04}$		Flat Plate Absorption Correction:	0.000000
Formula mass/ g/mol:	548.1706	Porosity:	0.000000
Density (calculated)/ g/cm^3	4.4890	Roughness:	0.000000
F(000):	245.5600	Fitting mode:	Structure Fit
Weight fraction/ %:	48.6(3)	U Left:	0.067(8)
Space group (No.): I 41/a m d (141)		V Left:	-0.054(7)
Lattice parameters:		W Left:	0.029(1)
a/ A:	4.43668(9)	Preferred orientation direction/ hkl:	0.00 0.00 1.00
b/ A:	4.43668(9)	Preferred orientation parameter:	1.000000
c/ A:	10.3000(2)	Asymmetry parameter 1:	0.170069
alpha/ °:	90	Asymmetry parameter 2:	0.000000
beta/ °:	90	Peak shape:	
gamma/ °:	90	parameter 1 Left:	0.66(2)
V/ 10^6 pm^3	202.74720	parameter 2 Left:	0.000000
Overall displacement parameter:	0.000000	parameter 3 Left:	0.000000
		R (Bragg)/ %:	6.25824

Occupancy, atomic fract. coordinates and Biso for $LiYO_2$-HT, 50993-ICSD

Atom	Wyck.	s.o.f.	x	y	z	B/ 10^4 pm^2
Y1	4b	0.890000	0.000000	0.250000	0.375000	0.000000
Li1	4b	0.000000	0.000000	0.250000	0.375000	0.000000
Li2	4a	1.000000	0.000000	0.750000	0.125000	0.000000
O1	8e	1.000000	0.000000	0.250000	0.152000	0.000000
Yb1	4b	0.100000	0.000000	0.250000	0.375000	0.000000
Er1	4b	0.010000	0.000000	0.250000	0.375000	0.000000

The data for the $LiYO_2$:10%Yb^{3+}, 1%Er^{3+} at 260 K:

Global Parameters

Number of used phases:	3	GOF:	98.59666
Number of variables:	17	d-statistic:	0.11953
Number of constraints:	2	U standard:	0.000000
Zero shift/ °2Theta:	0.000000	V standard:	0.000000
Specimen displacement/ mm :	0.1398(8)	W standard:	0.010000
Profile function:	Pseudo Voigt	U Left:	0.000000
Background:	Polynomial	V Left:	0.000000
R (expected)/ %:	0.59230	W Left:	0.010000
R (profile)/ %:	3.89771	U Right:	0.000000
R (weighted profile)/ %:	5.88128	V Right:	0.000000

W Right:	0.010000	Shape 3 Right:	0.000000
Asymmetry Type:	No Asymmetry Function	K a1/a2 intensity ratio:	0.500000
		K alpha/beta intensity ratio:	0.000000
Asymmetry 1:	0.000000	Crystal Shape Factor K:	1.0000
Asymmetry 2:	0.000000	Instrumental FWHM Curve Type:	Caglioti function
Shape Type:	Shape Individual	Instr. Gauss Curve Coefficient A:	0.0045(5)
Shape 1 Left:	0.600000	Instr. Gauss Curve Coefficient B:	-0.0032(9)
Shape 2 Left:	0.000000	Instr. Gauss Curve Coefficient C:	0.0046(3)
Shape 3 Left:	0.000000	Instr. Lorentz Curve Coefficient A:	0.0062(7)
Shape 1 Right:	0.600000	Instr. Lorentz Curve Coefficient B:	-0.004(1)
Shape 2 Right:	0.000000	Instr. Lorentz Curve Coefficient C:	0.0064(5)

Relevant parameters of $LiYO_2$-LT, 50992-ICSD

Structure and profile data:		Extinction:	0.000000
Formula sum: $Y_{3.56}Li_{4.00}O_{8.00}Yb_{0.40}Er_{0.04}$		Flat Plate Absorption Correction:	0.000000
Formula mass/ g/mol:	548.1706	Porosity:	0.000000
Density (calculated)/ g/cm^3	4.4433	Roughness:	0.000000
F(000):	245.5600	Fitting mode:	Structure Fit
Weight fraction/ %:	3.0(2)	U Left:	0.063012
Space group (No.):	P 1 21/c 1 (14)	V Left:	-0.051065
Lattice parameters:		W Left:	0.031423
a/ A:	6.098925	Preferred orientation direction/ hkl:	0.00 0.00 1.00
b/ A:	6.195206	Preferred orientation parameter:	1.000000
c/ A:	6.170460	Asymmetry parameter 1:	0.026145
alpha/ °:	90	Asymmetry parameter 2:	0.000000
beta/ °:	118.531900	Peak shape:	
gamma/ °:	90	parameter 1 Left:	0.386637
V/ 10^6 pm^3	204.83000	parameter 2 Left:	0.000000
Overall displacement parameter:	0.000000	parameter 3 Left:	0.000000
		R (Bragg)/ %:	5.79018

Occupancy, atomic fract. coordinates and Biso for $LiYO_2$-LT, 50992-ICSD

Atom	Wyck.	s.o.f.	x	y	z	B/ 10^4 pm^2
Y1	4e	0.890000	0.266000	0.131200	0.020700	0.210000
Li1	4e	1.000000	0.791000	0.346000	0.068000	0.600000
O1	4e	1.000000	0.045900	0.107000	0.237800	0.280000
O2	4e	1.000000	0.512100	0.328800	0.343900	0.290000
Yb1	4e	0.100000	0.266000	0.131200	0.020700	0.210000
Er1	4e	0.010000	0.266000	0.131200	0.020700	0.210000

Relevant parameters of $LiYO_2$-HT, 50993-ICSD

Structure and profile data:		Flat Plate Absorption Correction:	0.000000
Formula sum: $Y_{3.56}Li_{4.00}O_{8.00}Yb_{0.40}Er_{0.04}$		Porosity:	0.000000
Formula mass/ g/mol:	548.1706	Roughness:	0.000000
Density (calculated)/ g/cm^3	4.4859	Fitting mode:	Structure Fit
F(000):	245.5600	U Left:	0.067(4)
Weight fraction/ %:	88.9(3)	V Left:	-0.052(4)
Space group (No.): I 41/a m d (141)		W Left:	0.0268(7)
Lattice parameters:		Preferred orientation direction/ hkl:	0.00 0.00 1.00
a/ A:	4.43613(6)	Preferred orientation parameter:	1.000000
b/ A:	4.43613(6)	Asymmetry parameter 1:	0.18(2)
c/ A:	10.3098(2)	Asymmetry parameter 2:	0.000000
alpha/ °:	90	Peak shape:	
beta/ °:	90	parameter 1 Left:	0.599(9)
gamma/ °:	90	parameter 2 Left:	0.000000
V/ 10^6 pm^3	202.88820	parameter 3 Left:	0.000000
Overall displacement parameter:	0.000000	R (Bragg)/ %:	6.27540
Extinction:	0.000000		

Occupancy, atomic fract. coordinates and Biso for $LiYO_2$-HT, 50993-ICSD

Atom	Wyck.	s.o.f.	x	y	z	B/ 10^4 pm^2
Y1	4b	0.890000	0.000000	0.250000	0.375000	0.000000
Li1	4b	0.000000	0.000000	0.250000	0.375000	0.000000
Li2	4a	1.000000	0.000000	0.750000	0.125000	0.000000
O1	8e	1.000000	0.000000	0.250000	0.152000	0.000000
Yb1	4b	0.100000	0.000000	0.250000	0.375000	0.000000
Er1	4b	0.010000	0.000000	0.250000	0.375000	0.000000

The data for the $LiYO_2$:10%Yb^{3+}, 1%Er^{3+} at 280 K:

Global Parameters

Number of used phases:	3
Number of variables:	16
Number of constraints:	2
Zero shift/ °2Theta:	0.000000
Specimen displacement/ mm :	0.1318(8)
Profile function:	Pseudo Voigt
Background:	Polynomial
R (expected)/ %:	0.59157
R (profile)/ %:	4.00301
R (weighted profile)/ %:	6.32535
GOF:	114.32950
d-statistic:	0.10007
U standard:	0.000000
V standard:	0.000000
W standard:	0.010000
U Left:	0.000000
V Left:	0.000000
W Left:	0.010000
U Right:	0.000000
V Right:	0.000000
W Right:	0.010000

Asymmetry Type:	No Asymmetry Function
Asymmetry 1:	0.000000
Asymmetry 2:	0.000000
Shape Type:	Shape Individual
Shape 1 Left:	0.600000
Shape 2 Left:	0.000000
Shape 3 Left:	0.000000
Shape 1 Right:	0.600000
Shape 2 Right:	0.000000
Shape 3 Right:	0.000000
K a1/a2 intensity ratio:	0.500000
K alpha/beta intensity ratio:	0.000000
Crystal Shape Factor K:	1.0000
Instrumental FWHM Curve Type:	Caglioti function
Instr. Gauss Curve Coefficient A:	0.0045(5)
Instr. Gauss Curve Coefficient B:	-0.0032(9)
Instr. Gauss Curve Coefficient C:	0.0046(3)
Instr. Lorentz Curve Coefficient A:	0.0062(7)
Instr. Lorentz Curve Coefficient B:	-0.004(1)
Instr. Lorentz Curve Coefficient C:	0.0064(5)

Relevant parameters of $LiYO_2$-LT, 50992-ICSD

Structure and profile data:	
Formula sum:	$Y_{3.56}Li_{4.00}O_{8.00}$
Formula mass/ g/mol:	472.2642
Density (calculated)/ g/cm^3	3.7970
F(000):	214.8400
Weight fraction/ %:	0.000000
Space group (No.):	P 1 21/c 1 (14)
Lattice parameters:	
a/ A:	6.149002
b/ A:	6.150001
c/ A:	6.249001
alpha/ °:	90
beta/ °:	119.091000
gamma/ °:	90
V/ 10^6 pm^3	206.50310
Overall displacement parameter:	0.000000

Extinction:	0.000000
Flat Plate Absorption Correction:	0.000000
Porosity:	0.000000
Roughness:	0.000000
Fitting mode:	Structure Fit
U Left:	0.000000
V Left:	0.000000
W Left:	0.010000
Preferred orientation direction/ hkl:	0.00 0.00 1.00
Preferred orientation parameter:	1.000000
Asymmetry parameter 1:	0.000000
Asymmetry parameter 2:	0.000000
Peak shape:	
parameter 1 Left:	0.600000
parameter 2 Left:	0.000000
parameter 3 Left:	0.000000
R (Bragg)/ %:	999.00000

Occupancy, atomic fract. coordinates and Biso for $LiYO_2$-LT, 50992-ICSD

Atom	Wyck.	s.o.f.	x	y	z	B/ 10^4 pm^2
Y1	4e	0.890000	0.266000	0.131200	0.020700	0.210000
Li1	4e	1.000000	0.791000	0.346000	0.068000	0.600000
O1	4e	1.000000	0.045900	0.107000	0.237800	0.280000
O2	4e	1.000000	0.512100	0.328800	0.343900	0.290000

Relevant parameters of $LiYO_2$-HT, 50993-ICSD

Structure and profile data:	
Formula sum: $Y_{3.56}Li_{4.00}O_{8.00}Yb_{0.40}Er_{0.04}$	
Formula mass/ g/mol:	548.1706
Density (calculated)/ g/cm^3	4.4843
F(000):	245.5600
Weight fraction/ %:	91.9(3)
Space group (No.): I 41/a m d (141)	
Lattice parameters:	
a/ A:	4.43682(6)
b/ A:	4.43682(6)
c/ A:	10.3101(2)
alpha/ °:	90
beta/ °:	90
gamma/ °:	90
V/ 10^6 pm^3	202.95810
Overall displacement parameter:	0.000000

Extinction:	0.000000
Flat Plate Absorption Correction:	0.000000
Porosity:	0.000000
Roughness:	0.000000
Fitting mode:	Structure Fit
U Left:	0.069(4)
V Left:	-0.051(4)
W Left:	0.0253(7)
Preferred orientation direction/ hkl:	0.00 0.00 1.00
Preferred orientation parameter:	1.000000
Asymmetry parameter 1:	0.25(2)
Asymmetry parameter 2:	0.000000
Peak shape:	
parameter 1 Left:	0.557(9)
parameter 2 Left:	0.000000
parameter 3 Left:	0.000000
R (Bragg)/ %:	7.09510

Occupancy, atomic fract. coordinates and Biso for $LiYO_2$-HT, 50993-ICSD

Atom	Wyck.	s.o.f.	x	y	z	B/ 10^4 pm^2

Y1	4b	0.890000	0.000000	0.250000	0.375000	0.000000
Li1	4b	0.000000	0.000000	0.250000	0.375000	0.000000
Li2	4a	1.000000	0.000000	0.750000	0.125000	0.000000
O1	8e	1.000000	0.000000	0.250000	0.152000	0.000000
Yb1	4b	0.100000	0.000000	0.250000	0.375000	0.000000
Er1	4b	0.010000	0.000000	0.250000	0.375000	0.000000

The data for the $LiYO_2$:10%Yb^{3+}, 1%Er^{3+} at 298 K:

Global Parameters

Number of used phases:	3	Asymmetry Type:	No Asymmetry Function
Number of variables:	16		
Number of constraints:	2	Asymmetry 1:	0.000000
Zero shift/ °2Theta:	0.000000	Asymmetry 2:	0.000000
Specimen displacement/ mm :	0.1235(8)	Shape Type:	Shape Individual
Profile function:	Pseudo Voigt	Shape 1 Left:	0.600000
Background:	Polynomial	Shape 2 Left:	0.000000
R (expected)/ %:	0.58979	Shape 3 Left:	0.000000
R (profile)/ %:	4.07910	Shape 1 Right:	0.600000
R (weighted profile)/ %:	6.39249	Shape 2 Right:	0.000000
GOF:	117.47550	Shape 3 Right:	0.000000
d-statistic:	0.09355	K a1/a2 intensity ratio:	0.500000
U standard:	0.000000	K alpha/beta intensity ratio:	0.000000
V standard:	0.000000	Crystal Shape Factor K:	1.0000
W standard:	0.010000	Instrumental FWHM Curve Type:	Caglioti function
U Left:	0.000000	Instr. Gauss Curve Coefficient A:	0.0045(5)
V Left:	0.000000	Instr. Gauss Curve Coefficient B:	-0.0032(9)
W Left:	0.010000	Instr. Gauss Curve Coefficient C:	0.0046(3)
U Right:	0.000000	Instr. Lorentz Curve Coefficient A:	0.0062(7)
V Right:	0.000000	Instr. Lorentz Curve Coefficient B:	-0.004(1)
W Right:	0.010000	Instr. Lorentz Curve Coefficient C:	0.0064(5)

Relevant parameters of $LiYO_2$-LT, 50992-ICSD

Structure and profile data:		Extinction:	0.000000
Formula sum:	$Y_{3.56}Li_{4.00}O_{8.00}$	Flat Plate Absorption Correction:	0.000000
Formula mass/ g/mol:	472.2642	Porosity:	0.000000
		Roughness:	0.000000
Density (calculated)/ g/cm^3	3.7970	Fitting mode:	Structure Fit
F(000):	214.8400	U Left:	0.000000
Weight fraction/ %:	0.000000	V Left:	0.000000
Space group (No.):	P 1 21/c 1 (14)	W Left:	0.010000
Lattice parameters:		Preferred orientation direction/ hkl:	0.00 0.00 1.00
a/ A:	6.149002	Preferred orientation parameter:	1.000000
b/ A:	6.150001	Asymmetry parameter 1:	0.000000
c/ A:	6.249001	Asymmetry parameter 2:	0.000000
alpha/ °:	90	Peak shape:	
beta/ °:	119.091000	parameter 1 Left:	0.600000
gamma/ °:	90	parameter 2 Left:	0.000000
V/ 10^6 pm^3	206.50310	parameter 3 Left:	0.000000
Overall displacement parameter:	0.000000	R (Bragg)/ %:	999.00000

Occupancy, atomic fract. coordinates and Biso for $LiYO_2$-LT, 50992-ICSD

Atom	Wyck.	s.o.f.	x	y	z	B/ 10^4 pm^2
Y1	4e	0.890000	0.266000	0.131200	0.020700	0.210000
Li1	4e	1.000000	0.791000	0.346000	0.068000	0.600000
O1	4e	1.000000	0.045900	0.107000	0.237800	0.280000
O2	4e	1.000000	0.512100	0.328800	0.343900	0.290000

Relevant parameters of $LiYO_2$-HT, 50993-ICSD

Structure and profile data:
Formula sum: $Y_{3.56}Li_{4.00}O_{8.00}Yb_{0.40}Er_{0.04}$

Formula mass/ g/mol:	548.1706
Density (calculated)/ g/cm^3	4.4821
F(000):	245.5600
Weight fraction/ %:	91.6(3)
Space group (No.): I 41/a m d (141)	
Lattice parameters:	
a/ A:	4.43761(6)
b/ A:	4.43761(6)
c/ A:	10.3116(2)
alpha/ °:	90
beta/ °:	90
gamma/ °:	90
V/ 10^6 pm^3	203.05950
Overall displacement parameter:	0.000000
Extinction:	0.000000
Flat Plate Absorption Correction:	0.000000
Porosity:	0.000000
Roughness:	0.000000
Fitting mode:	Structure Fit
U Left:	0.078(4)
V Left:	-0.064(4)
W Left:	0.0283(7)
Preferred orientation direction/ hkl:	0.00 0.00 1.00
Preferred orientation parameter:	1.000000
Asymmetry parameter 1:	0.26(2)
Asymmetry parameter 2:	0.000000
Peak shape:	
parameter 1 Left:	0.548(9)
parameter 2 Left:	0.000000
parameter 3 Left:	0.000000
R (Bragg)/ %:	7.37902

Occupancy, atomic fract. coordinates and Biso for $LiYO_2$-HT, 50993-ICSD

Atom	Wyck.	s.o.f.	x	y	z	B/ 10^4 pm^2
Y1	4b	0.890000	0.000000	0.250000	0.375000	0.000000
Li1	4b	0.000000	0.000000	0.250000	0.375000	0.000000
Li2	4a	1.000000	0.000000	0.750000	0.125000	0.000000
O1	8e	1.000000	0.000000	0.250000	0.152000	0.000000
Yb1	4b	0.100000	0.000000	0.250000	0.375000	0.000000
Er1	4b	0.010000	0.000000	0.250000	0.375000	0.000000

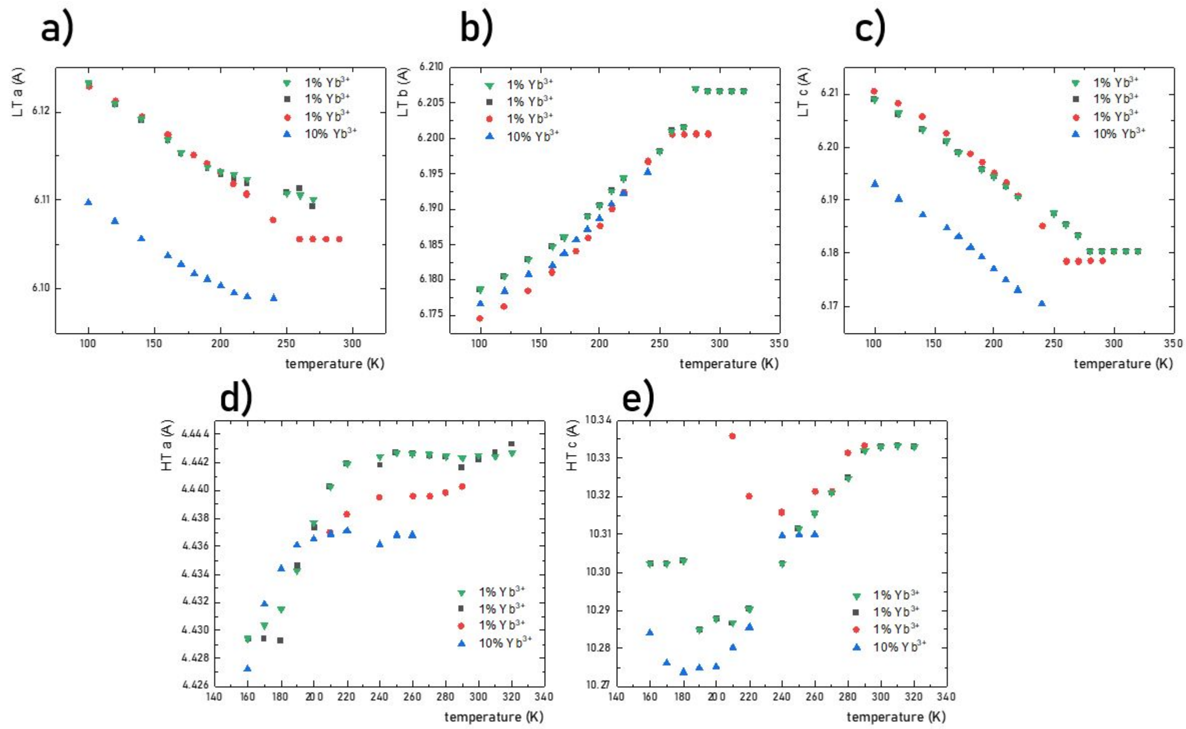

Figure S2. The influence of the temperature on the cell parameters of the low temperature (*a*-a); *b*-b); *c*-c)) (LT) and high temperature (*a*-d); *c*-e) (HT) of $LiYO_2:Er^{3+},Yb^{3+}$ with different concentrations of Yb^{3+} ions determined from the Rietveld refinement based on the XRD patterns

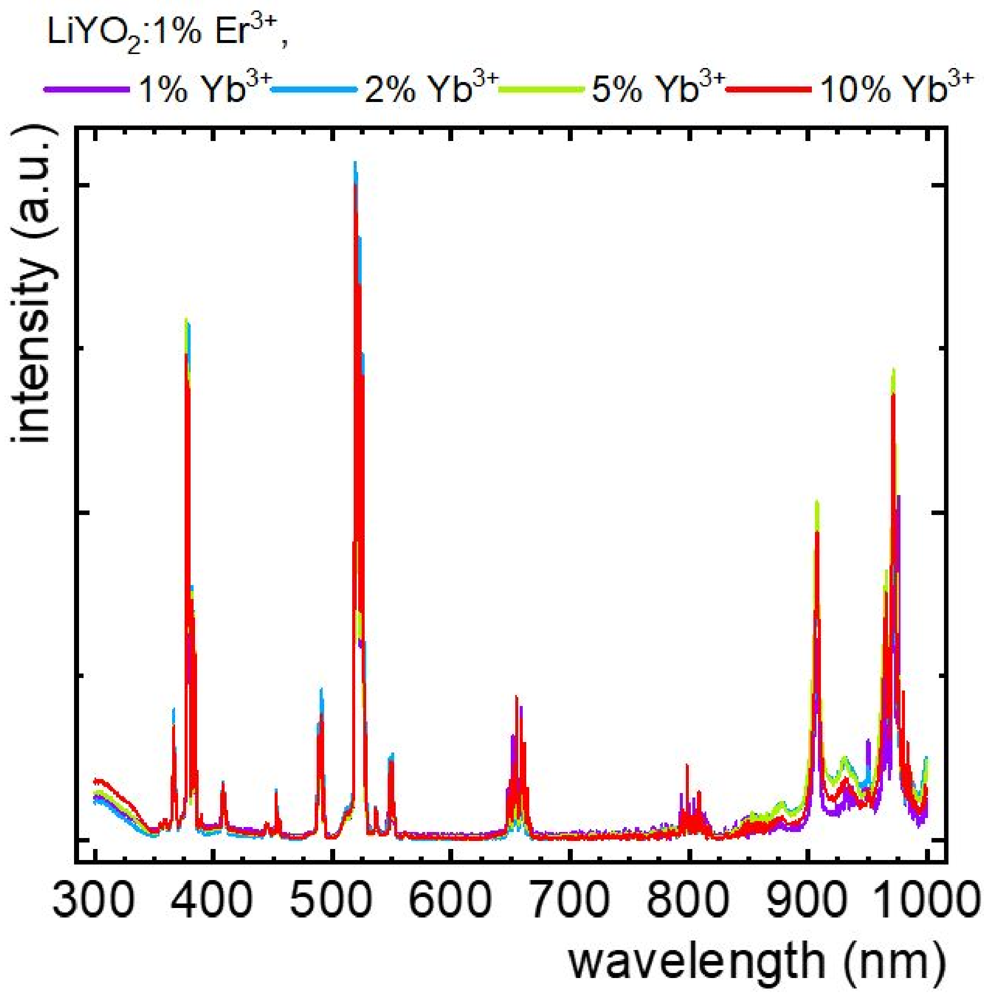

Figure S3. Comparison of excitation spectra of $LiYO_2:Er^{3+},Yb^{3+}$ measured at 83K for λ_{em}=1550 nm

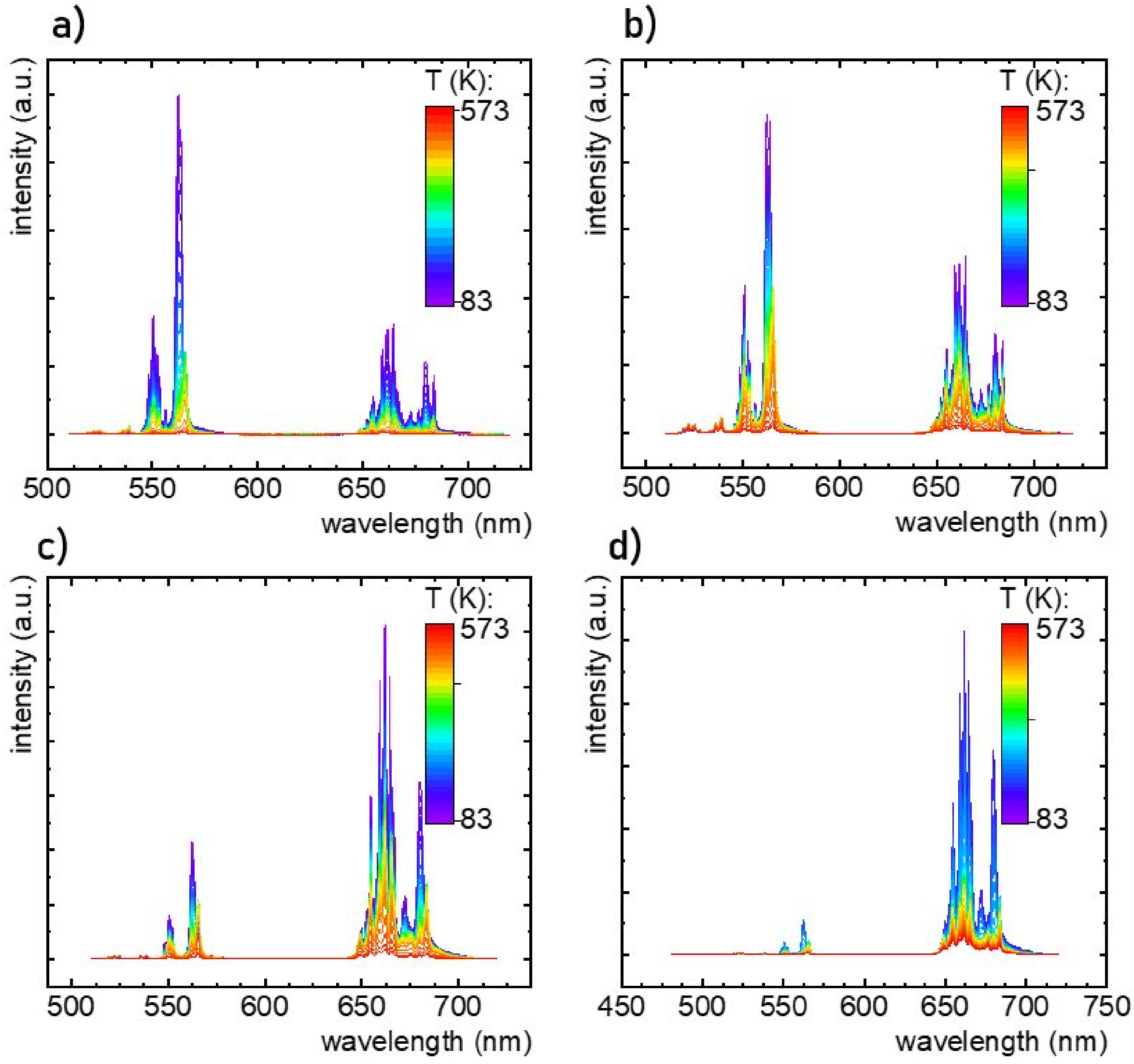

Figure S4. Emission spectra of $LiYO_2:Er^{3+},Yb^{3+}$ measured as a function of temperature for 1%Yb^{3+}-a), 2%Yb^{3+}-a), 5%Yb^{3+}-a) and 10%Yb^{3+}-a)

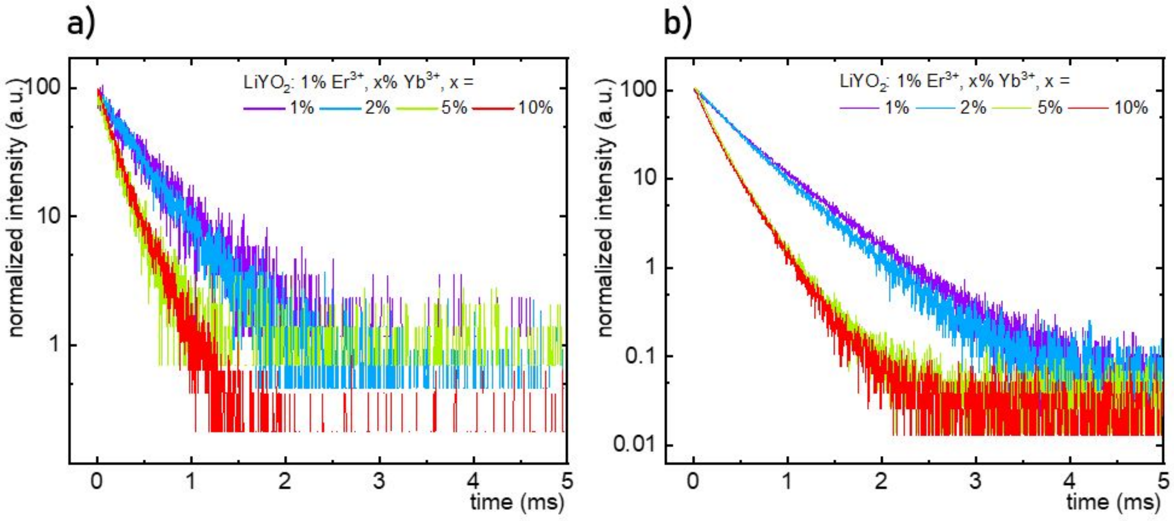

Figure S5. The comparison of the luminescence decay profiles of $LiYO_2$:Er^{3+},Yb^{3+} measured at λ_{em}=520 nm -a) and λ_{em}=570 nm-b).

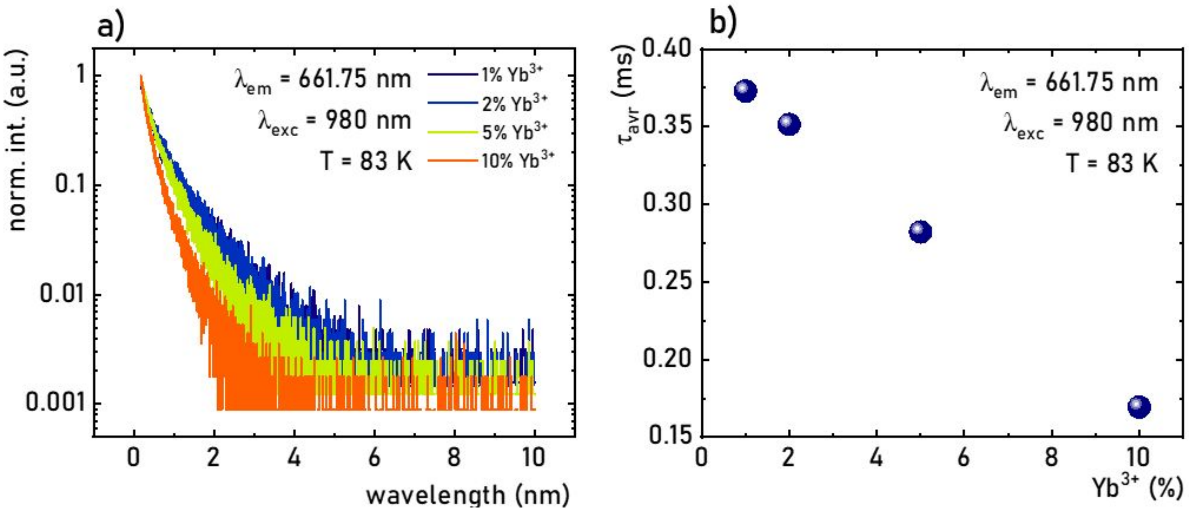

Figure S6. The luminescence decay profiles of $LiYO_2$:Er^{3+},Yb^{3+} measured at λ_{em}=670 nm -a) and τ_{avr} as a function of Yb^{3+} concentration. -b).

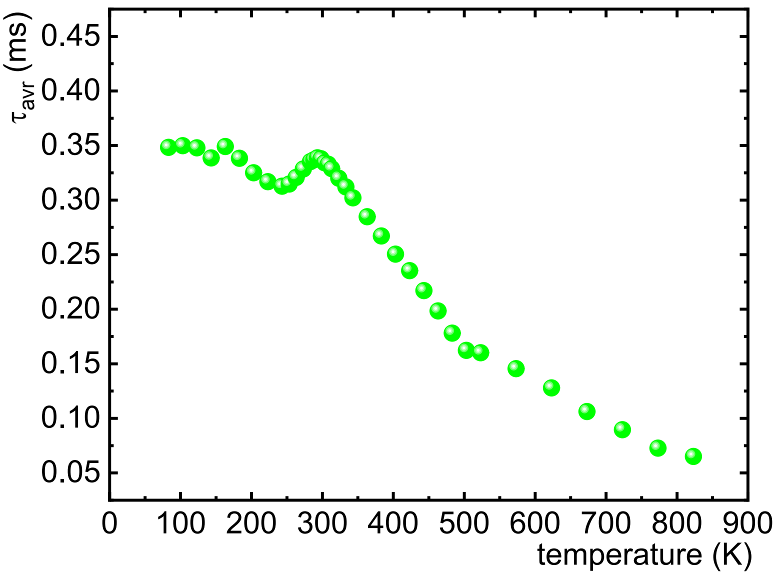

Figure S7. The τ_{avr} of $LiYO_2$:1%Er^{3+},1%Yb^{3+} measured at λ_{em}=570 nm measured as a function of temperature.

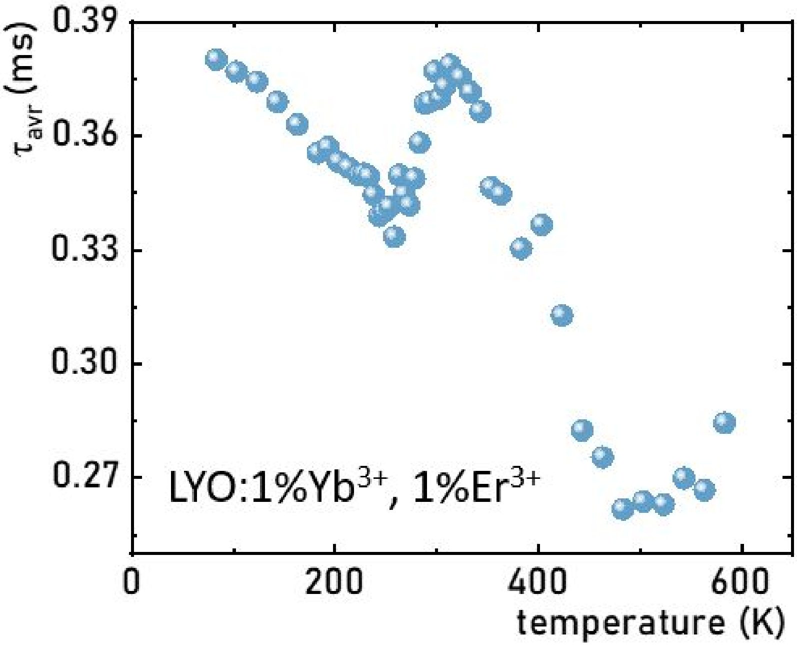

Figure S8. The τ_{avr} of $LiYO_2$:1%Er^{3+},1%Yb^{3+} measured at λ_{em}=670 nm measured as a function of temperature.

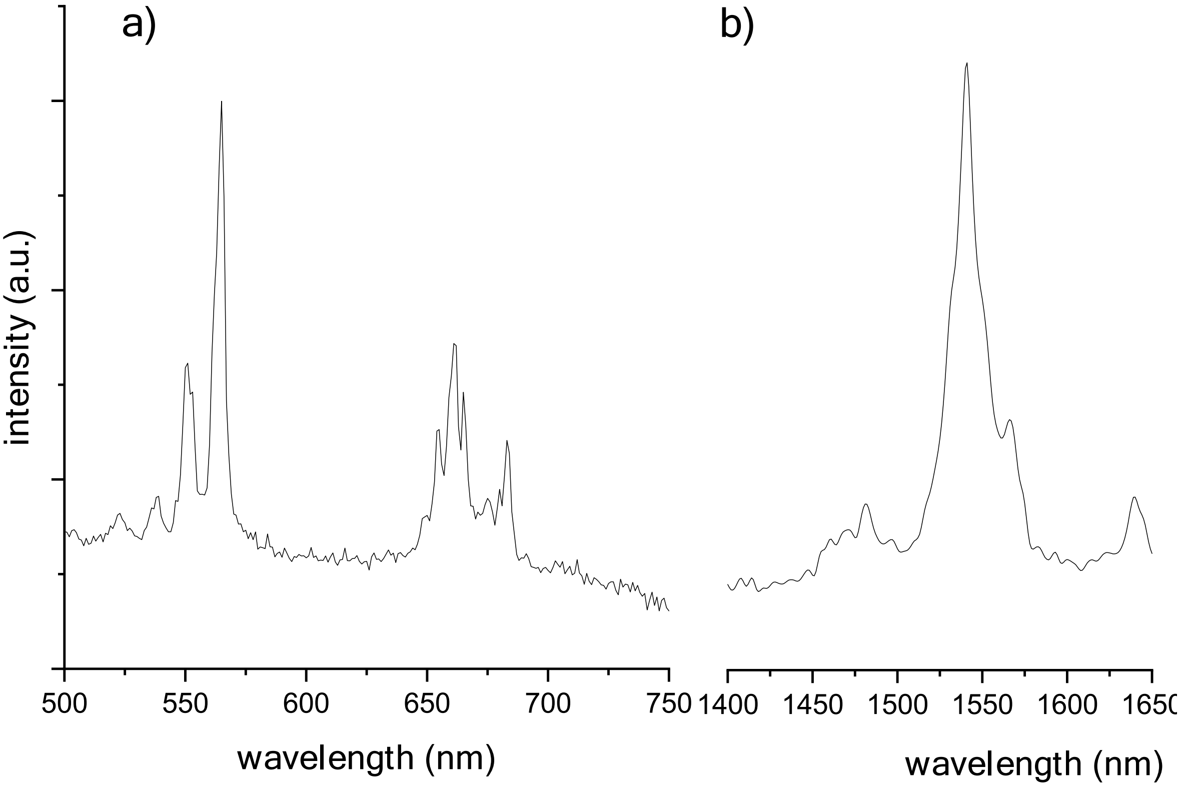

Figure S9. The up-conversion -a) and downshifting-b) emission spectra of $LiYO_2$:1%Er^{3+},1%Yb^{3+} measured in the integration sphere.

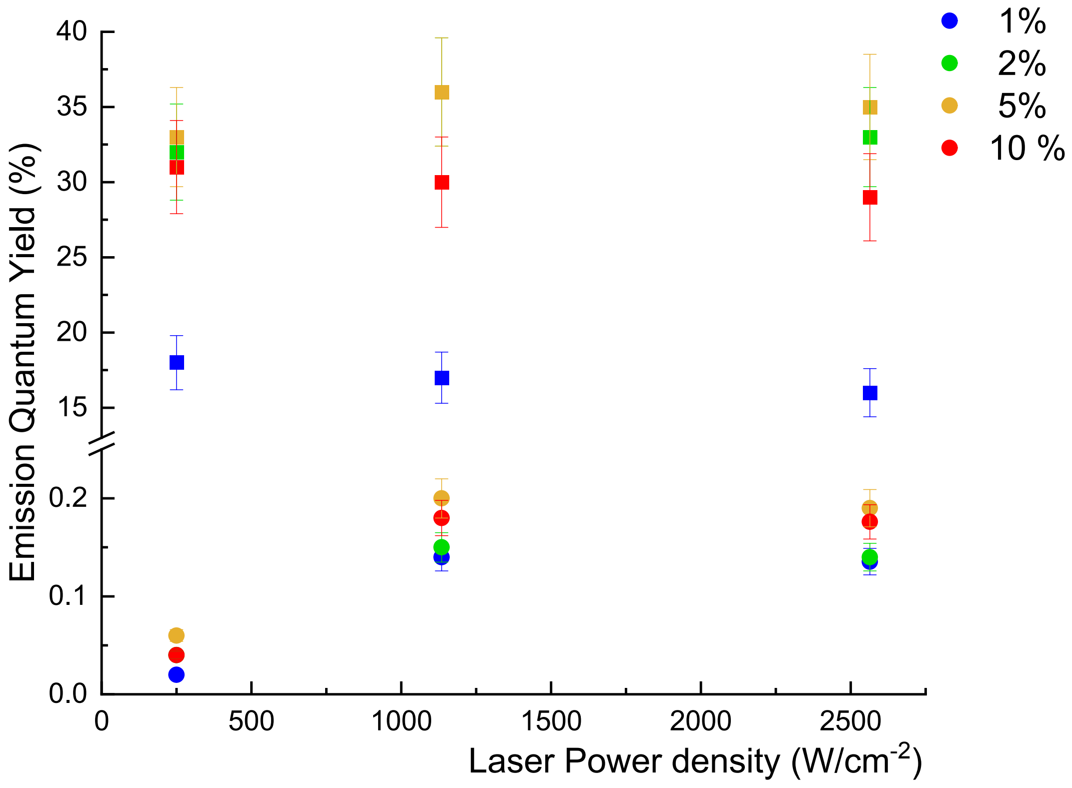

Figure S10. The emission quantum yield for downshifting (upper part) and up-conversion (bottom part) of $LiYO_2$:1%Er^{3+},Yb^{3+} measured upon λ_{exc}=980 nm for different concentration of Yb^{3+} ions.

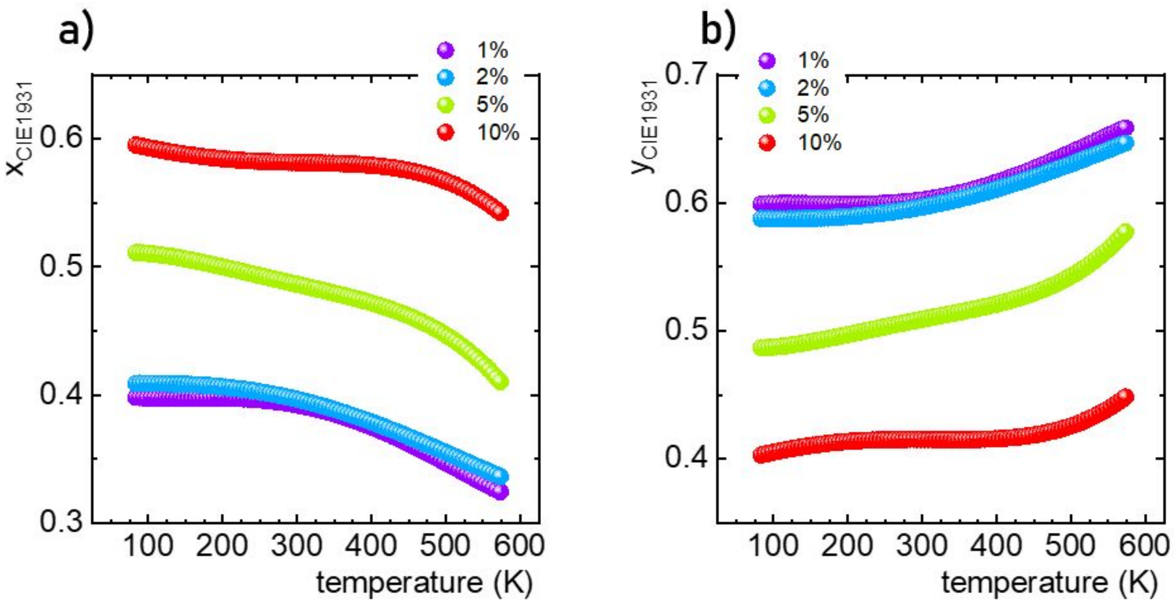

Figure S11. Thermal dependence of x-a) and y-b) chromatic coordinates (1931 CIE) of $LiYO_2$:1%Er^{3+},Yb^{3+} for different concentration of Yb^{3+} ions.

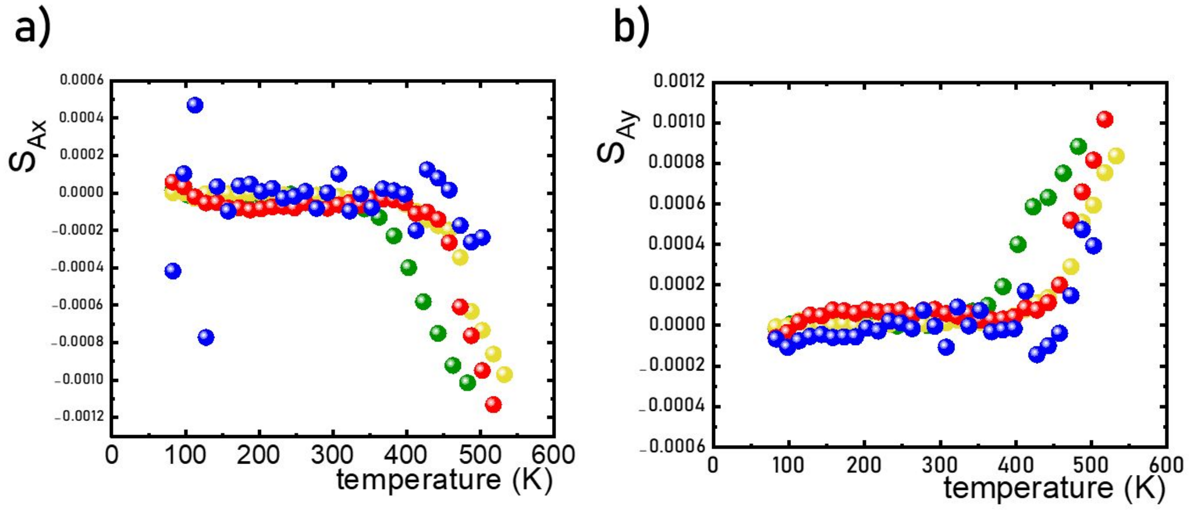

Figure S12. Thermal dependence of absolute sensitivities S_{Ax}-a) and S_{Ay}-b) based on the chromatic coordinates (1931 CIE) of $LiYO_2$:1%Er^{3+},Yb^{3+} for different concentration of Yb^{3+} ions.

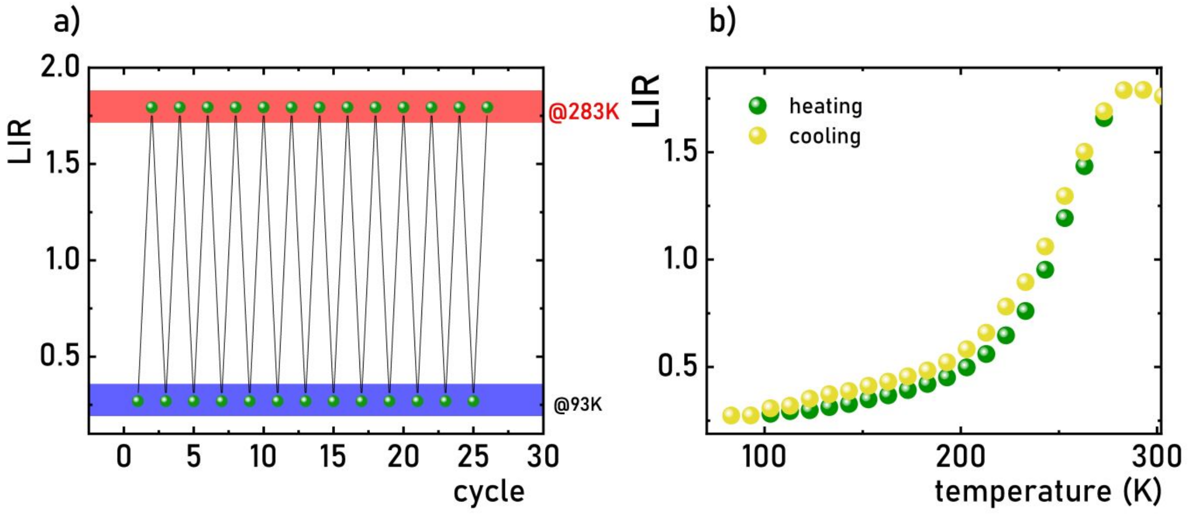

Figure S13. The LIR of $LiYO_2$:1%Er^{3+}, 1%Yb^{3+} measured withing heating-cooling cycles at 93 and 283 K-a) and thermal dependence of this LIR within whole thermal range -b).
